# Design, Synthesis, and Anticancer Evaluation of New Small-Molecule EGFR Inhibitors Targeting NSCLC and Breast Cancer

**DOI:** 10.3390/ijms26157065

**Published:** 2025-07-22

**Authors:** Belgin Sever, Masami Otsuka, Mikako Fujita, Halilibrahim Ciftci

**Affiliations:** 1 Department of Pharmaceutical Chemistry, Faculty of Pharmacy, Anadolu University, Eskisehir 26470, Türkiye; 2 Medicinal and Biological Chemistry Science Farm Joint Research Laboratory, Faculty of Life Sciences, Kumamoto University, Kumamoto 862-0973, Japan; motsuka@gpo.kumamoto-u.ac.jp (M.O.); mfujita@kumamoto-u.ac.jp (M.F.); 3 Department of Drug Discovery, Science Farm Ltd., Kumamoto 862-0976, Japan; 4 Department of Molecular Biology and Genetics, Burdur Mehmet Akif Ersoy University, Istiklal Campus, Burdur 15030, Türkiye; 5 Department of Bioengineering Sciences, Izmir Katip Celebi University, Izmir 35620, Türkiye

**Keywords:** NSCLC, breast cancer, EGFR, cytotoxicity, apoptosis, chalcone, pyrazoline, thiazole, small-molecule inhibitors, in silico studies

## Abstract

EGFR is the most frequently altered driver gene in non-small-cell lung cancer (NSCLC), and its overexpression is also associated with breast cancer. In the present study, we synthesized 18 new compounds (**B-1**, **B-2**, **B-6**, **B-7**, and **BP-1**–**14**). The cytotoxicity of these compounds was evaluated in A549 NSCLC and MCF-7 breast cancer cells, as well as in Jurkat cells and PBMCs (healthy). The most potent compounds were further examined for their ability to induce apoptosis in A549 and MCF-7 cells, as well as their EGFR inhibitory activity. Molecular docking was conducted at the ATP-binding site of EGFR, and key pharmacokinetic and toxicity parameters were predicted in silico. **B-2** demonstrated the strongest cytotoxicity against A549 and MCF-7 cells (IC_50_ = 2.14 ± 0.83 μM and 8.91 ± 1.38 μM, respectively), displaying selective cytotoxicity between Jurkat cells and PBMCs (SI = 23.2). **B-2** induced apoptosis in A549 and MCF-7 cells at rates of 16.8% and 4.3%, respectively. **B-2** inhibited EGFR by 66% at a 10 μM concentration and showed a strong binding affinity to the ATP-binding site of EGFR. Furthermore, **B-2** exhibited drug-like characteristics and was not identified as carcinogenic, genotoxic, or mutagenic. **B-2** shows promise as an apoptosis inducer and EGFR inhibitor for future anti-NSCLC and anti-breast cancer research.

## 1. Introduction

Protein kinases, which transfer high-energy phosphate groups from adenosine triphosphate (ATP) to the hydroxyl groups of serine, threonine, or tyrosine residues, play crucial roles in various cellular processes, including development, proliferation, apoptosis, and signal transduction. Based on their amino acid substrate specificity, protein kinases can be classified into three main groups: serine/threonine kinases, tyrosine kinases, and dual-specificity kinases, which are capable of phosphorylating serine, threonine, or tyrosine residues. Among these, tyrosine kinases-classified as either receptor tyrosine kinases (RTKs) or non-receptor tyrosine kinases (NRTKs)-specifically mediate the phosphorylation of tyrosine residues on cellular proteins [1,2]. The RTK family comprises a plethora of cell surface receptors, which are susceptible to growth factors, hormones, and cytokines, and consequently regulate a myriad of vital cellular and metabolic signalling pathways [3]. Epidermal Growth Factor Receptor (EGFR, ErbB1 or HER-1) is a member of the ErbB family of RTKs, which also includes HER-2 (ErbB2), HER-3 (ErbB3), and HER-4 (ErbB4). The composition of EGFR structure is as follows: an extracellular ligand-binding domain, a hydrophobic transmembrane region, and an intracellular domain with tyrosine kinase activity, and a carboxy-terminal region. EGFR plays a central role in regulating key downstream signalling activation of the RAS-MAP kinase pathways, PI3K-AKT-mTOR and JAK-STAT, thereby controlling essential cellular processes including proliferation, survival, differentiation, adhesion, and migration. Its aberrant activation—through gene mutations, amplifications, or protein overexpression—serves as a key driving mechanism in various solid tumours, particularly in non-small cell lung cancer (NSCLC), which accounts for 80–90% of all lung cancer cases, as well as in breast cancer [4,5,6,7,8,9].

**Figure 1:**
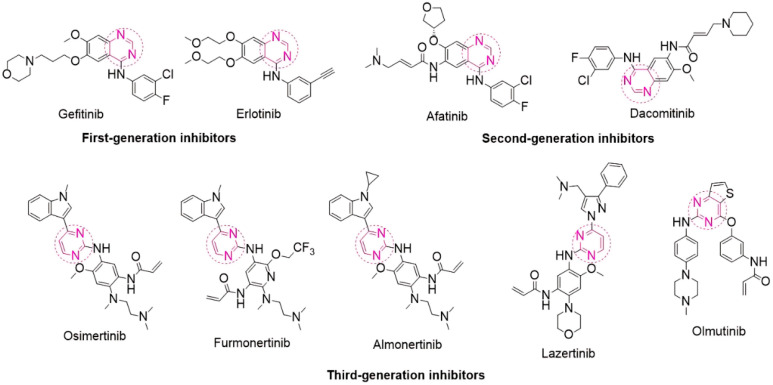
The chemical structures of first-, second- and third-generation EGFR TKIs.

Based on the report of the World Health Organization’s (WHO) cancer agency, the International Agency for Research on Cancer (IARC), lung cancer leads with 2.5 million new cases, followed by breast cancer with 2.3 million diagnoses [10,11]. Currently, a wide range of therapeutic regimens is available for NSCLC, and the development of novel therapies has significantly improved response rates and overall survival in advanced stages, offering the potential for long-term durable remission [12]. Tyrosine kinase inhibitors (TKIs) are often really effective treatments for patients with NSCLC, especially those with tumours that have activating mutations in the EGFR kinase domain [13]. Most first- and second-generation inhibitors contain a quinazoline core, a bicyclic heterocycle consisting of a benzene ring fused with a pyrimidine ring. Gefitinib (Figure 1) and erlotinib (Figure 1) are first-generation EGFR TKIs that selectively and reversibly block ATP binding. In contrast, second-generation EGFR TKIs—such as afatinib and dacomitinib (Figure 1)—bind irreversibly to the C797 residue within the ATP-binding site, effectively inhibiting EGFR autophosphorylation. The emergence of the T790M mutation following treatment with first- and second-generation EGFR TKIs led to the development of third-generation irreversible EGFR TKIs, such as osimertinib, furmonertinib, almonertinib, and lazertinib (Figure 1). The enhanced potency of osimertinib and other third-generation TKIs is attributed to the combination of a covalent warhead and a pyrimidine core, which together enable stronger binding and the irreversible inhibition of EGFR kinase activity [14,15,16,17,18,19]. Olmutinib (Figure 1), which was granted breakthrough therapy designation by the United States Food and Drug Administration (FDA) in 2015 for the treatment of NSCLC, possesses a thieno[3,2-d]pyrimidine core [20,21]. However, the emergence of an additional tertiary C797S resistance mutation has limited the efficacy of third-generation EGFR TKIs, underscoring the need for the development of more effective new small-molecule inhibitors [22].

**Figure 2:**
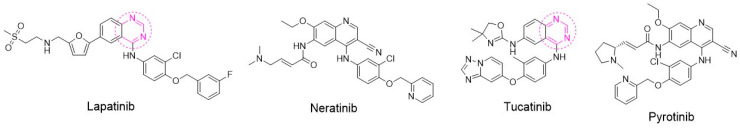
The chemical structures of EGFR TKIs effective against breast cancer.

Breast cancer is classified based on molecular characteristics, including the expression of estrogen receptor (ER), progesterone receptor (PR), HER-2, EGFR, the cell proliferation marker Ki-67, and basal cytokeratins [23,24]. The conventional options of treatment for breast cancer include local treatments such as surgery and radiation therapy, as well as systemic treatments like chemotherapy, endocrine therapy, targeted therapy, and immunotherapy. The selection of each treatment option is based on factors such as hormone receptor status, HER-2 protein expression, overall health, menopausal status, and the stage of the disease [25]. Research has emphasized the potential of anti-EGFR targeted therapies, particularly when combined with monoclonal antibodies, to improve outcomes in the treatment of breast cancer. Currently, the TKIs approved for treating breast cancer are lapatinib, neratinib, tucatinib, and pyrotinib (Figure 2) [26]. Tucatinib is a highly selective inhibitor specifically targeting HER-2, while neratinib and pyrotinib are irreversible TKIs with broader and less selective HER-2 activity. In comparison, lapatinib functions as a dual-action TKI, inhibiting the phosphorylation and activation of both EGFR and HER-2 [27,28]. Despite high levels of EGFR expression in breast cancer, EGFR TKIs have shown limited effectiveness, highlighting the need for the development of new small-molecule drug candidates [29,30].

**Figure 3:**
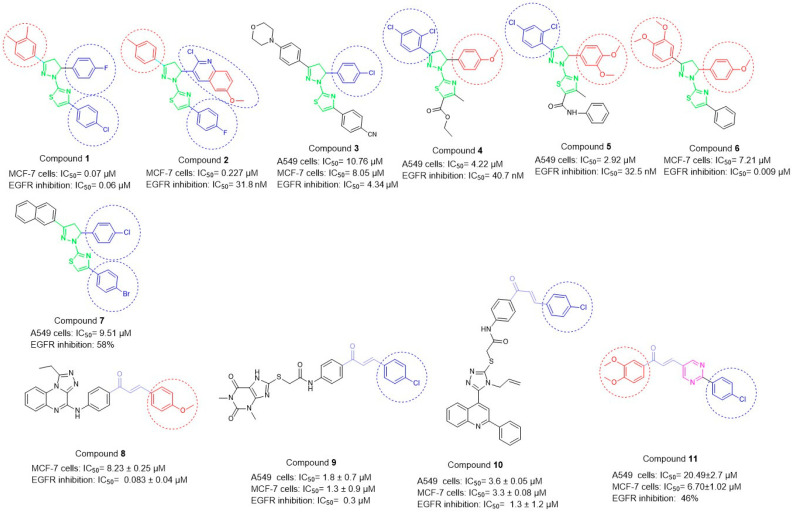
The chemical structures of potential EGFR-TKIs bearing pyrazoline–thiazole and chalcone structures (Pyrazoline–thiazole: green; halogen-substituted aryl groups: blue, and methyl/methoxy-substituted aryl groups: red). Compounds **1** [31], **2** [32], **3** [33], **4** [34], **5** [34], **6** [35], **7** [36], **8** [37], **9** [38], **10** [39], and **11** [11].

The hybridizing pyrazoline and thiazole scaffolds has proven effective in developing novel conjugates with targeted anticancer properties, including EGFR-specific activity against NSCLC and/or breast cancer (Figure 3) [31,32,33,34,35,36]. Among them, compounds **3** and **7** were synthesized by our group, while compounds **1, 2, 4, 5,** and **6** were prepared by other research groups. Chalcones, which serve as precursors for pyrazolines, have also been reported to exhibit potential anticancer activity against EGFR-driven NSCLC and/or breast cancer (Figure 3). Of these, compound **11** was synthesized by our team, whereas compounds **8, 9** and **10** were developed by other research groups [11,37,38,39].

Based on the aforementioned data, the most effective EGFR-TKIs generally feature chalcone- and pyrazoline–thiazole-based structures, substituted with halogen atoms and/or aryl groups bearing methyl or methoxy substituents. In this study, we designed and synthesized 18 novel compounds incorporating 2-(4-chlorophenyl)pyrimidin-5-yl along with tolyl or methoxyphenyl groups. The series includes chalcones (**B-1** and **B-2**), pyrazoline–carbothioamides (**B-6** and **B-7**), and pyrazoline–thiazole hybrids (**BP-1**–**14**). Subsequently, we assessed the anticancer activity of these compounds against A549 NSCLC and MCF-7 breast cancer cell lines, as well as their selective cytotoxicity between Jurkat leukemic T cells and peripheral blood mononuclear cells (PBMCs) (healthy). The most potent compound was further investigated to understand its anticancer mechanisms, including its ability to induce apoptosis in NSCLC and breast cancer cells and its inhibition of EGFR. Finally, molecular docking was conducted to examine its interaction with the ATP-binding site of EGFR, and several Absorption, Distribution, Metabolism, Excretion, and Toxicity (ADMET) parameters were predicted through in silico analysis.

## 2. Results

### 2.1. Chemistry

**Scheme 1:**
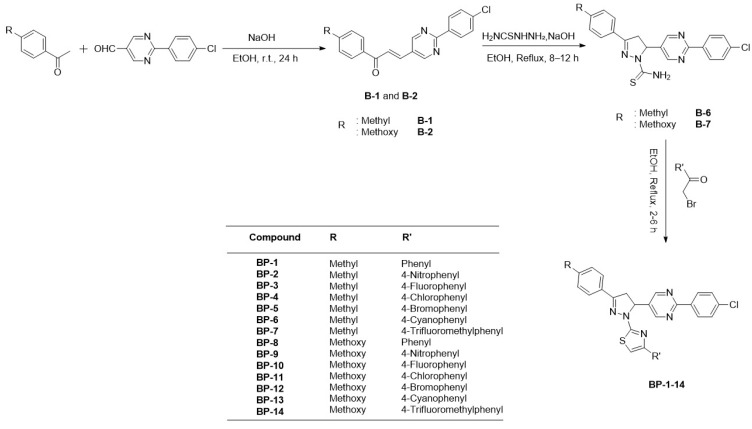
Synthetic route of **B-1**, **B-2**, **B-6**, **B-7**, and **BP-1**–**14**.

In this study, the synthesis commenced with **B-1** and **B-2**, prepared through the Claisen–Schmidt condensation of 1-(4-tolyl/4-methoxyphenyl)ethan-1-one with 2-(4-chlorophenyl)pyrimidine-5-carbaldehyde under basic conditions. These intermediates were then reacted with thiosemicarbazide in the presence of NaOH to yield **B-6** and **B-7**. The final compounds (**BP-1**–**14**) were obtained via Hantzsch thiazole synthesis [40], involving the condensation of α-haloketones (2-bromo-1-arylethanones) with the thioamides **B-6** and **B-7** (Scheme 1).

### 2.2. Anticancer Activity

**Figure 4:**
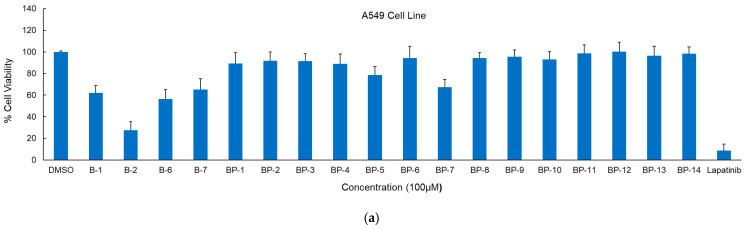

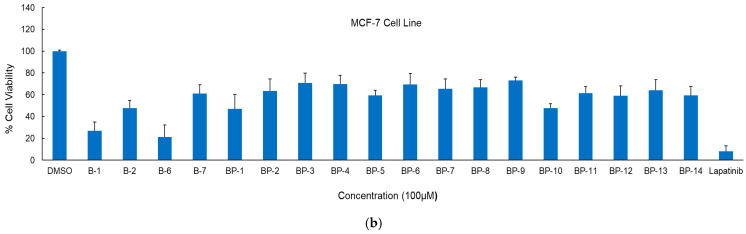
The level of cell toxicity of **B-1**, **B-2**, **B-6**, **B-7**, and **BP-1**–**14** against A549 (**a**) and MCF-7 (**b**) cells at a concentration of 100 µM is demonstrated in comparison to lapatinib. The culture time was 72 h, and the experiments were performed in triplicate. The data are presented as mean values with a maximum deviation of 15%.

The newly synthesized **BP-1**–**14**, along with intermediates **B-1**, **B-2**, **B-6**, and **B-7** were assessed for their cytotoxic effects on A549 and MCF-7 cell lines using the MTT (3-(4,5-dimethyl-2-thiazolyl)-2,5-diphenyltetrazolium bromide) assay, with lapatinib serving as a reference compound. The anticancer potential of **BP-1**–**14**, **B-1**, **B-2**, **B-6**, and **B-7** was initially evaluated in triplicate at a concentration of 100 µM against A549 and MCF-7 cells (Figure 4a,b). Among the tested compounds, **B-2** exhibited the most pronounced cytotoxic effect, reducing A549 cell viability by 73%, followed by **B-6** (44%), **B-1** (38%), and **B-7** (35%). Among **BP-1**–**14**, **BP-7** and **BP-5** showed the highest inhibitory activity, decreasing cell viability by 33% and 21%, respectively (Figure 4a). On the other hand, most of the tested compounds demonstrated notably stronger cytotoxicity against MCF-7 cells at a concentration of 100 µM. Among them, **B-6** showed the highest anticancer activity with 79% inhibition, followed by **B-1** (73%), **B-2** (52%), and **B-7** (39%). **BP-1**–**14** showed moderate cytotoxicity, with inhibition ranging from 27% to 53% (**BP-1** and **BP-10**) (Figure 4b).

As **B-1**, **B-2**, **B-6**, **BP-1**, and **BP-10** revealed over 50% inhibition against A549 and/or MCF-7 cells at a concentration of 100 µM, they were further tested at five different concentrations (1, 3, 10, 30, and 100 µM), with each dose assessed in triplicate, and IC_50_ values were subsequently calculated (Table 1). **B-2** demonstrated superior anticancer activity against both A549 and MCF-7 cell lines, with IC_50_ values of 2.14 ± 0.83 µM and 8.91 ± 1.38 µM, respectively, surpassing the efficacy of lapatinib, which showed IC_50_ values of 18.21 ± 3.25 µM for A549 cells and 9.71 ± 1.12 µM for MCF-7 cells (Table 1). On the other hand, **B-1** and **B-6** emerged as the most promising anticancer agents in this series, with IC_50_ values of 6.10 ± 1.26 µM and 6.52 ± 0.97 µM, respectively. The anti-lung cancer effects of **B-1** and **B-6** were not found to be statistically significant. Furthermore, **BP-1** and **BP-10** showed no significant anticancer activity against either A549 or MCF-7 cells (Table 1).

As illustrated in Table 1, the anticancer selectivity of compounds **B-1**, **B-2**, and **B-6** was assessed by comparing their effects on Jurkat T cells and healthy PBMCs. The selectivity index (SI)—calculated by dividing the IC_50_ value for PBMCs by the corresponding IC_50_ for Jurkat cells—was determined to be 57.08 for **B-1**, 23.20 for **B-2**, and 7.49 for **B-6**. These SI values are notably higher than that of lapatinib (SI = 7.72), suggesting that **B-1** and **B-2** exhibit superior selectivity.

**Figure 5:**
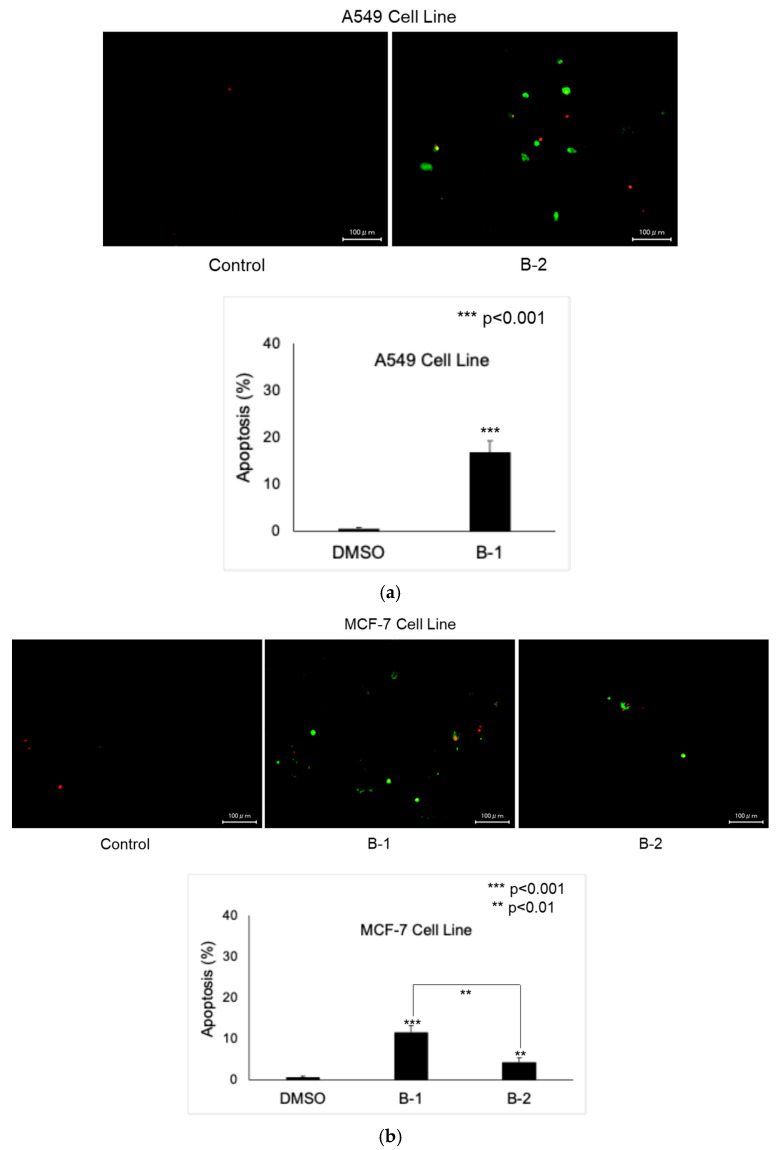
The changes observed in A549 (**a**) and MCF-7 (**b**) cells following 24 h of treatment with either the control or **B-1** and **B-2** (Statistical significance is indicated as follows: *** *p* < 0.001; ** *p* < 0.01).

Given that **B-2** and **B-1** demonstrated the highest effectiveness and selectivity as anticancer agents against A549 and MCF-7 cells, respectively, apoptosis levels in these cell lines were evaluated using Annexin V/ethidium homodimer-III staining. In this method, green staining indicates apoptotic cells, red indicates necrotic cells, and yellow represents cells in the late stages of apoptosis. This staining was analyzed using a fluorescence microscope. The results showed that **B-2** induced apoptosis in 16.8% of A549 cells (Figure 5a), whereas **B-1** triggered apoptosis in 11.6% of MCF-7 cells compared to the control (Figure 5b). Furthermore, the apoptotic effect of **B-2** on MCF-7 cells was assessed, showing that 4.3% of the cells underwent apoptosis after treatment, a result that was statistically significant (Figure 5b). The apoptotic responses induced by **B-2** in A549 cells and **B-1** in MCF-7 cells were found to be highly statistically significant (Figure 5a,b). The difference in apoptotic behaviour between **B-1** and **B-2** in MCF-7 cells was found to be statistically significant (Figure 5b).

**Figure 6:**
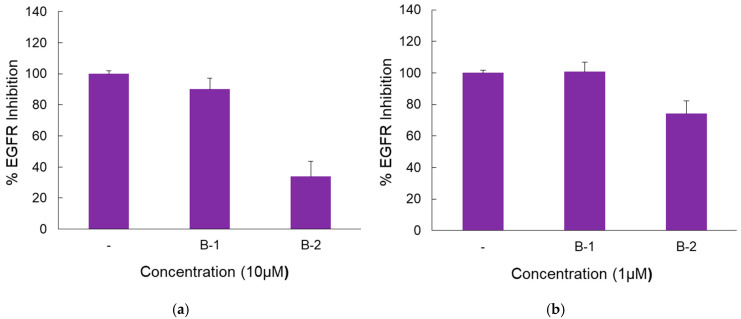
EGFR inhibition by **B-1** and **B-2** at concentrations of 10 μM (**a**) and 1 μM (**b**).

Overexpression or mutation of the EGFR gene markedly enhances cell growth and proliferation, particularly in NSCLC [41] and breast cancer [32], making EGFR a well-established and effective therapeutic target for these diseases. Due to their potent activity against NSCLC and breast cancer, **B-1** and **B-2** were further examined for their potential to inhibit EGFR. The findings revealed that **B-2** inhibited EGFR activity by 66% at a concentration of 10 μM and by 26% at 1 μM. In contrast, **B-1** showed no inhibitory effect on EGFR at either concentration (Figure 6).

### 2.3. Molecular Docking and In Silico Pharmacokinetic Estimation

**Figure 7:**
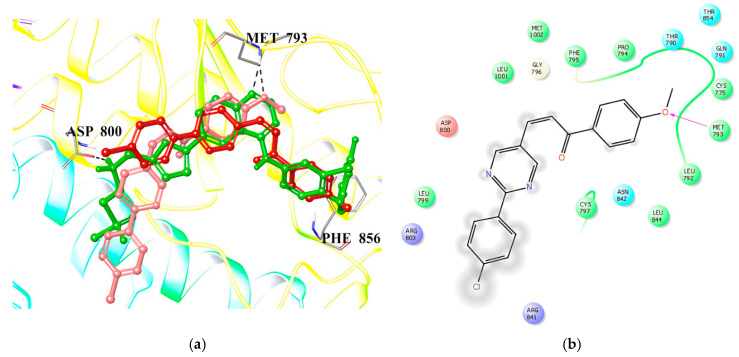

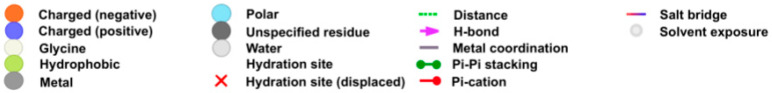
Docking poses of **B-1, B-2** and lapatinib—shown in red, pink and green, respectively (**a**). Docking interactions of **B-2** within the ATP-binding cleft of the EGFR (**b**).

A molecular docking study was conducted for **B-1** and **B-2** to assess their ability to bind to the ATP-binding site of EGFR (PDB ID: 1XKK) [42], in comparison with lapatinib, using the Maestro software [43]. The results demonstrated that both **B-1** and **B-2** occupied the ATP-binding site of EGFR with high affinity (Figure 7a). The calculated docking scores were −9.03 kcal/mol for **B-2** and −8.94 kcal/mol for **B-1**, while lapatinib showed a more favourable score of −12.20 kcal/mol. **B-2** was able to form a hydrogen bond with the key residue Met793 via its 4-methoxyphenyl substitution (Figure 7b). In contrast, **B-1** did not establish any significant interactions.

**B-1** and **B-2** were assessed using in silico profiling for various ADMET properties through the QikProp module [44] and the ADMETlab 3.0 web tool [45]. The evaluation covered parameters such as the octanol/water partition coefficient (QPlogPo/w), aqueous solubility (QPlogS), human serum albumin binding (QPlogKhsa), and percent human oral absorption (HOA). Additionally, their compliance with Lipinski’s Rule of Five and Jorgensen’s Rule of Three was examined using QikProp, while AMES toxicity, carcinogenicity, rat oral acute toxicity, and genotoxicity were analyzed using ADMETlab 3.0. Moreover, the potential of **B-1** and **B-2** to inhibit major cytochrome P450 (CYP) enzymes—specifically CYP1A2, CYP2C19, CYP2C9, CYP2D6, and CYP3A4—as well as their ability to cross the blood–brain barrier (BBB), was evaluated using the SwissADME web tool [46].

**B-1** and **B-2** exhibited strong pharmacokinetic profiles, with all evaluated parameters falling within the recommended ranges. For **B-1**, the values were: QPlogPo/w at 4.802, QPlogS at −5.407, and QPlogKhsa at 0.503. For **B-2**, the corresponding values were 4.421, −4.948, and 0.241. These results lie within the acceptable intervals of −2 to 6.5 for QPlogPo/w, −6.5 to 0.5 for QPlogS, and −1.5 to 1.5 for QPlogKhsa. **B-1** and **B-2** also showed excellent HOA, each scoring 100% on a 0–100% scale, where values above 80% are considered high and below 25% low. **B-2** fully complied with both Lipinski’s Rule of Five and Jorgensen’s Rule of Three. **B-1** met all the criteria of Lipinski’s rule and had only one minor violation of Jorgensen’s rule, which remains within acceptable limits.

**B-1** exhibited excellent safety profiles across all evaluated parameters, with values of 0.177 for AMES toxicity, 0.334 for carcinogenicity, 0.187 for rat oral acute toxicity, and 0.303 for genotoxicity. Similarly, **B-2** showed excellent results for AMES toxicity (0.257) and rat oral acute toxicity (0.193). However, its carcinogenicity (0.429) and genotoxicity (0.356) were classified as medium. According to the classification criteria, optimal values fall into Category 0 for AMES toxicity, carcinogenicity, and genotoxicity, and Category 0 for rat oral acute toxicity, which corresponds to doses greater than 500 mg/kg.

**Figure 8:**
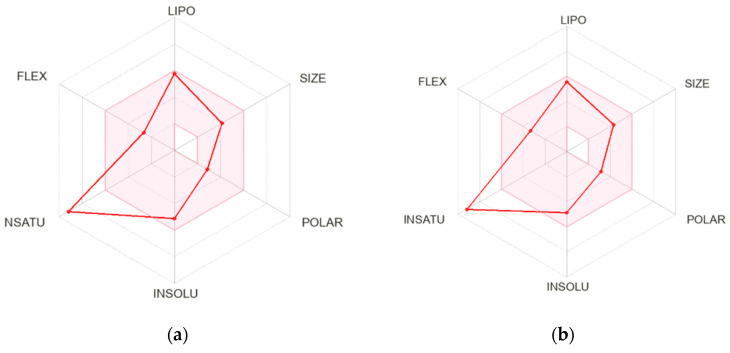
The bioavailability radars for **B-1** (**a**) and **B-2** (**b**), generated using the SwissADME web tool.

The pink region of the Bioavailability Radar (Figure 8) highlights optimal ranges for oral bioavailability parameters, including saturation (INSATU), size (SIZE), polarity (POLAR), solubility (INSOLU), lipophilicity (LIPO), and flexibility (FLEX). Both **B-1** and **B-2** fell outside the ideal range only for saturation, while all other parameters remained within the pink area. In terms of cytochrome P450 enzyme inhibition, **B-1** is predicted to inhibit CYP1A2, CYP2C19, and CYP2C9, but not CYP3A4 or CYP2D6. **B-2**, on the other hand, inhibits CYP1A2, CYP2C19, CYP2C9, and CYP3A4, but not CYP2D6. These interactions suggest that **B-1** and **B-2** may pose a risk of potential drug–drug or drug–food interactions.

**Figure 9:**
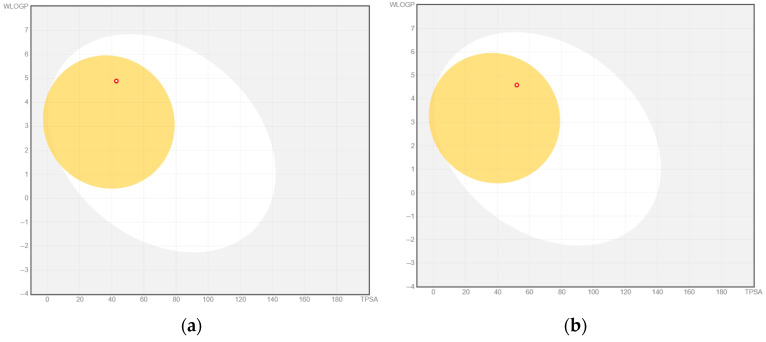
The BOILED-Egg models **B-1** (**a**) and **B-2** (**b**) from the SwissADME web tool.

The BOILED-Egg model (Figure 9) is used to predict a molecule’s potential for passive gastrointestinal absorption and BBB penetration. According to the results, **B-1** and **B-2** were predicted to penetrate the brain, as indicated by their position in the yellow region. Additionally, it is not expected to act as substrates for P-glycoprotein (red dot), suggesting a reduced likelihood of being expelled by efflux mechanisms in cancer cells, which may lower the risk of resistance.

## 3. Discussion

EGFR is one of the most significant oncogenic targets identified in NSCLC, and its discovery marked a major milestone in the development of EGFR-targeted therapies. However, as these therapies have become more widely used, several limitations have surfaced. Persistent challenges include the emergence of complex and evolving resistance mechanisms, primary treatment failure in some patients, and significant inequalities in drug accessibility—all of which remain major hurdles for both researchers and clinicians. In contrast, although EGFR is highly expressed in breast cancer, EGFR inhibitors have shown limited clinical success in this context. This discrepancy emphasizes the need to explore the mechanisms responsible for resistance to EGFR-targeted therapies in breast cancer. Therefore, the development of novel small-molecule inhibitors capable of effectively targeting EGFR-driven NSCLC and breast cancer remains a critical area of research.

The most effective third-generation EGFR TKIs are characterized by the presence of a pyrimidine core (Figure 1). Chalcone derivatives have also been reported for their potential to target EGFR-driven NSCLC and/or breast cancer (Figure 3). In our recent study, we demonstrated the significant therapeutic contribution of both the pyrimidine core and chalcone moiety in combating EGFR-driven NSCLC and breast cancer. These promising findings motivated us to further optimize compound **11** (Figure 3) to enhance its mechanistic anticancer activity. To this end, we designed and synthesized a series of new compounds, including chalcones (**B-1** and **B-2**), pyrazoline–carbothioamides (**B-6** and **B-7**), and pyrazoline–thiazole hybrids (**BP-1**–**14**). Their anticancer activity was evaluated against A549 and MCF-7 cell lines. Furthermore, the most active compounds were assessed for their pro-apoptotic effects in both cell lines and their potential to inhibit EGFR activity. In silico analyses were also performed to investigate the binding affinity of the lead compounds to the ATP-binding site of EGFR, as well as their key pharmacokinetic and toxicity profiles.

The MTT assay results revealed that the chalcone (**B-1** and **B-2**) and pyrazoline–carbothioamide (**B-6**) moieties contributed more significantly to anticancer activity than the pyrazoline–thiazole framework. The 4-methoxy substitution, as shown in **B-2**, enhanced the anti-NSCLC activity compared to the 4-tolyl substitution in **B-1**. Although **BP-1**–**14** did not show significant anticancer activity against A549 cells, the most effective compounds, **BP-1** and **BP-5**, highlighted the importance of a 4-tolyl substitution at the 3rd position of the pyrazoline ring, as well as phenyl and 4-bromophenyl substitutions at the 4th position of the thiazole ring, respectively. On the other hand, **B-1** and **B-6** exhibited comparable anti-breast cancer activity, with their cytotoxic effects against MCF-7 cells being greater than that of **B-2**. This outcome indicated that 4-tolyl substitution of **B-1** and **B-6** made a great impact on anti-breast cancer activity compared to 4-methoxy substitution of **B-2**. Another notable result from the MTT assay is that **B-2** was the only compound to exhibit significant dual activity against both NSCLC and breast cancer cell lines. Despite **BP-1**–**14** did not exhibit significant anti-breast cancer activity overall, **BP-1** and **BP-10** showed relatively higher anticancer effects, suggesting that phenyl and 4-fluorophenyl substitutions at the 4th position of the thiazole ring may enhance activity. These findings align with earlier studies on the most effective chalcone- and pyrazoline–thiazole-based EGFR TKIs (Figure 3), emphasizing the crucial role of halogen-substituted and methyl/methoxy-substituted aryl groups in increasing activity.

**B-1** and **B-2** showed cytotoxic activity against Jurkat cells while displaying reduced toxicity toward healthy PBMCs, underscoring their selective cytotoxicity. The strong anticancer effects of **B-1** and **B-2** against Jurkat cells further imply that these compounds might act on other kinases beyond EGFR.

**B-2** triggered apoptosis in both NSCLC and breast cancer cells, with a significantly greater effect observed in NSCLC cells. Breast cancer cells treated with **B-1** showed a more pronounced level of apoptosis than those treated with **B-2**, indicating that the 4-tolyl substitution in **B-1** markedly enhances its pro-apoptotic activity in breast cancer cells compared to **B-2**. Furthermore, **B-2** exhibited strong inhibition of EGFR at a concentration of 10 µM, highlighting its EGFR-targeted apoptotic activity in both cells. However, **B-1** failed to inhibit EGFR at concentrations of 1 µM and 10 µM, indicating that its apoptotic effects in MCF-7 cells may occur through an EGFR-independent pathway. Molecular docking studies confirmed that the 4-methoxyphenyl substitution in **B-2** is crucial for forming key hydrogen bonds within the ATP binding site of EGFR.

In silico pharmacokinetic analysis revealed that **B-1** and **B-2** exhibit drug-like properties, including favourable lipophilicity, good aqueous solubility, and strong binding affinity to human serum albumin. Furthermore, in silico toxicity evaluations indicated that both compounds are non-carcinogenic, non-mutagenic (negative in the AMES toxicity), and non-genotoxic. One of the primary locations for metastasis in NSCLC is the brain, making the ability of therapeutic agents to cross BBB critically important. **B-1** and **B-2** have demonstrated the capability to penetrate the BBB. **B-1** and **B-2** may be metabolized by several tested CYP enzymes, which could lead to potential drug–drug interactions.

## 4. Materials and Methods

### 4.1. Chemistry

We purchased all reagents commercially and used them without further purification, unless otherwise specified. Thin-layer chromatography (TLC) was performed on silica gel 60 F254 aluminum sheets (Merck, Darmstadt, Germany). Proton (^1^H) and carbon (^13^C) nuclear magnetic resonance (NMR) spectra were recorded using a Bruker NMR spectrometer (Bruker, Billerica, MA, USA), and high-resolution mass spectra (HRMS) were obtained using a JEOL JMS-700 Station/JMS-BU-20-GCmate (JEOL, Akishima, Tokyo, Japan).

#### 4.1.1. Synthesis of (E)-3-(2-(4-Chlorophenyl)pyrimidin-5-yl)-1-(4-methylphenyl)-2-propen-1-one (**B-1**) and (E)-3-(2-(4-Chlorophenyl)pyrimidin-5-yl)-1-(4-methoxyphenyl)-2-propen-1-one (**B-2**)

A mixture of 2-(4-chlorophenyl)pyrimidine-5-carbaldehyde (1 mmol), 1-(4-methyl/methoxyphenyl)ethan-1-one (1 mmol), and NaOH (1.1 mmol) was prepared in ethanol and stirred at room temperature for 24 h. Upon completion, as confirmed by thin-layer chromatography (TLC), the reaction mixture was poured onto crushed ice to induce precipitation. The resulting solid was filtered, washed with water, and dried. Final recrystallization of the product was carried out using ethanol [11,36].

**B-1:** ^1^H NMR (500 MHz, *CDCl*_3_) *δ* (ppm): 2.47 (3H, s, CH_3_), 7.34 (2H, d, *J* = 9.05 Hz, aromatic), 7.48 (2H, d, *J* = 9.6 Hz, aromatic), 7.69 (1H, *J* = 14.6 Hz, C_2_-H), 7.76 (1H, *J* = 15.6 Hz, C_3_-H), 7.96 (2H, *J* = 7.6 Hz, aromatic), 8.45 (2H, d, *J* = 9.1 Hz, aromatic), 9.03 (2H, s, aromatic). ^13^C NMR (125 MHz, *CDCl*_3_) *δ* (ppm): 21.9 (CH_3_), 124.5 (CH, C_2_-H), 126.6 (C, aromatic), 128.8 (2CH, aromatic), 129.1 (2CH, aromatic), 129.6 (2CH, aromatic), 129.9 (2CH, aromatic), 130.7 (C, aromatic), 136.9 (C, aromatic), 137.7 (C, aromatic), 140.1 (C, aromatic), 142.0 (CH, C_3_-H), 156.4 (2CH, aromatic), 161.8 (C, aromatic), 188.8 (C, C=O). HRMS (FAB) calcd. for C_20_H_16_ON_2_Cl [M+H]^+^: *m*/*z* = 335.0951; found: 335.0949. (Spectral Data: Supplementary Information. Figures S1–S3).

**B-2:** ^1^H NMR (500 MHz, *CDCl*_3_) *δ* (ppm): 3.91 (3H, s, OCH_3_), 7.02 (2H, d, *J* = 9.7 Hz, aromatic), 7.48 (2H, d, *J* = 9.2 Hz, aromatic), 7.69 (1H, *J* = 15.2 Hz, C_2_-H), 7.75 (1H, *J* = 16.4 Hz, C_3_-H), 8.06 (2H, *J* = 7.6 Hz, aromatic), 8.45 (2H, d, *J* = 9.1 Hz, aromatic), 9.02 (2H, s, aromatic). ^13^C NMR (125 MHz, *CDCl*_3_) *δ* (ppm): 55.7 (OCH_3_), 114.2 (2CH, aromatic), 124.3 (CH, C_2_-H), 126.6 (C, aromatic), 128.8 (2CH, aromatic), 129.9 (2CH, aromatic), 130.9 (2CH, aromatic), 132.9 (C, aromatic), 135.4 (C, aromatic), 136.1 (C, aromatic), 146.5 (CH, C_3_-H), 156.3 (2CH, aromatic), 157.2 (C, aromatic), 163.5 (C, aromatic), 189.1 (C, C=O). HRMS (FAB) calcd. for C_20_H_16_O_2_N_2_Cl [M+H]^+^: *m*/*z* = 351.0900; found: 351.0916. (Spectral Data: Supplementary Information. Figures S4–S6).

#### 4.1.2. Synthesis of 3-(4-Methylphenyl)-5-(2-(4-chlorophenyl)pyrimidin-5-yl)-1-thiocarbamoyl-2-pyrazoline (**B-6**) and 3-(4-methoxyphenyl)-5-(2-(4-chlorophenyl)pyrimidin-5-yl)-1-thiocarbamoyl-2- pyrazoline (**B-7**)

A mixture of **B-1** and **B-2** (3 mmol), thiosemicarbazide (4.5 mmol), and NaOH (3 mmol) in ethanol was refluxed for 8–12 h. After completion, the reaction mixture was poured onto crushed ice and allowed to cool, followed by washing with water. The resulting precipitate was filtered, rinsed with water, and dried. The final product was purified by recrystallization from ethanol [11,36].

**B-6:** ^1^H NMR (500 MHz, *DMSO-d_6_*) *δ* (ppm): 2.37 (3H, s, CH_3_), 3.46 (1H, dd, *J_AB_* = 18.5 Hz, *J_AX_* = 4.7 Hz, C_4_-H_A_ pyrazoline), 3.96 (1H, dd, *J_BA_* = 18.4 Hz, *J_BX_* = 12.2 Hz, C_4_-H_B_ pyrazoline), 6.01 (1H, dd, *J_BX_* = 11.8 Hz, *J_AX_* = 4.5 Hz, C_5_-H_X_ pyrazoline), 7.30 (2H, d, *J* = 8.1 Hz, aromatic), 7.59 (2H, d, *J* = 8.7 Hz, aromatic), 7.80 (2H, d, *J* = 8.3 Hz, aromatic), 7.99 (1H, bs, NH), 8.20 (1H, bs, NH), 8.38 (2H, d, *J* = 8.3 Hz, aromatic), 8.72 (2H, s, aromatic). ^13^C NMR (125 MHz, *DMSO-d_6_*) *δ* (ppm): 21.7 (CH_3_, OCH_3_), 41.8 (CH_2_, C_4_ pyrazoline), 59.5 (CH, C_5_ pyrazoline), 127.2 (2CH, aromatic), 127.9 (2CH, aromatic), 128.9 (2CH, aromatic), 129.3 (2CH, aromatic), 134.2 (C, aromatic), 135.7 (C, aromatic), 135.8 (C, aromatic), 137.6 (C, aromatic), 140.7 (C, aromatic), 155.3 (C, C_3_ pyrazoline), 155.5 (2CH, aromatic), 161.0 (C, aromatic), 176.0 (C, C=S). HRMS (FAB) calcd. for C_21_H_19_N_5_ClS [M+H]+: *m*/*z* = 408.1050; found: 408.1058. (Spectral Data: Supplementary Information. Figures S7–S9).

**B-7:** ^1^H NMR (500 MHz, *DMSO-d_6_*) *δ* (ppm): 3.44 (1H, dd, *J_AB_* = 18.0 Hz, *J_AX_* = 4.2 Hz, C_4_-H_A_ pyrazoline), 3.82 (3H, s, OCH_3_), 3.95 (1H, dd, *J_BA_* = 17.8 Hz, *J_BX_* = 11.4 Hz, C_4_-H_B_ pyrazoline), 6.01 (1H, dd, *J_BX_* = 11.7 Hz, *J_AX_* = 4.3 Hz, C_5_-H_X_ pyrazoline), 7.04 (2H, d, *J* = 8.9 Hz, aromatic), 7.57 (2H, d, *J* = 7.9 Hz, aromatic), 7.85 (2H, d, *J* = 8.9 Hz, aromatic), 7.96 (1H, bs, NH), 8.18 (1H, bs, NH), 8.37 (2H, d, *J* = 9.5 Hz, aromatic), 8.72 (2H, s, aromatic). ^13^C NMR (125 MHz, *DMSO-d_6_*) *δ* (ppm): 41.8 (CH_2_, C_4_ pyrazoline), 56.4 (2CH_3_, OCH_3_), 58.9 (CH, C_5_ pyrazoline), 114.3 (2CH, aromatic), 123.1 (C, aromatic), 128.9 (2CH, aromatic), 129.1 (2CH, aromatic), 129.3 (2CH, aromatic), 134.3 (C, aromatic), 135.7 (C, aromatic), 135.7 (C, aromatic), 155.0 (C, C_3_ pyrazoline), 155.6 (2CH, aromatic), 161.0 (C, aromatic), 161.3 (C, aromatic), 175.3 (C, C=S). HRMS (FAB) calcd. for C_21_H_19_ON_5_ClS [M+H]+: *m*/*z* = 424.0999; found: 424.1000. (Spectral Data: Supplementary Information. Figures S10–S12).

#### 4.1.3. Synthesis of 1-(4-(Aryl)thiazol-2-yl)-3-(4-methyl/methoxyphenyl)-5-(2-(4-chlorophenyl)pyrimidin-5-yl)-2-pyrazoline derivatives (**BP1**–**14**)

**B-6** and **B-7** (0.8 mmol each) were reacted with 2-bromo-1-arylethanone (0.8 mmol) in ethanol under reflux for 2–6 h. After completion, the precipitate was filtered, washed with water, and dried. The final product was purified by recrystallization from ethanol [11,36].

1-(4-Phenylthiazol-2-yl)-3-(4-methylphenyl)-5-(2-(4-chlorophenyl)pyrimidin-5-yl)-2-pyrazoline (**BP-1**): ^1^H NMR (500 MHz, *CDCl*_3_) *δ* (ppm): 3.42 (3H, s, CH_3_), 3.37 (1H, dd, *J_AB_* = 17.4 Hz, *J_AX_* = 7.3 Hz, C_4_-H_A_ pyrazoline), 3.96 (1H, dd, *J_BA_* = 17.3 Hz, *J_BX_* = 12.0 Hz, C_4_-H_B_ pyrazoline), 5.67 (1H, dd, *J_BX_* = 11.5 Hz, *J_AX_* = 7.4 Hz, C_5_-H_X_ pyrazoline), 6.85 (1H, s, aromatic), 7.21–7.26 (4H, m, aromatic), 7.31 (2H, t, *J* = 7.5 Hz, aromatic), 7.43 (2H, d, *J* = 9.2 Hz, aromatic), 7.64–7.69 (3H, m, aromatic), 8.37 (2H, d, *J* = 8.8 Hz, aromatic), 8.37 (2H, s, aromatic). ^13^C NMR (125 MHz, *CDCl*_3_) *δ* (ppm): 22.1 (CH_3_), 42.6 (CH_2_, pyrazoline C_4_), 60.9 (CH, pyrazoline C_5_), 108.1 (CH, thiazole C_5_), 124.1 (2CH, aromatic), 126.3 (2CH, aromatic), 126.5 (2CH, aromatic), 127.9 (CH, aromatic), 128.9 (2CH, aromatic), 129.6 (2CH, aromatic), 129.7 (2CH, aromatic), 132.2 (C, aromatic), 135.7 (C, aromatic), 137.2 (C, aromatic), 140.4 (C, aromatic), 140.9 (C, aromatic), 146.8 (C, aromatic), 149.4 (C, thiazole C_4_), 152.5 (C, pyrazoline C_3_), 156.2 (2CH, aromatic), 163.4 (C, aromatic), 165.4 (C, thiazole C_2_). HRMS (FAB) calcd. for C_29_H_23_N_5_ClS [M+H]+: *m*/*z* = 508.1363; found: 508.1355. (Spectral Data: Supplementary Information. Figures S13–S15).

1-(4-(4-Nitrophenyl)thiazol-2-yl)-3-(4-methylphenyl)-5-(2-(4-chlorophenyl)pyrimidin-5-yl)-2-pyrazoline (**BP-2**): ^1^H NMR (500 MHz, *CDCl*_3_) *δ* (ppm): 2.42 (3H, s, CH_3_), 3.40 (1H, dd, *J_AB_* = 17.4 Hz, *J_AX_* = 7.3 Hz, C_4_-H_A_ pyrazoline), 3.99 (1H, dd, *J_BA_* = 17.5 Hz, *J_BX_* = 12.1 Hz, C_4_-H_B_ pyrazoline), 5.65 (1H, dd, *J_BX_* = 11.9 Hz, *J_AX_* = 7.4 Hz, C_5_-H_X_ pyrazoline), 7.06 (1H, s), 7.27 (2H, d, *J* = 7.9 Hz, aromatic), 7.43 (2H, d, *J* = 9.1 Hz, aromatic), 7.67 (2H, d, *J* = 7.9 Hz, aromatic), 7.77 (2H, d, *J* = 9.7 Hz, aromatic), 8.16 (2H, d, *J* = 9.2 Hz, aromatic), 8.37 (2H, d, *J* = 10.4 Hz, aromatic), 8.89 (2H, s, aromatic). ^13^C NMR (125 MHz, *CDCl*_3_) *δ* (ppm): 21.5 (CH_3_), 42.8 (CH_2_, pyrazoline C_4_), 60.6 (CH, pyrazoline C_5_), 108.3 (CH, thiazole C_5_), 124.1 (2CH, aromatic), 126.3 (2CH, aromatic), 126.6 (2CH, aromatic), 127.8 (C, aromatic), 129.0 (2CH, aromatic), 129.6 (2CH, aromatic), 129.7 (2CH, aromatic), 132.2 (C, aromatic), 135.6 (C, aromatic), 137.3 (C, aromatic), 140.5 (C, aromatic), 141.0 (C, aromatic), 146.8 (C, aromatic), 149.4 (C, thiazole C_4_), 152.6 (C, aromatic), 156.2 (2CH, aromatic), 163.4 (C, pyrazoline C_3_), 165.4 (C, thiazole C_2_). HRMS (FAB) calcd. for C_29_H_22_O_2_N_6_ClS [M+H]+: *m*/*z* = 553.1213; found: 553.1185. (Spectral Data: Supplementary Information. Figures S16–S18).

1-(4-(4-Fluorophenyl)thiazol-2-yl)-3-(4-methylphenyl)-5-(2-(4-chlorophenyl)pyrimidin-5-yl)-2-pyrazoline (**BP-3**): ^1^H NMR (500 MHz, *CDCl*_3_) *δ* (ppm): 2.40 (3H, s, CH_3_), 3.36 (1H, dd, *J_AB_* = 17.4 Hz, *J_AX_* = 7.6 Hz, C_4_-H_A_ pyrazoline), 3.96 (1H, dd, *J_BA_* = 17.4 Hz, *J_BX_* = 11.9 Hz, C_4_-H_B_ pyrazoline), 5.64 (1H, dd, *J_BX_* = 12.3 Hz, *J_AX_* = 7.3 Hz, C_5_-H_X_ pyrazoline), 6.77 (1H, s, aromatic), 6.99 (2H, d, *J* = 8.7 Hz, aromatic), 7.25 (2H, d, *J* = 7.3 Hz, aromatic), 7.43 (2H, d, *J* = 8.0 Hz, aromatic), 7.61 (2H, t, *J* = 6.9 Hz, aromatic), 7.66 (2H, d, *J* = 7.6 Hz, aromatic), 8.37 (2H, d, *J* = 8.3 Hz, aromatic), 8.88 (2H, s, aromatic). ^13^C NMR (125 MHz, *CDCl*_3_) *δ* (ppm): 21.7 (CH_3_), 42.4 (CH_2_, pyrazoline C_4_), 60.7 (CH, pyrazoline C_5_), 103.9 (CH, thiazole C_5_), 115.4 (2CH, d, *J* = 125.7 Hz, aromatic), 126.4 (2CH, aromatic), 127.1 (C, aromatic), 127.5 (2CH, d, *J* = 38.0 Hz, aromatic), 128.0 (2CH, aromatic), 128.9 (2CH, aromatic), 129.6 (2CH, d, *J* = 44.3 Hz, aromatic), 132.4 (C, aromatic), 133.5 (C, aromatic), 135.8 (C, aromatic), 137.1 (C, aromatic), 141.7 (C, aromatic), 150.5 (C, thiazole C_4_), 151.9 (C, pyrazoline C_3_), 156.2 (2CH, aromatic), 161.4 (C, aromatic), 163.3 (C, aromatic), 165.2 (C, thiazole C_2_). HRMS (FAB) calcd. for C_29_H_22_N_5_ClFS [M+H]+: *m*/*z* = 526.1268; found: 526.1259. (Spectral Data: Supplementary Information. Figures S19–S21).

1-(4-(4-Chlorophenyl)thiazol-2-yl)-3-(4-methylphenyl)-5-(2-(4-chlorophenyl)pyrimidin-5-yl)-2-pyrazoline (**BP-4**): ^1^H NMR (500 MHz, *CDCl*_3_) *δ* (ppm): 2.4 (3H, s, CH_3_), 3.35 (1H, dd, *J_AB_* = 17.4 Hz, *J_AX_* = 7.0 Hz, C_4_-H_A_ pyrazoline), 3.94 (1H, dd, *J_BA_* = 17.1 Hz, *J_BX_ =* 11.8, Hz, C_4_-H_B_ pyrazoline), 5.61 (1H, dd, *J_BX_* = 12.1 Hz, *J_AX_* = 7.4 Hz, C_5_-H_X_ pyrazoline), 6.81 (1H, s, aromatic), 7.23–7.27 (4H, m, aromatic), 7.42 (2H, d, *J* = 8.4 Hz, aromatic), 7.56 (2H, d, *J* = 9.1 Hz, aromatic), 7.65 (2H, d, *J* = 8.7 Hz, aromatic), 8.36 (2H, d, *J* = 9.4 Hz, aromatic), 8.86 (2H, s, aromatic). ^13^C NMR (125 MHz, *CDCl*_3_) *δ* (ppm): 21.9 (CH_3_), 42.6 (CH_2_, pyrazoline C_4_), 60.9 (CH, pyrazoline C_5_), 104.1 (CH, thiazole C_5_), 126.5 (2CH, aromatic), 127.1 (2CH, aromatic), 128.0 (C, aromatic), 128.7 (2CH, aromatic), 128.8 (2CH, aromatic), 129.5 (2CH, aromatic), 129.6 (2CH, aromatic), 132.4 (C, aromatic), 133.1 (C, aromatic), 133.3 (C, aromatic), 135.8 (C, aromatic), 137.1 (C, aromatic), 140.8 (C, aromatic), 150.4 (C, thiazole C_4_), 152.1 (C, pyrazoline C_3_), 156.3 (2CH, aromatic), 163.3 (C, aromatic), 165.2 (C, thiazole C_2_). HRMS (FAB) calcd. for C_29_H_22_N_5_Cl_2_S [M+H]+: *m*/*z* = 542.0973; found: 542.0545. (Spectral Data: Supplementary Information. Figures S22–S24).

1-(4-(4-Bromophenyl)thiazol-2-yl)-3-(4-methylphenyl)-5-(2-(4-chlorophenyl)pyrimidin-5-yl)-2-pyrazoline (**BP-5**): ^1^H NMR (500 MHz, *CDCl*_3_) *δ* (ppm): 2.4 (3H, s, CH_3_), 3.35 (1H, dd, *J_AB_* = 17.7 Hz, *J_AX_* = 7.8 Hz, C_4_-H_A_ pyrazoline), 3.94 (1H, dd, *J_BA_* = 17.3 Hz, *J_BX_ =* 12.5, Hz, C_4_-H_B_ pyrazoline), 5.61 (1H, dd, *J_BX_* = 11.5 Hz, *J_AX_* = 8.2 Hz, C_5_-H_X_ pyrazoline), 6.83 (1H, s, aromatic), 7.24 (2H, d, *J* = 8.4 Hz, aromatic), 7.41–7.43 (4H, m, aromatic), 7.50 (2H, d, *J* = 7.2 Hz, aromatic), 7.65 (2H, d, *J* = 7.0 Hz, aromatic), 8.36 (2H, d, *J* = 9.2 Hz, aromatic), 8.36 (2H, s, aromatic). ^13^C NMR (125 MHz, *CDCl*_3_) *δ* (ppm): 21.5 (CH_3_), 42.4 (CH_2_, pyrazoline C_4_), 60.5 (CH, pyrazoline C_5_), 104.5 (CH, thiazole C_5_), 121.5 (C, aromatic), 126.5 (2CH, aromatic), 127.4 (2CH, aromatic), 127.9 (C, aromatic), 128.8 (2CH, aromatic), 129.5 (2CH, aromatic), 129.6 (2CH, aromatic), 131.7 (2CH, aromatic), 132.4 (C, aromatic), 133.5 (C, aromatic), 135.8 (C, aromatic), 137.2 (C, aromatic), 140.7 (C, aromatic), 150.4 (C, thiazole C_4_), 152.1 (C, pyrazoline C_3_), 156.3 (2CH, aromatic), 163.2 (C, aromatic), 165.1 (C, thiazole C_2_). HRMS (FAB) calcd. for C_29_H_22_N_5_ClBrS [M+H]+: *m*/*z* = 586.0458; found: 586.0435. (Spectral Data: Supplementary Information. Figures S25–S27).

1-(4-(4-Cyanophenyl)thiazol-2-yl)-3-(4-methylphenyl)-5-(2-(4-chlorophenyl)pyrimidin-5-yl)-2-pyrazoline (**BP-6**): ^1^H NMR (500 MHz, *CDCl*_3_) *δ* (ppm): 2.4 (3H, s, CH_3_), 3.37 (1H, dd, *J_AB_* = 17.5 Hz, *J_AX_* = 7.2 Hz, C_4_-H_A_ pyrazoline), 3.96 (1H, dd, *J_BA_* = 17.6 Hz, *J_BX_ =* 12.0, Hz, C_4_-H_B_ pyrazoline), 5.61 (1H, dd, *J_BX_* = 12.0 Hz, *J_AX_* = 7.3 Hz, C_5_-H_X_ pyrazoline), 6.98 (1H, s, aromatic), 7.25 (2H, d, *J* = 8.2 Hz, aromatic), 7.42 (2H, d, *J* = 8.6 Hz, aromatic), 7.57 (2H, d, *J* = 8.6 Hz, aromatic), 7.66 (2H, d, *J* = 8.4 Hz, aromatic), 7.71 (2H, d, *J* = 8.6 Hz, aromatic), 8.36 (2H, d, *J* = 8.8 Hz, aromatic), 8.86 (2H, s, aromatic). ^13^C NMR (125 MHz, *CDCl*_3_) *δ* (ppm): 21.7 (CH_3_), 42.8 (CH_2_, pyrazoline C_4_), 60.7 (CH, pyrazoline C_5_), 107.4 (CH, thiazole C_5_), 110.7 (C, aromatic), 119.3 (C, C≡N), 126.2 (2CH, aromatic), 126.5 (2CH, aromatic), 127.8 (C, aromatic), 129.6 (2CH, aromatic), 129.6 (2CH, aromatic), 129.7 (2CH, aromatic), 132.2 (C, aromatic), 132.5 (2CH, aromatic), 135.6 (C, aromatic), 137.2 (C, aromatic), 138.7 (C, aromatic), 140.9 (C, aromatic), 149.6 (C, thiazole C_4_), 152.5 (C, pyrazoline C_3_), 156.2 (2CH, aromatic), 163.3 (C, aromatic), 165.3 (C, thiazole C_2_). HRMS (FAB) calcd. for C_30_H_22_N_6_ClS [M+H]+: *m*/*z* = 533.1315; found: 533.1304. (Spectral Data: Supplementary Information. Figures S28–S30).

1-(4-(4-Trifluoromethylphenyl)thiazol-2-yl)-3-(4-methylphenyl)-5-(2-(4-chlorophenyl)pyrimidin-5-yl)-2-pyrazoline (**BP-7**): ^1^H NMR (500 MHz, *CDCl*_3_) *δ* (ppm): 2.40 (3H, s, CH_3_), 3.96 (1H, dd, *J_AB_* = 17.5 Hz, *J_AX_* = 7.7 Hz, C_4_-H_A_ pyrazoline), 3.96 (1H, dd, *J_BA_* = 17.3 Hz, *J_BX_ =* 12.1, Hz, C_4_-H_B_ pyrazoline), 5.64 (1H, dd, *J_BX_* = 12.1 Hz, *J_AX_* = 7.0 Hz, C_5_-H_X_ pyrazoline), 6.94 (1H, s, aromatic), 7.25 (2H, d, *J* = 8.0 Hz, aromatic), 7.43 (2H, d, *J* = 9.1 Hz, aromatic), 7.56 (2H, d, *J* = 8.8 Hz, aromatic), 7.66 (2H, d, *J* = 7.3 Hz, aromatic), 7.74 (2H, d, *J* = 8.0 Hz, aromatic), 8.37 (2H, d, *J* = 8.8 Hz, aromatic), 8.88 (2H, s, aromatic). ^13^C NMR (125 MHz, *CDCl*_3_) *δ* (ppm): 21.7 (CH_3_), 43.1 (CH_2_, pyrazoline C_4_), 60.5 (CH, pyrazoline C_5_), 105.9 (CH, thiazole C_5_), 125.6 (C, CF_3_), 126.0 (2CH, aromatic), 126.6 (2CH, aromatic), 127.9 (C, aromatic), 128.9 (2CH, aromatic), 129.5 (3CH, aromatic), 129.6 (3CH, aromatic), 132.3 (C, aromatic), 135.7 (C, aromatic), 137.1 (C, aromatic), 137.6 (C, aromatic), 137.8 (C, aromatic), 140.8 (C, aromatic), 150.1 (C, thiazole C_4_), 152.2 (C, pyrazoline C_3_), 156.2 (2CH, aromatic), 163.3 (C, aromatic), 165.3 (C, thiazole C_2_). HRMS (FAB) calcd. for C_30_H_22_N_5_ClF_3_S [M+H]+: *m*/*z* = 576.1237; found: 576.1225. (Spectral Data: Supplementary Information. Figures S31–S33).

1-(4-Phenylthiazol-2-yl)-3-(4-methoxyphenyl)-5-(2-(4-chlorophenyl)pyrimidin-5-yl)-2-pyrazoline (**BP-8**): ^1^H NMR (500 MHz, *CDCl*_3_) *δ* (ppm): 3.31 (1H, dd, *J_AB_* = 17.5 Hz, *J_AX_* = 7.2 Hz, C_4_-H_A_ pyrazoline), 3.84 (3H, s, OCH_3_), 3.90 (1H, dd, *J_BA_* = 17.5 Hz, *J_BX_* = 12.1 Hz, C_4_-H_B_ pyrazoline), 5.60 (1H, dd, *J_BX_* = 12.2 Hz, *J_AX_* = 7.4 Hz, C_5_-H_X_ pyrazoline), 6.83 (1H, s, aromatic), 6.94 (2H, t, *J* = 9.4 Hz, aromatic), 7.20–7.24 (1H, m, aromatic), 7.30 (2H, d, *J* = 7.2 Hz, aromatic), 7.41 (2H, d, *J* = 8.4 Hz, aromatic), 7.65 (2H, d, *J* = 6.9 Hz, aromatic), 7.69 (2H, d, *J* = 8.5 Hz, aromatic), 8.36 (2H, d, *J* = 9.9 Hz, aromatic), 8.86 (2H, s, aromatic). ^13^C NMR (125 MHz, *CDCl*_3_) *δ* (ppm): 42.9 (CH_2_, pyrazoline C_4_), 55.4 (OCH_3_), 60.4 (CH, pyrazoline C_5_), 104.1 (CH, thiazole C_5_), 114.1 (2CH, aromatic), 123.7 (C, aromatic), 125.9 (2CH, aromatic), 127.6 (CH, aromatic), 128.1 (2CH, aromatic), 128.5 (2CH, aromatic), 128.8 (2CH, aromatic), 129.6 (2CH, aromatic), 132.5 (C, aromatic), 134.7 (C, aromatic), 135.9 (C, aromatic), 137.0 (C, aromatic), 151.5 (C, thiazole C_4_), 151.8 (C, pyrazoline C_3_), 156.3 (2CH, aromatic), 161.3 (C, aromatic), 163.2 (C, aromatic), 165.4 (C, thiazole C_2_). HRMS (FAB) calcd. for C_29_H_23_ON_5_ClS [M+H]+: *m*/*z* = 524.1312; found: 524.1298. (Spectral Data: Supplementary Information. Figures S34–S36).

1-(4-(4-Nitrophenyl)thiazol-2-yl)-3-(4-methoxyphenyl)-5-(2-(4-chlorophenyl)pyrimidin-5-yl)-2-pyrazoline (**BP-9**): ^1^H NMR (500 MHz, *CDCl*_3_) *δ* (ppm): 3.40 (1H, dd, *J_AB_* = 17.1 Hz, *J_AX_* = 6.8 Hz, C_4_-H_A_ pyrazoline), 3.87 (3H, s, OCH_3_), 4.00 (1H, dd, *J_BA_* = 17.1 Hz, *J_BX_* = 11.5 Hz, C_4_-H_B_ pyrazoline), 5.65 (1H, dd, *J_BX_* = 11.7 Hz, *J_AX_* = 7.3 Hz, C_5_-H_X_ pyrazoline), 6.98 (2H, d, *J* = 10.1 Hz, aromatic), 7.06 (1H, s), 7.44 (2H, d, *J* = 9.1 Hz, aromatic), 7.73 (2H, d, *J* = 8.7 Hz, aromatic), 7.78 (2H, d, *J* = 10.9 Hz, aromatic), 8.17 (2H, d, *J* = 9.7 Hz, aromatic), 8.38 (2H, d, *J* = 9.7 Hz, aromatic), 8.89 (2H, s, aromatic). ^13^C NMR (125 MHz, *CDCl*_3_) *δ* (ppm): 42.7 (CH_2_, pyrazoline C_4_), 55.6 (OCH_3_), 60.5 (CH, pyrazoline C_5_), 107.8 (CH, thiazole C_5_), 114.4 (2CH, aromatic), 123.2 (2CH, aromatic), 124.1 (2CH, aromatic), 126.2 (C, aromatic), 128.2 (2CH, aromatic), 128.9 (2CH, aromatic), 129.6 (2CH, aromatic), 135.6 (C, aromatic), 135.6 (C, aromatic), 137.2 (C, aromatic), 140.5 (C, aromatic), 146.8 (C, aromatic), 149.3 (C, thiazole C_4_), 152.4 (C, aromatic), 156.2 (2CH, aromatic), 161.5 (C, pyrazoline C_3_), 163.4 (C, aromatic), 165.5 (C, thiazole C_2_). HRMS (FAB) calcd. for C_29_H_22_O_3_N_6_ClS [M+H]+: *m*/*z* = 569.1163; found: 569.1144. (Spectral Data: Supplementary Information. Figures S37–S39).

1-(4-(4-Fluorophenyl)thiazol-2-yl)-3-(4-methoxyphenyl)-5-(2-(4-chlorophenyl)pyrimidin-5-yl)-2-pyrazoline (**BP-10**): ^1^H NMR (500 MHz, *CDCl*_3_) *δ* (ppm): 3.32 (1H, dd, *J_AB_* = 17.3 Hz, *J_AX_* = 7.1 Hz, C_4_-H_A_ pyrazoline), 3.84 (3H, s, OCH_3_), 3.91 (1H, dd, *J_BA_* = 16.9 Hz, *J_BX_* = 11.8 Hz, C_4_-H_B_ pyrazoline), 5.60 (1H, dd, *J_BX_* = 12.0 Hz, *J_AX_* = 7.3 Hz, C_5_-H_X_ pyrazoline), 6.74 (1H, s, aromatic), 6.93–6.99 (4H, m), 7.42 (2H, d, *J* = 8.5 Hz, aromatic), 7.59–7.62 (2H, m, aromatic), 7.69 (2H, d, *J* = 8.7 Hz, aromatic), 8.36 (2H, d, *J* = 8.7 Hz, aromatic), 8.90 (2H, s, aromatic). ^13^C NMR (125 MHz, *CDCl*_3_) *δ* (ppm): 42.7 (CH_2_, pyrazoline C_4_), 55.6 (OCH_3_), 60.6 (CH, pyrazoline C_5_), 103.5 (CH, thiazole C_5_), 114.3 (2CH, aromatic), 115.4 (2CH, d, *J* = 98.5 Hz, aromatic), 123.5 (C, aromatic), 127.5 (2CH, d, *J* = 35.4 Hz, aromatic), 128.1 (2CH, aromatic), 128.8 (2CH, aromatic), 129.6 (2CH, aromatic), 130.9 (C, aromatic), 132.4 (C, aromatic), 135.8 (C, aromatic), 137.0 (C, aromatic), 150.5 (C, thiazole C_4_), 151.8 (C, pyrazoline C_3_), 156.3 (2CH, aromatic), 161.4 (C, aromatic), 163.2 (C, aromatic), 163.4 (C, aromatic), 165.3 (C, thiazole C_2_). HRMS (FAB) calcd. for C_29_H_21_ON_5_ClFS [M+H]+: *m*/*z* = 541.1139; found: 541.1155. (Spectral Data: Supplementary Information. Figures S40–S42).

1-(4-(4-Chlorophenyl)thiazol-2-yl)-3-(4-methylphenyl)-5-(2-(4-chlorophenyl)pyrimidin-5-yl)-2-pyrazoline (**BP-11**): ^1^H NMR (500 MHz, *CDCl*_3_) *δ* (ppm): 3.33 (1H, dd, *J_AB_* = 17.2 Hz, *J_AX_* = 7.3 Hz, C_4_-H_A_ pyrazoline), 3.9 (3H, s, OCH_3_), 3.92 (1H, dd, *J_BA_* = 17.4 Hz, *J_BX_ =* 12.0, Hz, C_4_-H_B_ pyrazoline), 5.60 (1H, dd, *J_BX_* = 11.9 Hz, *J_AX_* = 7.4 Hz, C_5_-H_X_ pyrazoline), 6.80 (1H, s, aromatic), 6.95 (2H, d, *J* = 8.6 Hz, aromatic), 7.26 (2H, d, *J* = 8.6 Hz, aromatic), 7.43 (2H, d, *J* = 8.9 Hz, aromatic), 7.57 (2H, d, *J* = 8.3 Hz, aromatic), 7.70 (2H, d, *J* = 9.1 Hz, aromatic), 8.37 (2H, d, *J* = 9.6 Hz, aromatic), 8.86 (2H, s, aromatic). ^13^C NMR (125 MHz, *CDCl*_3_) *δ* (ppm): 42.8 (CH_2_, pyrazoline C_4_), 55.0 (OCH_3_), 60.4 (CH, pyrazoline C_5_), 104.5 (CH, thiazole C_5_), 113.9 (2CH, aromatic), 123.4 (C, aromatic),127.1 (2CH, aromatic), 128.1 (2CH, aromatic), 128.7 (2CH, aromatic), 128.9 (2CH, aromatic), 129.6 (2CH, aromatic), 132.4 (C, aromatic), 133.2 (C, aromatic), 133.3 (C, aromatic), 135.7 (C, aromatic), 137.1 (C, aromatic), 150.4 (C, thiazole C_4_), 151.7 (C, pyrazoline C_3_), 156.3 (2CH, aromatic), 161.3 (C, aromatic), 163.2 (C, aromatic), 165.2 (C, thiazole C_2_). HRMS (FAB) calcd. for C_29_H_22_ON_5_Cl_2_S [M+H]+: *m*/*z* = 558.0922; found: 558.0909. (Spectral Data: Supplementary Information. Figures S43–S45).

1-(4-(4-Bromophenyl)thiazol-2-yl)-3-(4-methoxyphenyl)-5-(2-(4-chlorophenyl)pyrimidin-5-yl)-2-pyrazoline (**BP-12**): ^1^H NMR (500 MHz, *CDCl*_3_) *δ* (ppm): 3.30 (1H, dd, *J_AB_* = 17.1 Hz, *J_AX_* = 7.2 Hz, C_4_-H_A_ pyrazoline), 3.83 (3H, s, OCH_3_), 3.88 (1H, dd, *J_BA_* = 17.3 Hz, *J_BX_ =* 12.2, Hz, C_4_-H_B_ pyrazoline), 5.55 (1H, dd, *J_BX_* = 11.6 Hz, *J_AX_* = 7.3 Hz, C_5_-H_X_ pyrazoline), 6.80 (1H, s, aromatic), 6.93 (2H, d, *J* = 8.6 Hz, aromatic), 7.39–7.42 (4H, m, aromatic), 7.49 (2H, d, *J* = 7.3 Hz, aromatic), 7.67 (2H, d, *J* = 7.6 Hz, aromatic), 8.35 (2H, d, *J* = 8.6 Hz, aromatic), 8.84 (2H, s, aromatic). ^13^C NMR (125 MHz, *CDCl*_3_) *δ* (ppm): 42.8 (CH_2_, pyrazoline C_4_), 55.7 (OCH_3_), 60.8 (CH, pyrazoline C_5_), 104.5 (CH, thiazole C_5_), 114.5 (2CH, aromatic), 121.5 (C, aromatic), 123.4 (C, aromatic), 127.5 (2CH, aromatic), 128.2 (2CH, aromatic), 128.8 (2CH, aromatic), 129.7 (2CH, aromatic), 131.7 (2CH, aromatic), 132.5 (C, aromatic), 133.7 (C, aromatic), 135.6 (C, aromatic), 137.1 (C, aromatic), 150.5 (C, thiazole C_4_), 152.0 (C, pyrazoline C_3_), 156.3 (2CH, aromatic), 161.4 (C, aromatic), 163.3 (C, aromatic), 165.3 (C, thiazole C_2_). HRMS (FAB) calcd. for C_29_H_22_ON_5_ClBrS [M+H]+: *m*/*z* = 602.0417; found: 602.0392. (Spectral Data: Supplementary Information. Figures S46–S48).

1-(4-(4-Cyanophenyl)thiazol-2-yl)-3-(4-methoxyphenyl)-5-(2-(4-chlorophenyl)pyrimidin-5-yl)-2-pyrazoline (**BP-13**): ^1^H NMR (500 MHz, *CDCl*_3_) *δ* (ppm): 3.36 (1H, dd, *J_AB_* = 17.4 Hz, *J_AX_* = 7.3 Hz, C_4_-H_A_ pyrazoline), 3.90 (3H, s, OCH_3_), 3.96 (1H, dd, *J_BA_* = 17.4 Hz, *J_BX_ =* 11.95, Hz, C_4_-H_B_ pyrazoline), 5.61 (1H, dd, *J_BX_* = 12.1 Hz, *J_AX_* = 7.4 Hz, C_5_-H_X_ pyrazoline), 6.96 (2H, d, *J* = 8.7 Hz, aromatic), 6.98 (1H, s, aromatic), 7.43 (2H, d, *J* = 9.3 Hz, aromatic), 7.57 (2H, d, *J* = 9.3 Hz, aromatic), 7.70–7.72 (4H, m, aromatic), 8.36 (2H, d, *J* = 8.8 Hz, aromatic), 8.87 (2H, s, aromatic). ^13^C NMR (125 MHz, *CDCl*_3_) *δ* (ppm): 43.2 (CH_2_, pyrazoline C_4_), 55.7 (OCH_3_), 60.6 (CH, pyrazoline C_5_), 107.4 (CH, thiazole C_5_), 110.6 (C, aromatic), 114.4 (2CH, aromatic), 119.2 (C, C≡N), 123.4 (C, aromatic), 126.2 (2CH, aromatic), 128.2 (2CH, aromatic), 129.0 (2CH, aromatic), 129.6 (2CH, aromatic), 132.2 (C, aromatic), 132.6 (2CH, aromatic), 135.7 (C, aromatic), 137.2 (C, aromatic), 138.7 (C, aromatic), 149.7 (C, thiazole C_4_), 152.3 (C, pyrazoline C_3_), 156.3 (2CH, aromatic), 161.5 (C, aromatic), 163.3 (C, aromatic), 165.4 (C, thiazole C_2_). HRMS (FAB) calcd. for C_30_H_22_ON_6_ClS [M+H]+: *m*/*z* = 549.1264; found: 549.1252. (Spectral Data: Supplementary Information. Figures S49–S51).

1-(4-(4-Trifluoromethylphenyl)thiazol-2-yl)-3-(4-methoxyphenyl)-5-(2-(4-chlorophenyl)pyrimidin-5-yl)-2-pyrazoline (**BP-14**): ^1^H NMR (500 MHz, *CDCl*_3_) *δ* (ppm): 3.33 (1H, dd, *J_AB_* = 17.3 Hz, *J_AX_* = 7.2 Hz, C_4_-H_A_ pyrazoline), 3.84 (3H, s, OCH_3_), 3.91 (1H, dd, *J_BA_* = 17.5 Hz, *J_BX_ =* 12.5, Hz, C_4_-H_B_ pyrazoline), 5.59 (1H, dd, *J_BX_* = 11.3 Hz, *J_AX_* = 7.9 Hz, C_5_-H_X_ pyrazoline), 6.92–6.97 (3H, m, aromatic), 7.41 (2H, d, *J* = 8.4 Hz, aromatic), 7.54 (2H, d, *J* = 8.3 Hz, aromatic), 7.69 (2H, d, *J* = 8.3 Hz, aromatic), 7.73 (2H, d, *J* = 8.1 Hz, aromatic), 8.36 (2H, d, *J* = 8.4 Hz, aromatic), 8.86 (2H, s, aromatic). ^13^C NMR (125 MHz, *CDCl*_3_) *δ* (ppm): 42.9 (CH_2_, pyrazoline C_4_), 55.3 (OCH_3_), 60.3 (CH, pyrazoline C_5_), 105.9 (CH, thiazole C_5_), 114.5 (2CH, aromatic), 123.3 (C, aromatic), 125.5 (C, CF_3_), 125.9 (2CH, aromatic), 128.2 (2CH, aromatic), 128.9 (3CH, aromatic), 129.5 (3CH, aromatic), 132.3 (C, aromatic), 133.6 (C, aromatic), 135.7 (C, aromatic), 137.2 (C, aromatic), 137.9 (C, aromatic), 150.2 (C, thiazole C_4_), 152.1 (C, pyrazoline C_3_), 156.3 (2CH, aromatic), 161.4 (C, aromatic), 163.3 (C, aromatic), 165.3 (C, thiazole C_2_). HRMS (FAB) calcd. for C_30_H_22_ON_5_ClF_3_S [M+H]+: *m*/*z* = 592.1186; found: 592.1192. (Spectral Data: Supplementary Information. Figures S52–S54).

### 4.2. Cytotoxicity

A549 and MCF-7 human cancer cell lines were cultured in Dulbecco’s Modified Eagle’s Medium (DMEM) (Wako Pure Chemical Industries, Osaka, Japan) supplemented with 10% fetal bovine serum (FBS) (Equitech-Bio, Kerrville, TX, USA). Jurkat cells, derived from human leukemia, were maintained in RPMI 1640 medium (Wako Pure Chemical Industries) with 10% FBS. PBMCs (Precision Bioservices, Frederick, MD, USA) were cultured in RPMI 1640 medium supplemented with 10% human AB serum (HS) (Gemini, Woodland, CA, USA). All media were supplemented with 89 μg/mL of streptomycin (Meiji Seika Pharma, Tokyo, Japan). Cells were incubated at 37 °C in a humidified atmosphere containing 95% air and 5% CO_2_. Cancer cells and PBMCs were seeded into 24-well and 96-well microtiter tissue culture plates (Iwaki, Asahi Glass Co., Chiba, Japan) at densities of 4 × 10^4^ cells/mL and 5 × 10^5^ cells/mL, respectively, and incubated for 72 h prior to drug treatment. The optimal seeding density for cytotoxicity assays was determined through preliminary testing. Stock solutions of the test compounds and lapatinib (Sigma-Aldrich, St. Louis, MO, USA) were prepared in dimethyl sulfoxide (DMSO; Wako Pure Chemical Industries) at concentrations of 0.1 mM, 0.3 mM, 1 mM, 3 mM, and 10 mM, and subsequently diluted into fresh culture medium, with the final DMSO concentration maintained at 1%. The cellular reduction MTT (Dojindo Molecular Technologies, Kumamoto, Japan) was assessed following established protocols from the literature, with slight modifications [11,28,36].

### 4.3. Apoptosis

A549 and MCF-7 cells were treated with **B-1** and **B-2** at its IC_50_ concentration for 24 h. Following treatment, the cells were detached using 0.05% trypsin, washed twice with 1× binding buffer, and incubated in the dark at r.t. for 20 min with a staining solution containing 50 μL of 1× binding buffer, 4 μL of FITC-Annexin V solution, 4 μL of ethidium homodimer III solution, and 4 μL of Hoechst 33342 solution. After staining, the cells were washed again with 1× binding buffer, fixed in 2% paraformaldehyde, rinsed with PBS, and analyzed using the Biorevo Fluorescence BZ-9000 all-in-one fluorescence microscope (Keyence, Osaka, Japan) [11,28,36].

### 4.4. EGFR Inhibition

The kinase profiling assay (EGFR Kinase Enzyme System, V3831) was conducted according to the manufacturer’s protocol (Promega Corporation, Madison, WI, USA), with minor modifications [11,28,36]. In brief, 95 μL of 2.5× kinase buffer and 15 μL of 100 μM ATP solution were used to prepare dilutions of recombinant human EGFR (amino acids 695 to C-terminus) and its substrate (4:1 Glu:Tyr mixture). Reactions were set up in a 384-well plate, each containing 2 μL of kinase working stock, 2 μL of ATP/substrate working stock, and 1 μL of test compound solution or 5% DMSO as a control. After a 2 h incubation at r.t., kinase activity was measured using the ADP-Glo Kinase Assay (Promega), following the manufacturer’s instructions. Specifically, 5 μL of ADP-Glo Reagent was added to each well and incubated for 40 min, followed by 10 μL of Kinase Detection Reagent and a further 30 min incubation at r.t. Luminescence was recorded using the Infinite M1000 microplate reader (Tecan, Grödig, Austria) with an integration time of 0.5 to 1 s to assess the compounds’ kinase inhibitory effects in a dose-dependent manner.

### 4.5. In Silico Studies

The EGFR crystal structure was retrieved from the RCSB Protein Data Bank under the accession code PDB ID: 1XKK [42]. Docking preparation was carried out using Maestro’s PrepWizard module [43], where Prime was used to add any missing chains, and PropKa calculated protonation states at physiological pH. The receptor–ligand complex was energy-minimized using the Optimized Potentials for Liquid Simulations (OPLS 2005) force field [43]. **B-1, B-2**, and lapatinib were sketched and optimized in the Maestro workspace, before being prepared using the LigPrep module at physiological pH with energy minimization using the same OPLS 2005 force field. Grid generation and molecular docking were performed using Glide in standard precision (SP) mode for **B-1, B-2**, and lapatinib [47,48]. Additionally, QikProp [44], ADMETlab 3.0 [45], and the SwissADME web tool [46] were employed to predict the pharmacokinetic and toxicity properties of **B-1** and **B-2**.

## 5. Conclusions

The development of small-molecule EGFR inhibitors plays a crucial role in advancing treatments for NSCLC and breast cancer. Among newly synthesized compounds, **B-2** and **B-1** demonstrated strong cytotoxic effects against the A549 NSCLC and MCF-7 breast cancer cell lines, respectively, while exhibiting minimal toxicity toward healthy cells (PBMCs). Notably, **B-2** induced significant apoptosis in both A549 and MCF-7 cells, whereas **B-1** was effective primarily in MCF-7 cells. **B-2** exhibited substantial EGFR inhibition at a concentration of 10 µM, a result further corroborated by in silico docking studies targeting the ATP-binding site of EGFR. Additionally, ADMET predictions indicated that both **B-1** and **B-2** possess favourable pharmacokinetic and safety profiles. Overall, **B-2** emerges as a promising small-molecule EGFR inhibitor with potential for further development against NSCLC and breast cancer.

## Figures and Tables

**Table 1:** The IC_50_ values of **B-1, B-2, B-6, BP-1**, and **BP-10** against A549, MCF-7, Jurkat cells as well as PBMCs compared to those of lapatinib.

Compound	IC_50_ Values (μM)				SI *
	A549 Cells	MCF-7 Cells	Jurkat Cells	PBMCs	
**B** **-** **1**	>100	6.10 ± 1.26	2.12 ± 0.48	121.02 ± 14.75	57.08
**B** **-** **2**	2.14 ± 0.83	8.91 ± 1.38	2.96 ± 0.44	68.67 ± 9.82	23.20
**B** **-** **6**	>100	6.52 ± 0.97	15.06 ± 2.85	112.73 ± 15.07	7.49
**BP** **-** **1**	>100	91.58 ± 10.20	>100		
**BP** **-** **10**	>100	76.04 ± 9.91	>100		
Lapatinib	18.21 ± 3.25	9.71 ± 1.12	1.43 ± 0.35	11.04 ± 2.47	7.72

* Selectivity Index (SI) = IC_50_ for PBMC/IC_50_ for Jurkat cell line.

## Data Availability

The original contributions presented in this study are included in the article. Further inquiries can be directed to the corresponding authors.

## Supplementary Materials

The following supporting information can be downloaded at: https://www.mdpi.com/article/10.3390/ijms26157065/s1.
