# Supplementary material for: Design, Synthesis, and Anticancer Evaluation of New Small-Molecule EGFR Inhibitors Targeting NSCLC and Breast Cancer
Source: Int J Mol Sci. 2025 Jul 22;26(15):7065. doi: 10.3390/ijms26157065 (PMC12346557; doi:10.3390/ijms26157065)
Supplement: Supplementary file 1 [file ijms-26-07065-s001.zip › ijms-3730907-supplementary.pdf]

Supplementary Information

Design, Synthesis, and Anticancer Evaluation of New Small-Molecule EGFR Inhibitors Targeting NSCLC and Breast Cancer

Belgin Sever [1,2,*], Masami Otsuka [2,3], Mikako Fujita [2], Halilibrahim Ciftci [2,3,4,5,*]

[1] Department of Pharmaceutical Chemistry, Faculty of Pharmacy, Anadolu University, Eskisehir 26470, Türkiye
[2] Medicinal and Biological Chemistry Science Farm Joint Research Laboratory, Faculty of Life Sciences, Kumamoto University, Kumamoto 862-0973, Japan; motsuka@gpo.kumamoto-u.ac.jp (M.O.); mfujita@kumamoto-u.ac.jp (M.F.)
[3] Department of Drug Discovery, Science Farm Ltd., Kumamoto 862-0976, Japan
[4] Department of Molecular Biology and Genetics, Burdur Mehmet Akif Ersoy University, Istiklal Campus, Burdur 15030, Türkiye
[5] Department of Bioengineering Sciences, Izmir Katip Celebi University, Izmir 35620, Türkiye

* Correspondence: belginsever@anadolu.edu.tr (B.S.); hciftci@mehmetakif.edu.tr (H.C.)

Supplementary Figures

Figure S1: ^{1}H NMR Spectrum of **B-1**
Figure S2: ^{13}C NMR Spectrum of **B-1**
Figure S3: Mass Spectrum of **B-1** (*: the *m/z* intensity value)
Figure S4: ^{1}H NMR Spectrum of **B-2**
Figure S5: ^{13}C NMR Spectrum of **B-2**
Figure S6: Mass Spectrum of **B-2** (*: the *m/z* intensity value)
Figure S7: ^{1}H NMR Spectrum of **B-6**
Figure S8: ^{13}C NMR Spectrum of **B-6**
Figure S9: Mass Spectrum of **B-6** (*: the *m/z* intensity value)
Figure S10: ^{1}H NMR Spectrum of **B-7**
Figure S11: ^{13}C NMR Spectrum of **B-7**
Figure S12: Mass Spectrum of **B-7** (*: the *m/z* intensity value)
Figure S13: ^{1}H NMR Spectrum of **BP-1**
Figure S14: ^{13}C NMR Spectrum of **BP-1**
Figure S15: Mass Spectrum of **BP-1** (*: the *m/z* intensity value)
Figure S16: ^{1}H NMR Spectrum of **BP-2**
Figure S17: ^{13}C NMR Spectrum of **BP-2**
Figure S18: Mass Spectrum of **BP-2** (*: the *m/z* intensity value)
Figure S19: ^{1}H NMR Spectrum of **BP-3**
Figure S20: ^{13}C NMR Spectrum of **BP-3**
Figure S21: Mass Spectrum of **BP-3** (*: the *m/z* intensity value)
Figure S22: ^{1}H NMR Spectrum of **BP-4**
Figure S23: ^{13}C NMR Spectrum of **BP-4**
Figure S24: Mass Spectrum of **BP-4** (*: the *m/z* intensity value)
Figure S25: ^{1}H NMR Spectrum of **BP-5**
Figure S26: ^{13}C NMR Spectrum of **BP-5**
Figure S27: Mass Spectrum of **BP-5** (*: the *m/z* intensity value)
Figure S28: ^{1}H NMR Spectrum of **BP-6**
Figure S29: ^{13}C NMR Spectrum of **BP-6**
Figure S30: Mass Spectrum of **BP-6** (*: the *m/z* intensity value)
Figure S31: ^{1}H NMR Spectrum of **BP-7**
Figure S32: ^{13}C NMR Spectrum of **BP-7**
Figure S33: Mass Spectrum of **BP-7** (*: the *m/z* intensity value)
Figure S34: ^{1}H NMR Spectrum of **BP-8**
Figure S35: ^{13}C NMR Spectrum of **BP-8**
Figure S36: Mass Spectrum of **BP-8** (*: the *m/z* intensity value)
Figure S37: ^{1}H NMR Spectrum of **BP-9**
Figure S38: ^{13}C NMR Spectrum of **BP-9**
Figure S39: Mass Spectrum of **BP-9** (*: the *m/z* intensity value)

Figure S40: ^{1}H NMR Spectrum of **BP-10**
Figure S41: ^{13}C NMR Spectrum of **BP-10**
Figure S42: Mass Spectrum of **BP-10** (*: the *m*/*z* intensity value)
Figure S43: ^{1}H NMR Spectrum of **BP-11**
Figure S44: ^{13}C NMR Spectrum of **BP-11**
Figure S45: Mass Spectrum of **BP-11** (*: the *m*/*z* intensity value)
Figure S46: ^{1}H NMR Spectrum of **BP-12**
Figure S47: ^{13}C NMR Spectrum of **BP-12**
Figure S48: Mass Spectrum of **BP-12** (*: the *m*/*z* intensity value)
Figure S49: ^{1}H NMR Spectrum of **BP-13**
Figure S50: ^{13}C NMR Spectrum of **BP-13**
Figure S51: Mass Spectrum of **BP-13** (*: the *m*/*z* intensity value)
Figure S52: ^{1}H NMR Spectrum of **BP-14**
Figure S53: ^{13}C NMR Spectrum of **BP-14**
Figure S54: Mass Spectrum of **BP-14** (*: the *m*/*z* intensity value)

Figure S1. ^{1}H NMR Spectrum of **B-1**

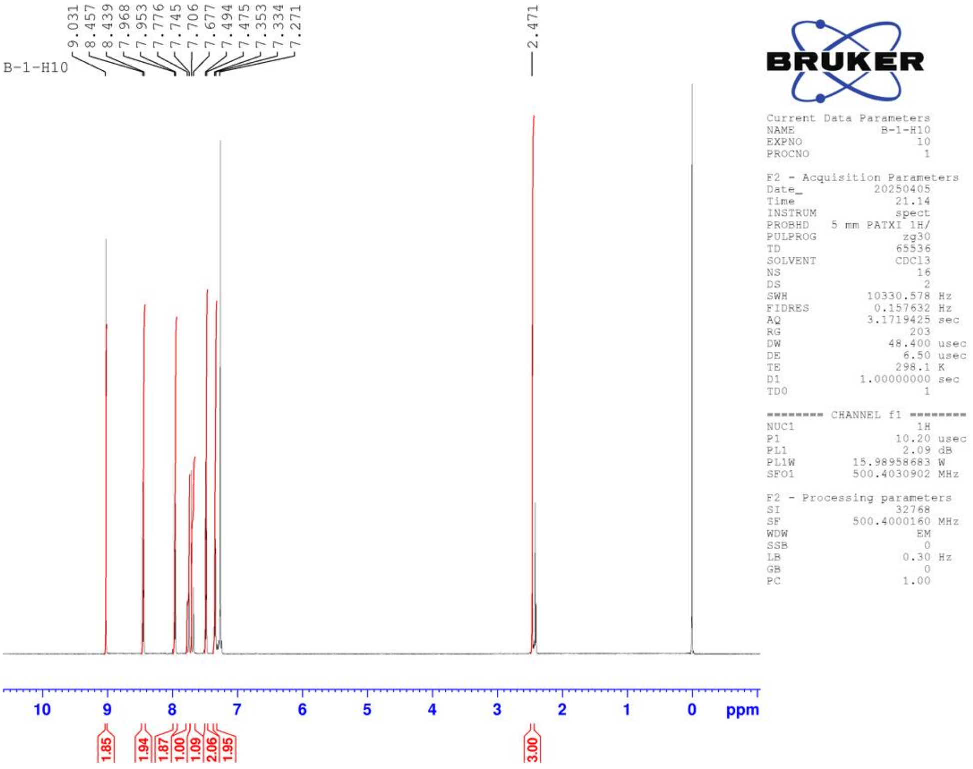

Figure S2. ^{13}C NMR Spectrum of **B-1**

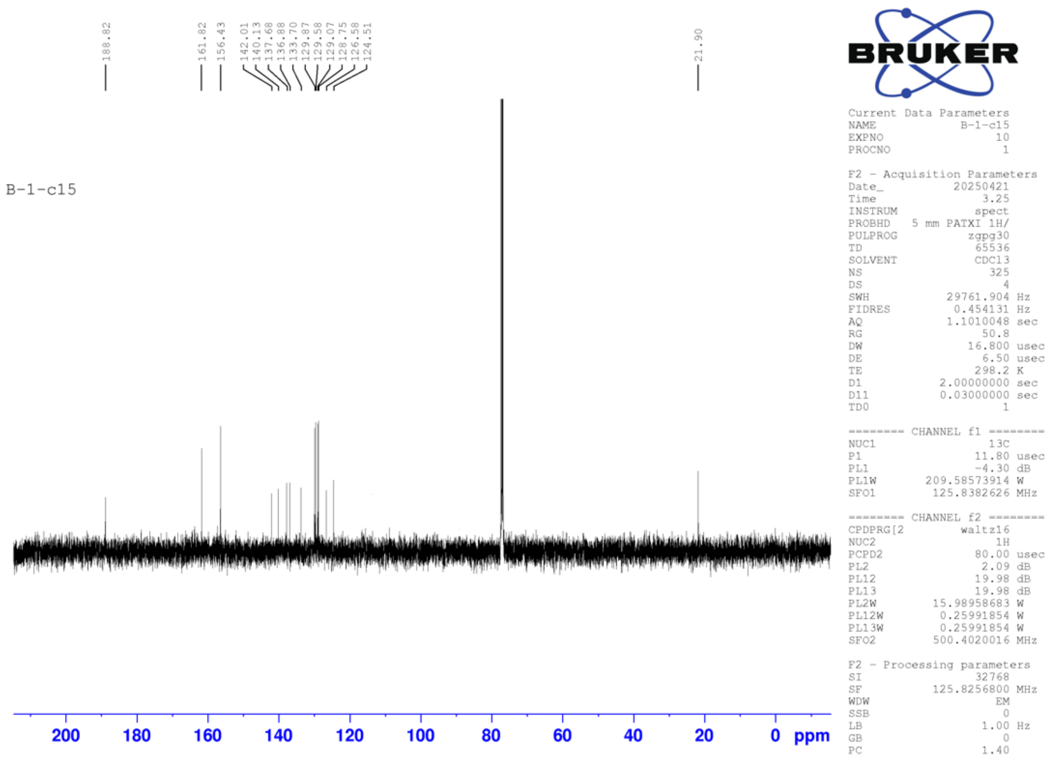

Figure S3: Mass Spectrum of **B-1**

```
[ Elemental Composition ]                                        Page: 1
Data   : gousei993                    Date : 26-Mar-2024 13:38
Sample: B-1/sever belgin
Note : NBA
Inlet : Direct                        Ion Mode : FAB+
RT : 1.00 min                         Scan#: (3,7)
Elements : C 100/0, H 100/0, O 2/0, N 3/1, Cl 2/0
Mass Tolerance       : 20ppm, 10mmu if m/z < 500, 20mmu if m/z > 1000
Unsaturation (U.S.) : -0.5 - 100.0

Observed m/z Int%   Err[ppm / mmu]    U.S.  Composition
  335.0949    18.1     +0.7 /  +0.3   18.0  C 23 H 13 O 2 N
                       -0.7 /  -0.2   13.5  C 20 H 16 O N 2 Cl
                       -2.2 /  -0.7    9.0  C 17 H 19 N 3 Cl 2
```

```
[ Theoretical Ion Distribution ]                                 Page: 1
Molecular Formula : C20 H16 O N2 Cl
                      (m/z   335.0951, MW   335.8128, U.S. 13.5)
Base Peak :   335.0951, Averaged MW :   335.8138(a),   335.8167(w)

    m/z       INT.
  335.0951 100.0000 **********************************************************
  336.0983  23.2575 *************
  337.0929  34.7583 ********************
  338.0956   7.6654 ****
  339.0985   0.9032 *
  340.1014   0.0736
  341.1042   0.0046
  342.1069   0.0002
```

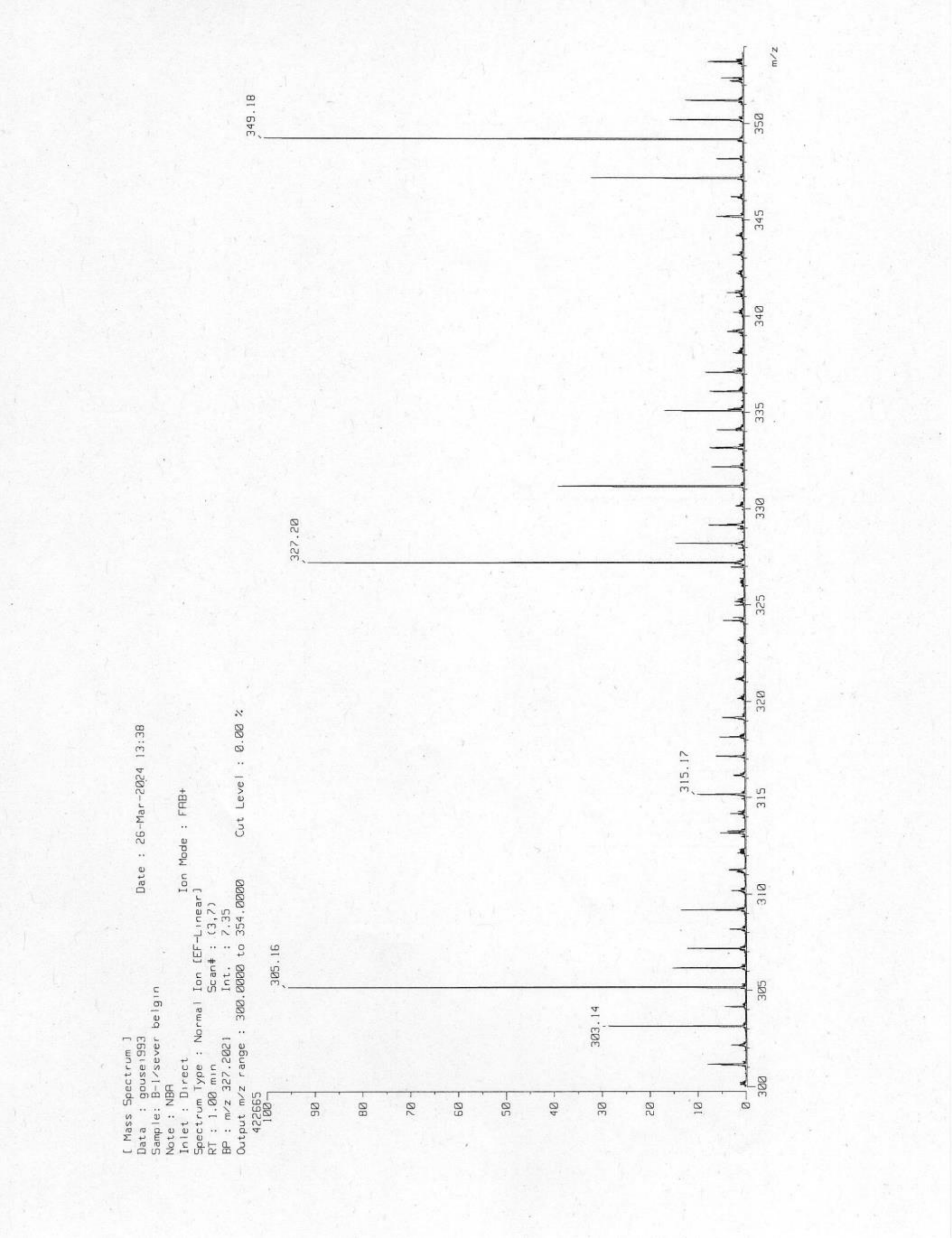
[Mass Spectrum]
Data : gouse1993
Sample: B-1/sever belgin
Note : NBA
Inlet : Direct
Date : 26-Mar-2024 13:38
Ion Mode : FAB+
Spectrum Type : Normal Ion [EF-Linear]
RT : 1.00 min
Scan# : (3,7)
BP : m/z 327.2021
Int. : 7.35
Output m/z range : 300.0000 to 354.0000
Cut Level : 0.00 %
422665
303.14
305.16
315.17
327.20
349.18
m/z

Figure S4: ^{1}H NMR Spectrum of **B-2**

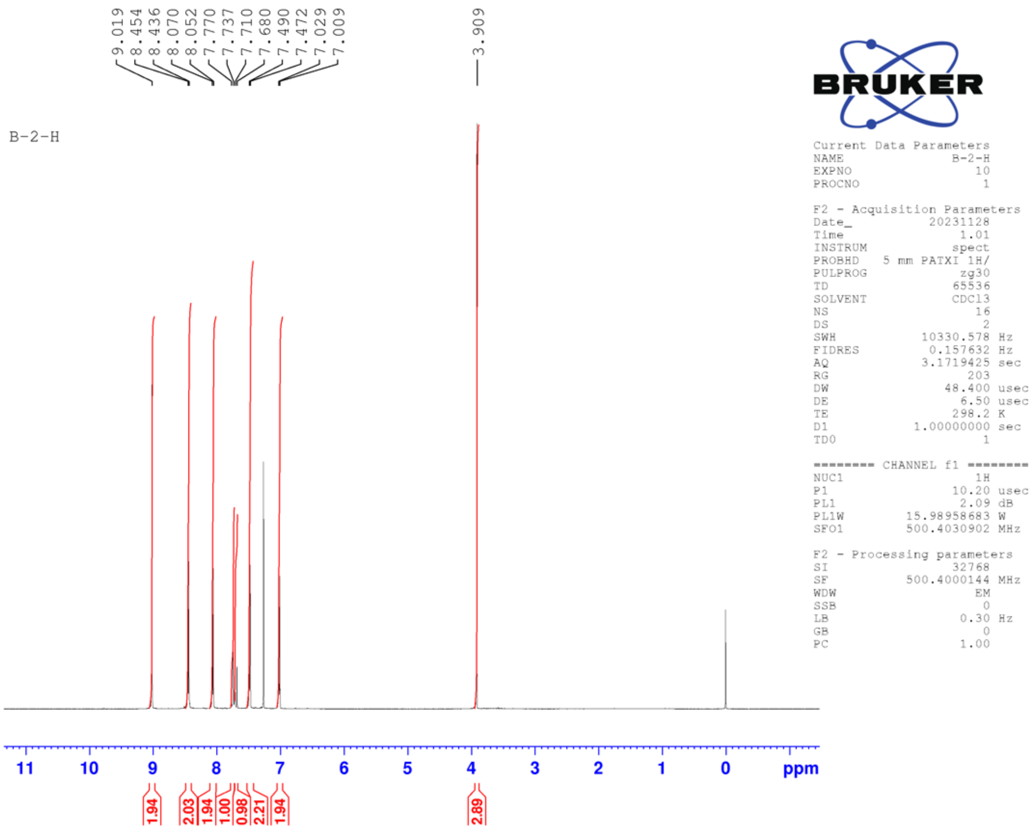

Figure S5: ^{13}C NMR Spectrum of **B-2**

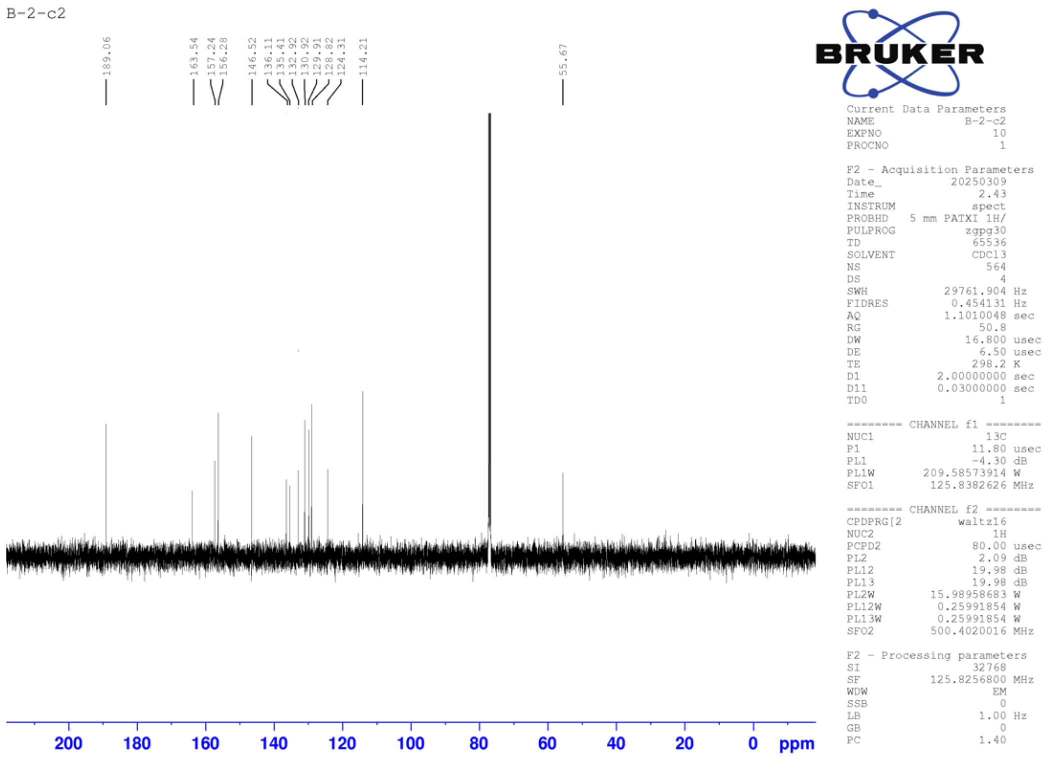

Figure S6: Mass Spectrum of **B-2**

```
[ Elemental Composition ]                                        Page: 1
Data   : gousei983                    Date : 25-Mar-2024 15:29
Sample: B-2/sever belgin
Note : NBA
Inlet : Direct                        Ion Mode : FAB+
RT : 1.00 min                         Scan#: (3,7)
Elements : C 100/0, H 100/0, O 3/1, N 3/1, Cl 2/0
Mass Tolerance       : 20ppm, 10mmu if m/z < 500, 20mmu if m/z > 1000
Unsaturation (U.S.) : -0.5 - 100.0

Observed m/z Int%   Err[ppm / mmu]    U.S.  Composition
  351.0916    38.8      +5.9 /  +2.1   18.0  C 23 H 13 O 3 N
                       -26.1 /  -9.2   18.0  C 22 H 13 O 2 N 3
                        +4.5 /  +1.6   13.5  C 20 H 16 O 2 N 2 Cl
                        +3.1 /  +1.1    9.0  C 17 H 19 O N 3 Cl 2
```

```
[ Theoretical Ion Distribution ]                                 Page: 1
Molecular Formula : C20 H16 O2 N2 Cl
                      (m/z   351.0900, MW   351.8122, U.S. 13.5)
Base Peak :    351.0900, Averaged MW :   351.8131(a),   351.8159(w)

   m/z       INT.
  351.0900 100.0000 **********************************************************
  352.0932  23.2956 **************
  353.0879  34.9676 ********************
  354.0906   7.7253 ****
  355.0933   0.9758 *
  356.0960   0.0893
  357.0987   0.0064
  358.1013   0.0004
```

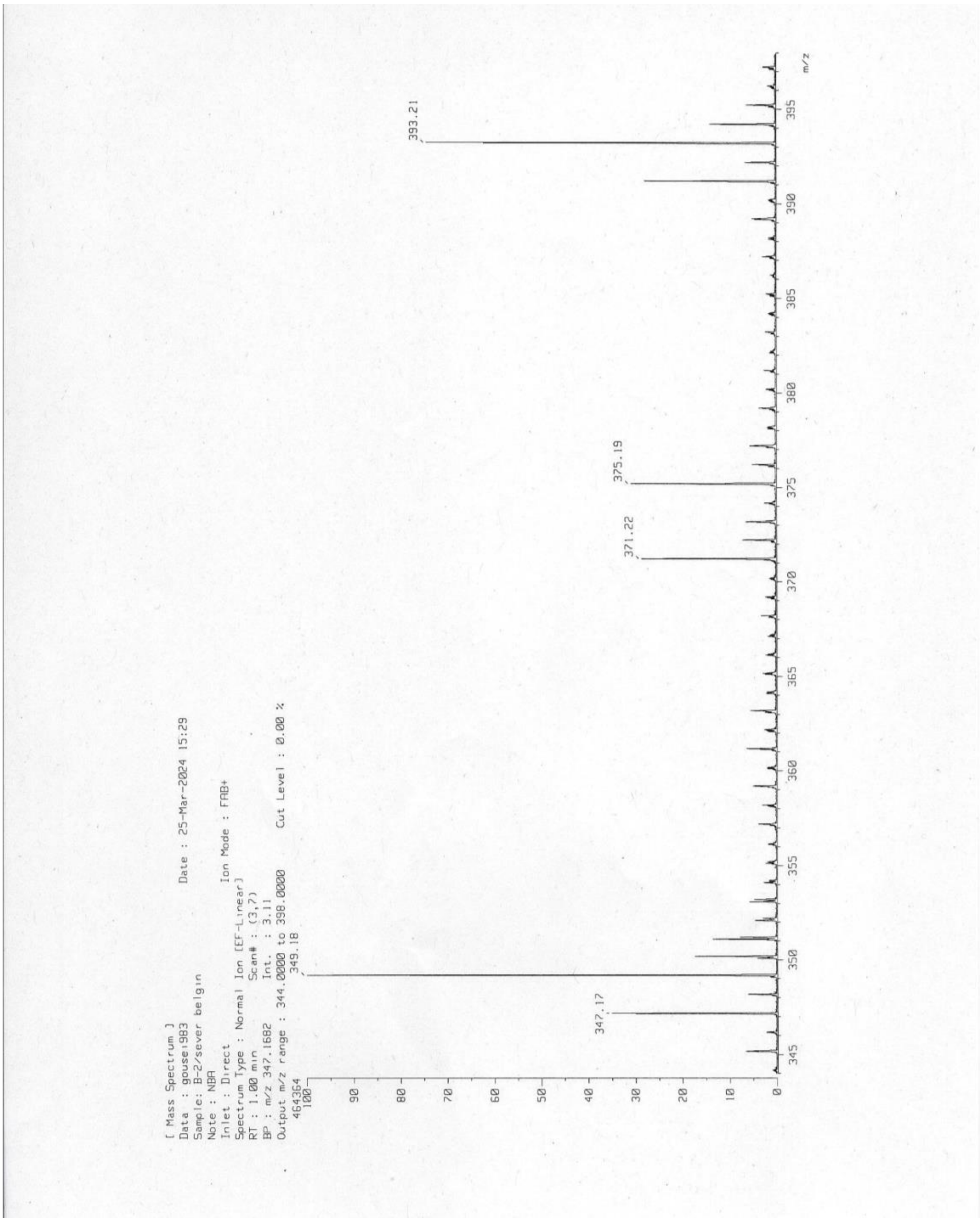
[Mass Spectrum]
Data : gouse1983
Date : 25-Mar-2024 15:29
Sample: B-2/sever belgin
Note : NBA
Inlet : Direct
Ion Mode : FAB+
Spectrum Type : Normal Ion [EF-Linear]
RT : 1.00 min
Scan# : (3,7)
BP : m/z 347.1682
Int. : 3.11
Output m/z range : 344.0000 to 398.0000
Cut Level : 0.00 %
464364
349.18
347.17
371.22
375.19
393.21
m/z

Figure S7: ^{1}H NMR Spectrum of **B-6**

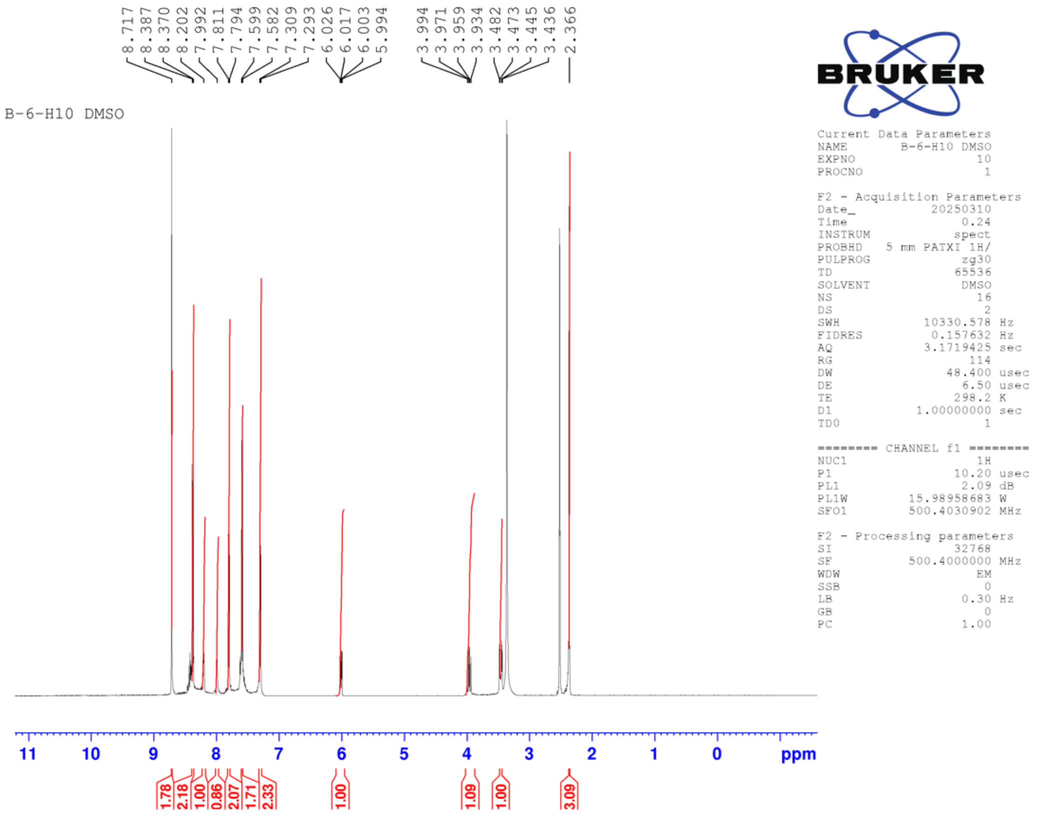

Figure S8: ^{13}C NMR Spectrum of **B-6**

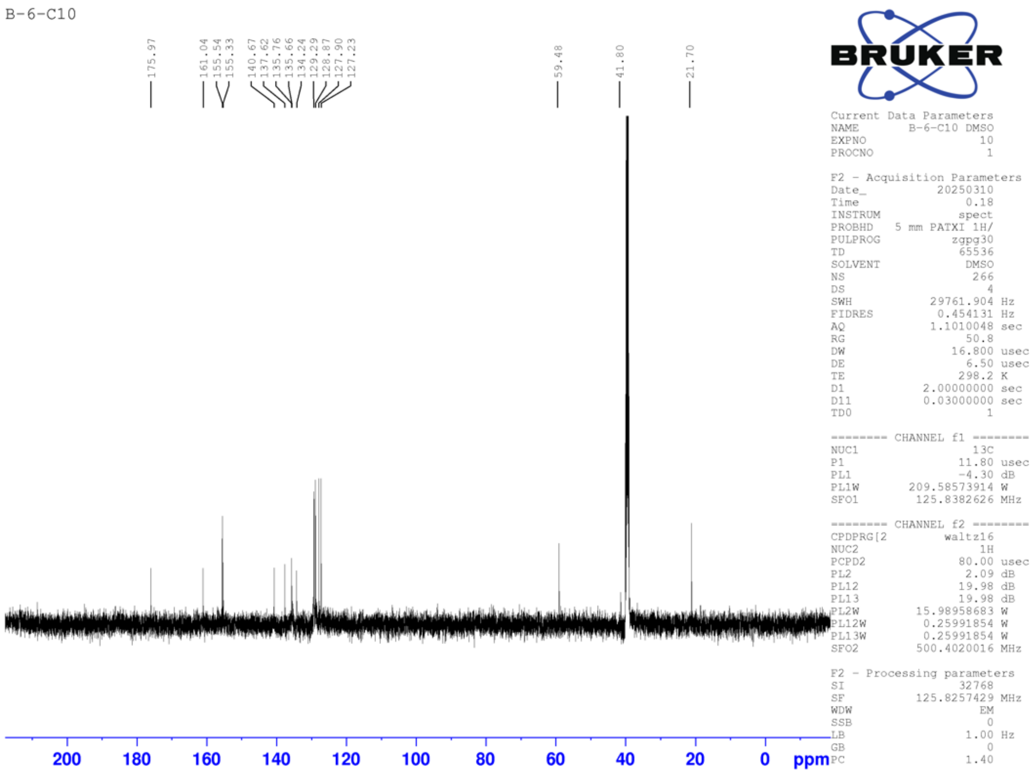

Figure S9: Mass Spectrum of **B-6**

[Elemental Composition] Page: 1

Data : gousei830 Date : 08-Dec-2023 11:04

Sample: B-6/sever belgin

Note : NBA

Inlet : Direct Ion Mode : FAB+

RT : 2.00 min Scan#: (7,11)

Elements : C 100/0, H 100/0, N 6/4, Cl 2/0, S 2/0

Mass Tolerance : 20ppm, 10mmu if m/z < 500, 20mmu if m/z > 1000

Unsaturation (U.S.) : -0.5 - 100.0

Observed m/z	Int%	Err[ppm / mmu]	U.S.	Composition
408.1058	65.5	-16.0 / -6.5	24.0	C 26 H 12 N 6
		-20.4 / -8.3	19.0	C 25 H 17 N 4 Cl
		+10.4 / +4.2	19.5	C 24 H 15 N 5 Cl
		-24.2 / -9.9	20.0	C 23 H 16 N 6 S
		+2.1 / +0.9	15.5	C 21 H 19 N 5 Cl S
		-6.1 / -2.5	11.5	C 18 H 23 N 5 Cl S 2
		+20.2 / +8.2	7.0	C 16 H 26 N 4 Cl 2 S 2

[Theoretical Ion Distribution] Page: 1

Molecular Formula : C21 H19 N5 Cl S

(m/z 408.1050, MW 408.9344, U.S. 15.5)

Base Peak : 408.1050, Averaged MW : 408.9341(a), 408.9369(w)

m/z	INT.	
408.1050	100.0000	**
409.1078	26.2680	***************
410.1026	39.7226	***********************
411.1049	9.7962	******
412.1023	2.6509	**
413.1028	0.4633	
414.1038	0.0571	
415.1049	0.0054	
416.1063	0.0004	

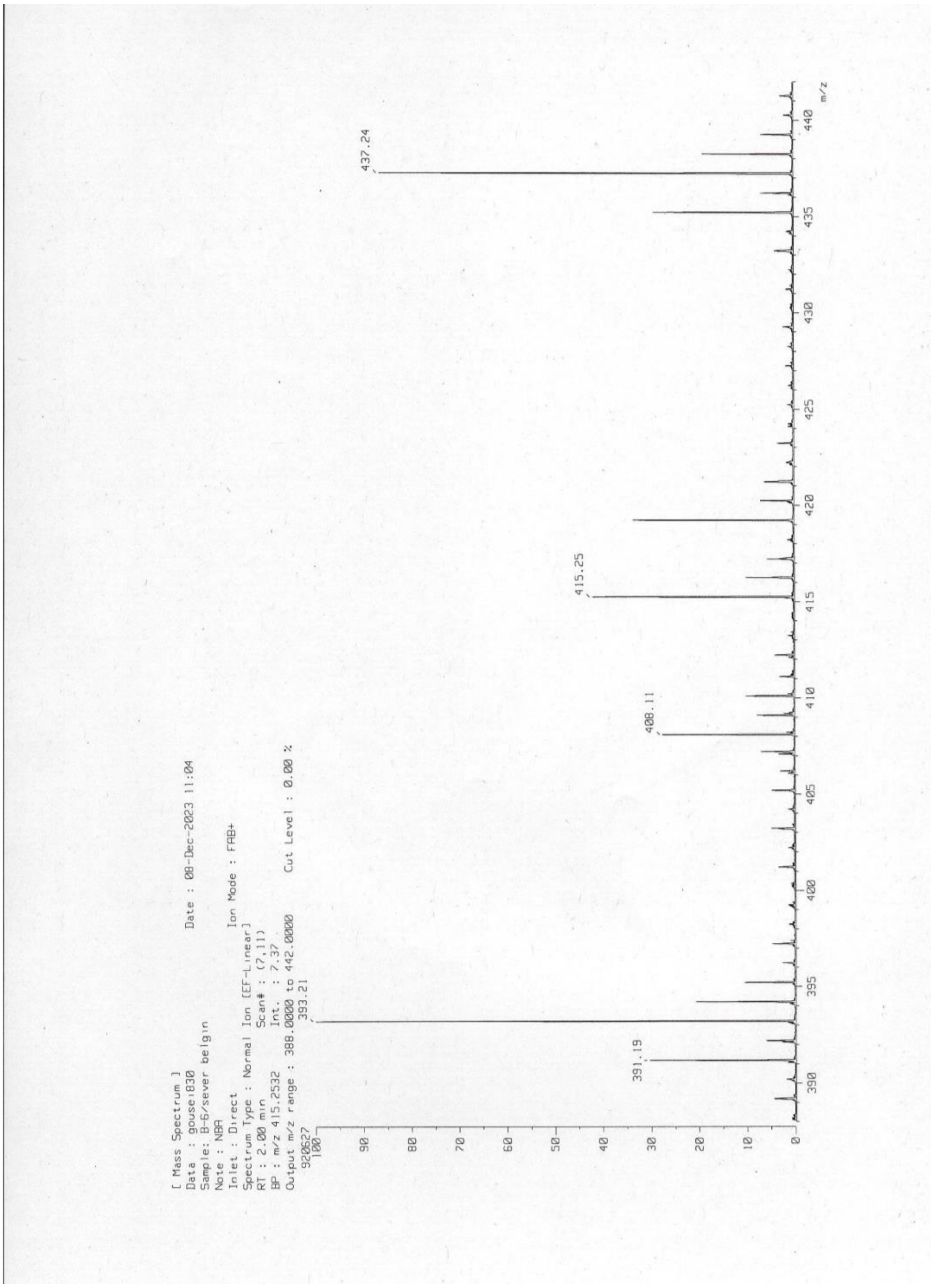
[Mass Spectrum]
Data : gouse1830
Sample: B-6/sever beigin
Note : NBA
Inlet : Direct
Spectrum Type : Normal Ion [EF-Linear]
RT : 2.00 min
BP : m/z 415.2532
Output m/z range : 388.0000 to 442.0000
Date : 08-Dec-2023 11:04
Ion Mode : FAB+
Scan# : (7,11)
Int. : 7.37
Cut Level : 0.00 %
920627
100
90
80
70
60
50
40
30
20
10
0
393.21
391.19
408.11
415.25
437.24
390
395
400
405
410
415
420
425
430
435
440
m/z

Figure S10: ^{1}H NMR Spectrum of **B-7**

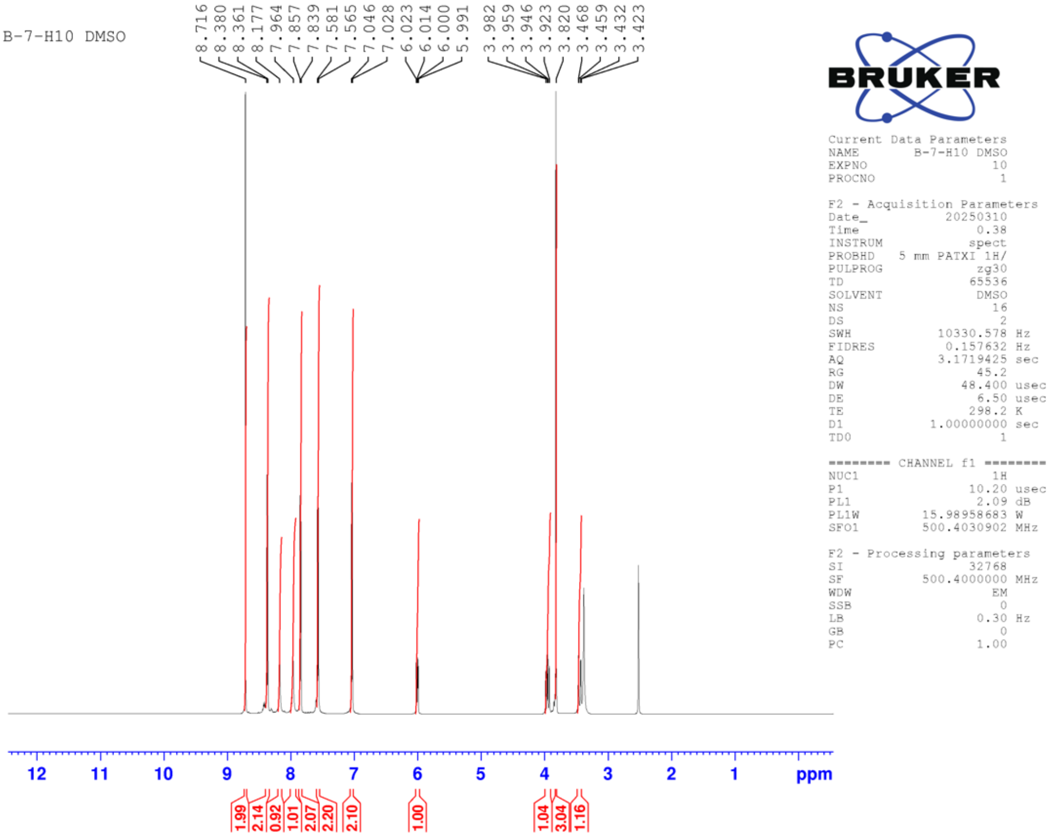

Figure S11: ^{13}C NMR Spectrum of **B-7**

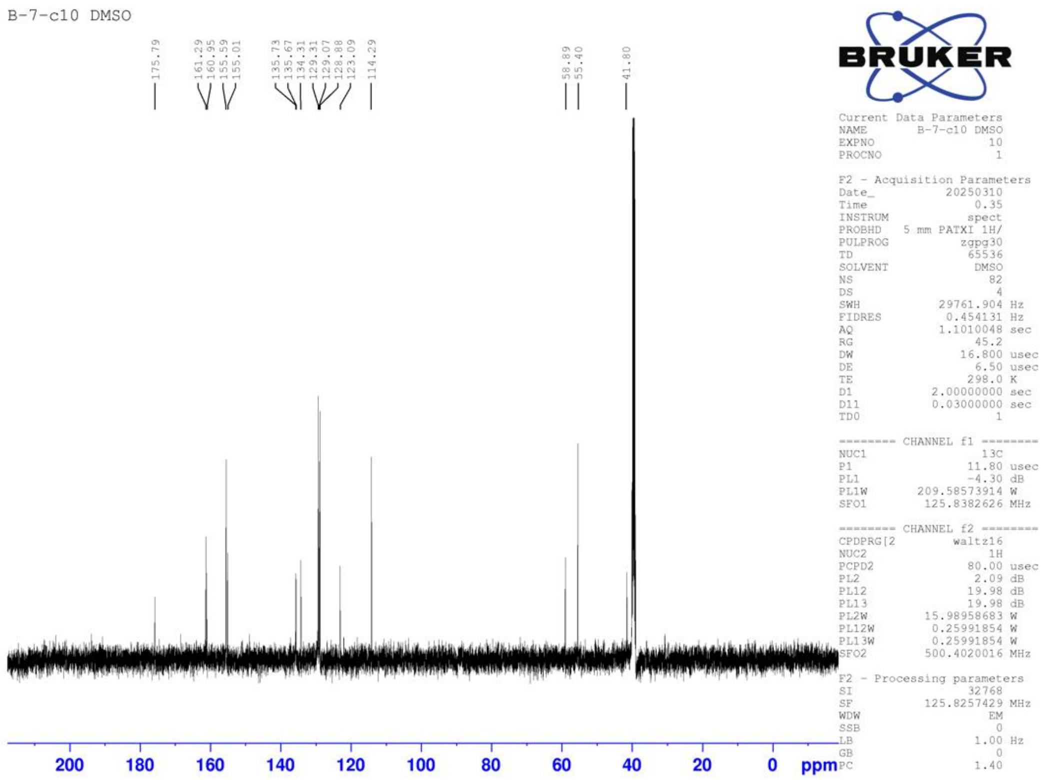

Figure S12: Mass Spectrum of **B-7**

[Elemental Composition] Page: 1
Data : gousei831 Date : 08-Dec-2023 11:16
Sample: B-7/sever belgin
Note : NBA
Inlet : Direct Ion Mode : FAB+
RT : 1.50 min Scan#: (5,9)
Elements : C 100/0, H 100/0, O 2/0, N 6/4, Cl 2/0, S 2/0
Mass Tolerance : 20ppm, 10mmu if m/z < 500, 20mmu if m/z > 1000
Unsaturation (U.S.) : -0.5 - 100.0

Observed m/z	Int%	Err[ppm / mmu]	U.S.	Composition
424.1000	70.0	+9.5 / +4.0	24.0	C 27 H 12 O 2 N 4
		-17.0 / -7.2	24.0	C 26 H 12 O N 6
		-21.3 / -9.1	19.0	C 25 H 17 O N 4 Cl
		+8.3 / +3.5	19.5	C 24 H 15 O N 5 Cl
		-22.5 / -9.5	14.5	C 22 H 20 N 5 Cl 2
		+7.2 / +3.0	15.0	C 21 H 18 N 6 Cl 2
		+1.5 / +0.6	20.0	C 24 H 16 O 2 N 4 S
		+0.4 / +0.2	15.5	C 21 H 19 O N 5 Cl S
		-0.8 / -0.3	11.0	C 18 H 22 N 6 Cl 2 S
		-6.4 / -2.7	16.0	C 21 H 20 O 2 N 4 S 2
		+23.2 / +9.8	16.5	C 20 H 18 O 2 N 5 S 2
		-7.6 / -3.2	11.5	C 18 H 23 O N 5 Cl S 2
		+22.1 / +9.4	12.0	C 17 H 21 O N 6 Cl S 2
		+17.7 / +7.5	7.0	C 16 H 26 O N 4 Cl 2 S 2
		-8.7 / -3.7	7.0	C 15 H 26 N 6 Cl 2 S 2

[Theoretical Ion Distribution] Page: 1
Molecular Formula : C21 H19 O N5 Cl S
(m/z 424.0999, MW 424.9338, U.S. 15.5)
Base Peak : 424.0999, Averaged MW : 424.9334(a), 424.9361(w)

m/z	INT.	
424.0999	100.0000	**
425.1027	26.3061	***************
426.0975	39.9331	***********************
427.0999	9.8640	******
428.0974	2.7343	**
429.0980	0.4839	
430.0990	0.0626	
431.1001	0.0064	
432.1016	0.0005	

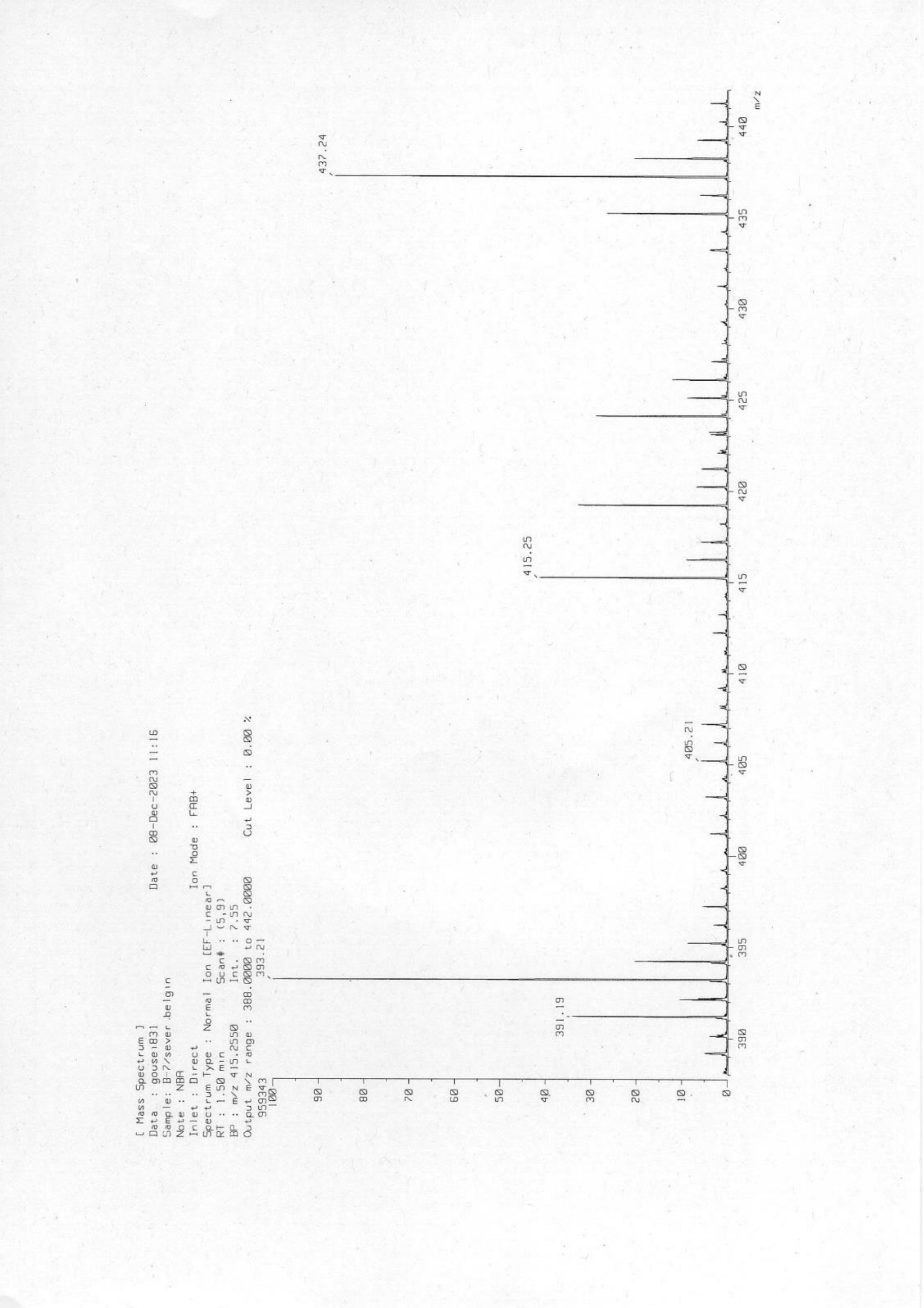
[Mass Spectrum]
Date : 08-Dec-2023 11:16
Sample: B-7/sever.belgin
Note : NBA
Inlet : Direct
Ion Mode : FAB+
Spectrum Type : Normal Ion [EF-Linear]
RT : 1.50 min
Scan# : (5,9)
BP : m/z 415.2550
Int. : 7.55
Output m/z range : 388.0000 to 442.0000
Cut Level : 0.00 %
959343
391.19
393.21
405.21
415.25
437.24
m/z

Figure S13: ^{1}H NMR Spectrum of **BP-1**

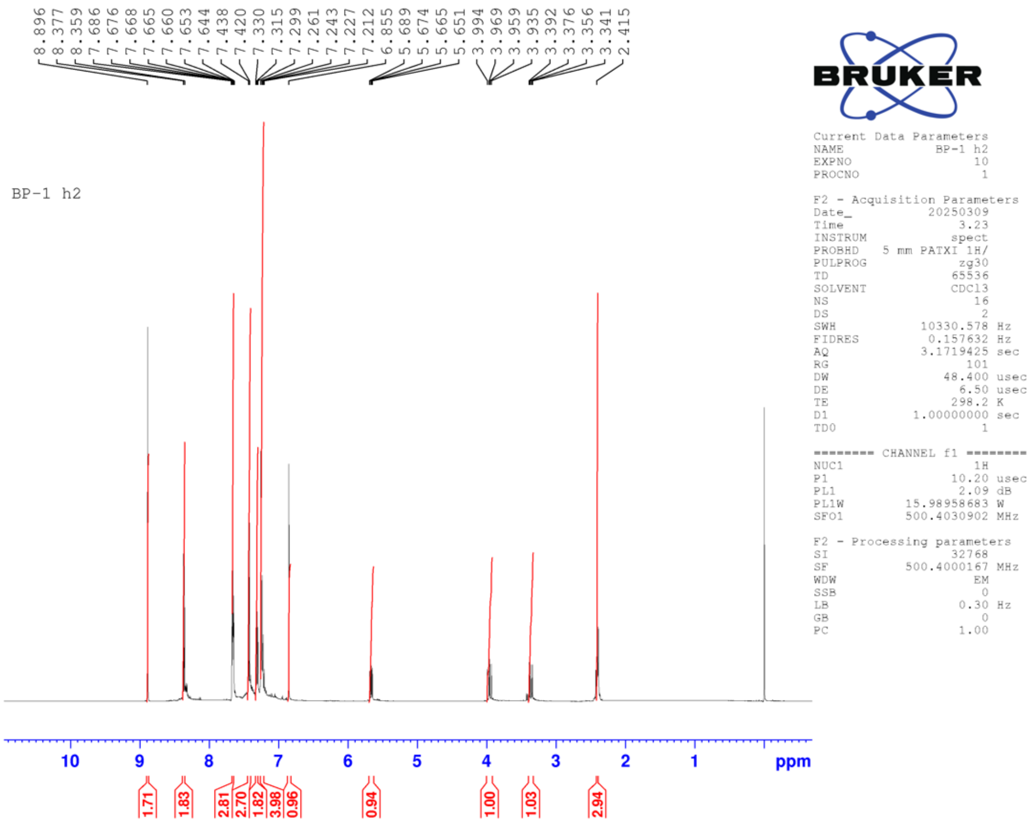

Figure S14: ^{13}C NMR Spectrum of **BP-1**

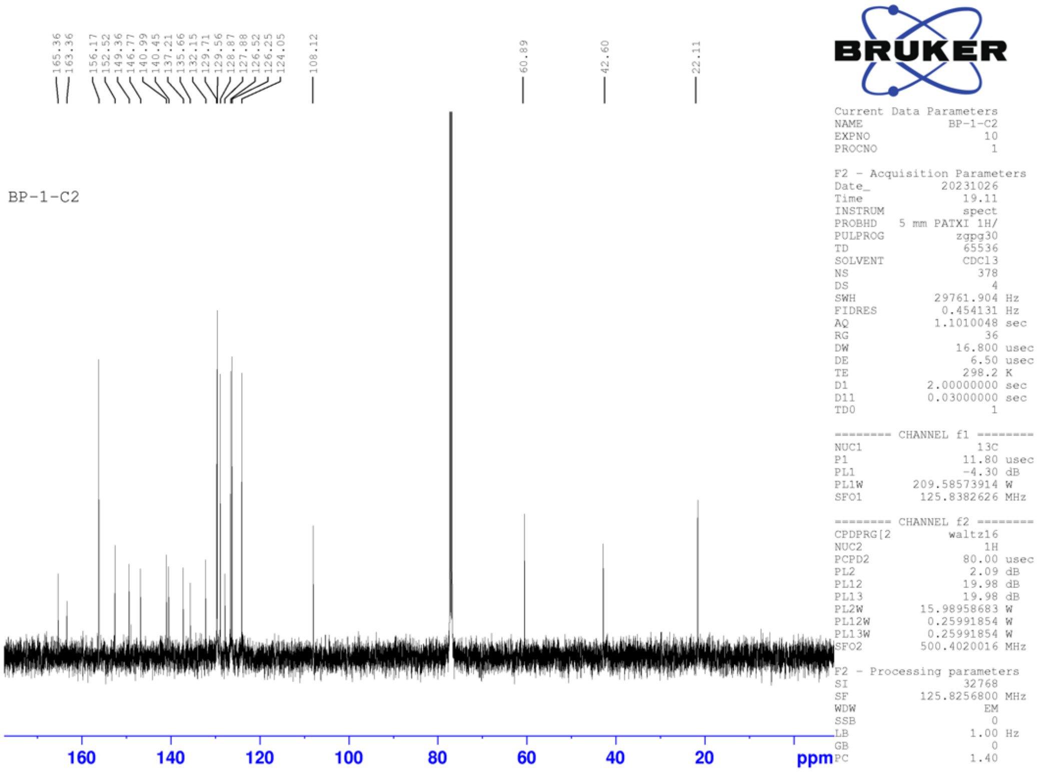

Figure S15: Mass Spectrum of **BP-1**

```
[ Elemental Composition ]                                         Page: 1
Data   : gousei754                   Date : 30-Oct-2023 14:45
Sample: BP-1/sever belgin
Note : NBA
Inlet : Direct                       Ion Mode : FAB+
RT : 1.00 min                        Scan#: (2,8)
Elements : C 100/0, H 100/0, N 6/4, Cl 2/0, S 2/0
Mass Tolerance       : 20ppm, 10mmu if m/z < 500, 20mmu if m/z > 1000
Unsaturation (U.S.) : -0.5 - 100.0

Observed m/z Int%   Err[ppm / mmu]     U.S.  Composition
  507.1302    83.8    -11.2 /  -5.7    30.5  C 34 H 15 N 6
                      -14.8 /  -7.5    25.5  C 33 H 20 N 4 Cl
                      +10.0 /  +5.1    26.0  C 32 H 18 N 5 Cl
                      -17.8 /  -9.0    26.5  C 31 H 19 N 6 S
                       +3.4 /  +1.7    22.0  C 29 H 22 N 5 Cl S
                       -3.3 /  -1.7    18.0  C 26 H 26 N 5 Cl S 2
                      +17.9 /  +9.1    13.5  C 24 H 29 N 4 Cl 2 S 2

  507.2784    29.9     +2.7 /  +1.4     8.5  C 26 H 41 N 6 Cl 2
                       -3.9 /  -2.0     4.5  C 23 H 45 N 6 Cl 2 S
                      -10.6 /  -5.4     0.5  C 20 H 49 N 6 Cl 2 S 2

  508.1355   100.0    -16.0 /  -8.1    30.0  C 34 H 16 N 6
                      -19.6 /  -9.9    25.0  C 33 H 21 N 4 Cl
                       +5.2 /  +2.6    25.5  C 32 H 19 N 5 Cl
                       -1.5 /  -0.7    21.5  C 29 H 23 N 5 Cl S
                      +19.7 / +10.0    17.0  C 27 H 26 N 4 Cl 2 S
                       -8.1 /  -4.1    17.5  C 26 H 27 N 5 Cl S 2
                      +16.7 /  +8.5    18.0  C 25 H 25 N 6 Cl S 2
                      +13.1 /  +6.6    13.0  C 24 H 30 N 4 Cl 2 S 2

  508.2799    10.4     -9.7 /  -4.9     8.0  C 26 H 42 N 6 Cl 2
                      -16.3 /  -8.3     4.0  C 23 H 46 N 6 Cl 2 S
```

```
[ Theoretical Ion Distribution ]                                     Page: 1
Molecular Formula : C29 H23 N5 Cl S
                        (m/z   508.1363, MW   509.0541, U.S. 21.5)
Base Peak :    508.1363, Averaged MW :    509.0543(a),    509.0567(w)

    m/z        INT.
  508.1363 100.0000 ***********************************************************
  509.1392  35.2259 ********************
  510.1344  42.4274 *************************
  511.1366  13.4548 ********
  512.1353   3.6704 **
  513.1355   0.7384
  514.1366   0.1087
  515.1379   0.0124
  516.1394   0.0011
```

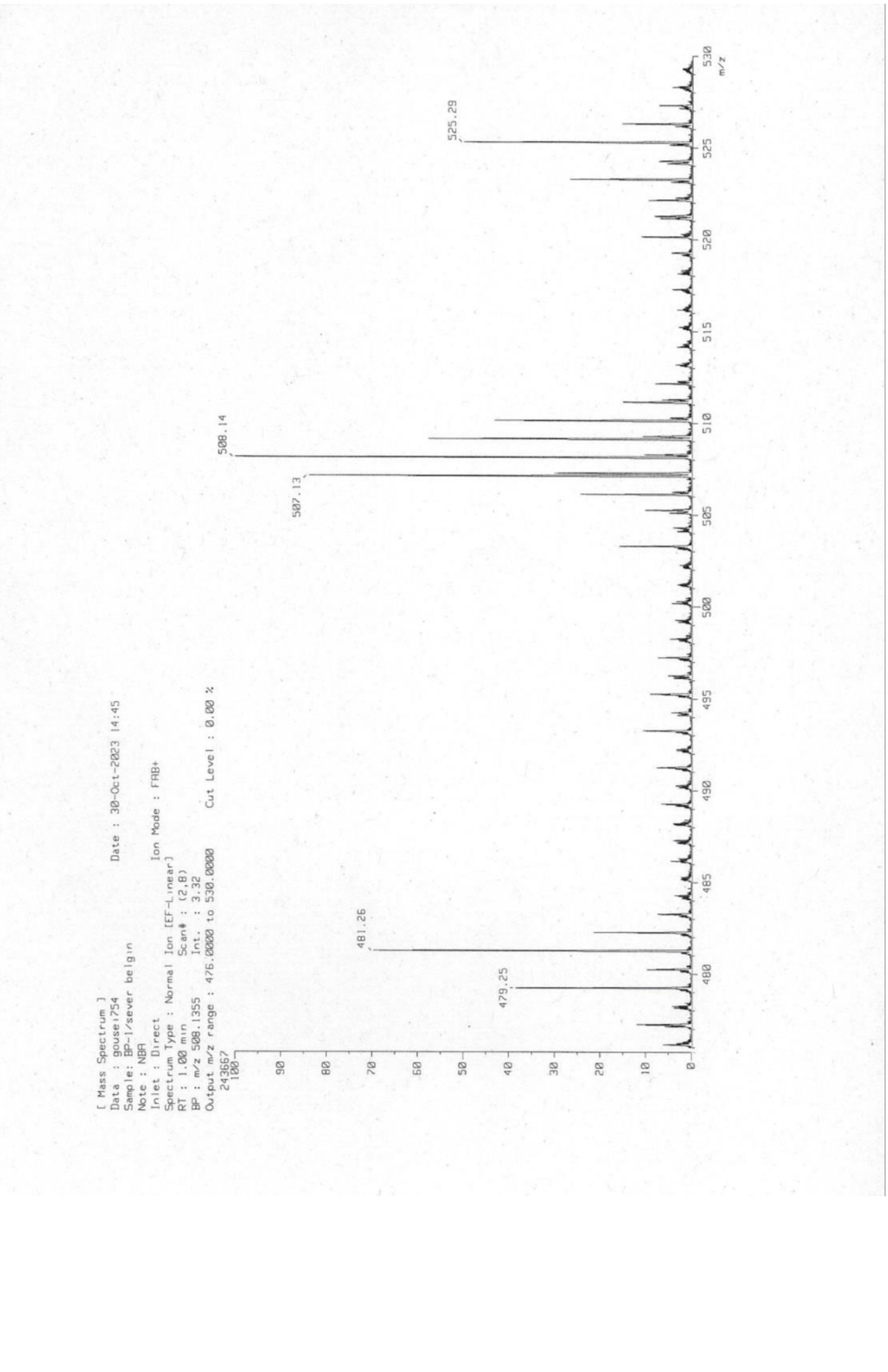
[Mass Spectrum]
Data : gouse1754
Date : 30-Oct-2023 14:45
Sample: BP-1/sever belgin
Note : NBA
Inlet : Direct
Ion Mode : FAB+
Spectrum Type : Normal Ion [EF-Linear]
RT : 1.00 min
Scan# : (2,8)
BP : m/z 508.1355
Int. : 3.32
Output m/z range : 476.0000 to 530.0000
Cut Level : 0.00 %
243667
479.25
481.26
507.13
508.14
525.29
m/z

Figure S16: ^{1}H NMR Spectrum of **BP-2**

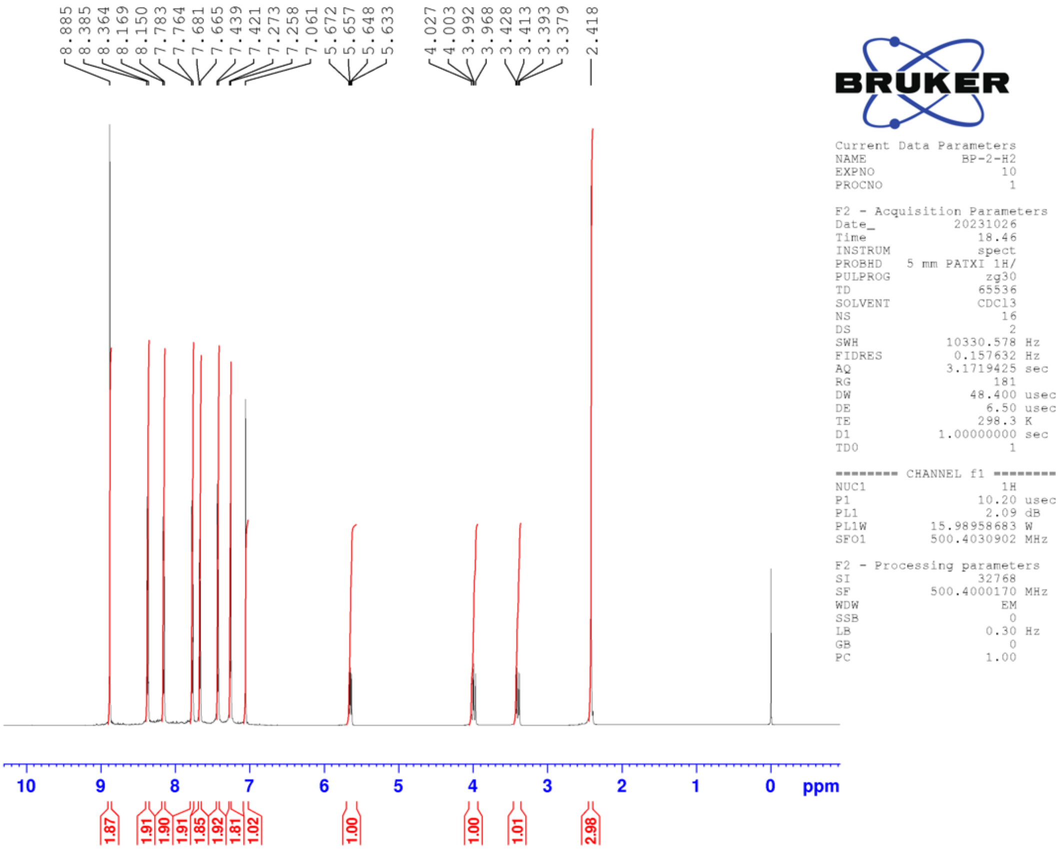

Figure S17: ^{13}C NMR Spectrum of **BP-2**

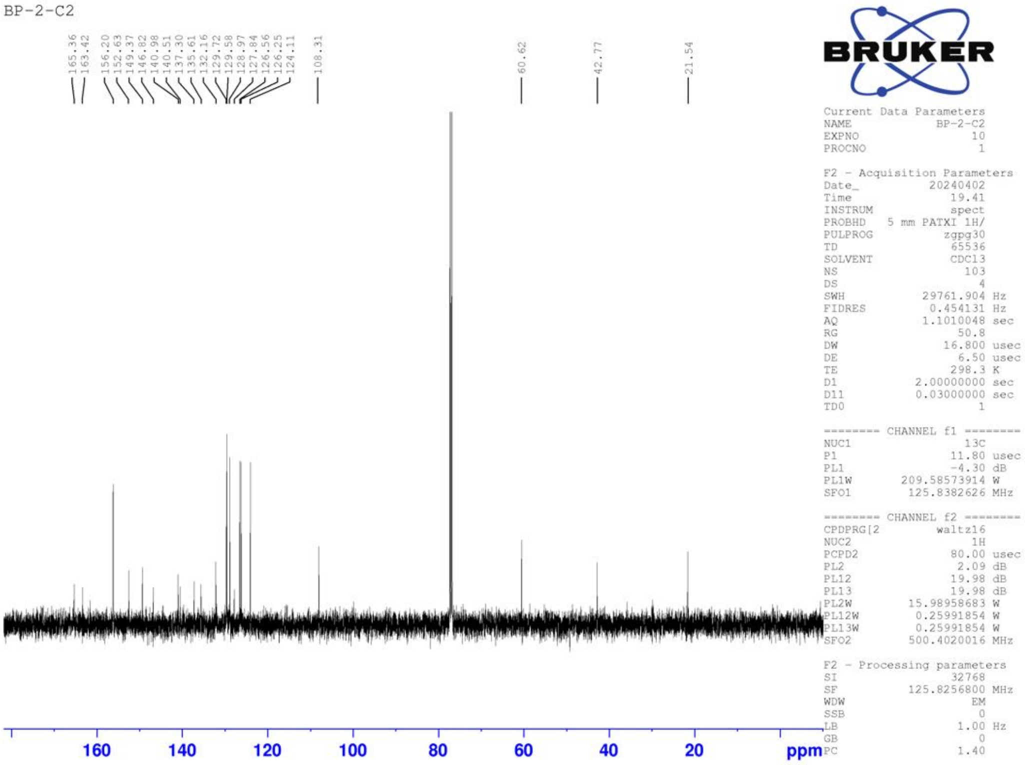

Figure S18: Mass Spectrum of **BP-2**

[Elemental Composition] Page: 1
Data : gousei751 Date : 26-Oct-2023 15:00
Sample: BP-2/sever belgin
Note : CHCl3+NBA
Inlet : Direct Ion Mode : FAB+
RT : 0.88 min Scan#: (3,6)
Elements : C 100/0, H 100/0, O 3/1, N 7/5, Cl 2/0, S 2/0
Mass Tolerance : 20ppm, 10mmu if m/z < 500, 20mmu if m/z > 1000
Unsaturation (U.S.) : -0.5 - 100.0

Observed m/z	Int%	Err[ppm / mmu]	U.S.	Composition
552.1135	83.7	+7.0 / +3.9	31.5	C 35 H 14 O 3 N 5
		-13.4 / -7.4	31.5	C 34 H 14 O 2 N 7
		-16.7 / -9.2	26.5	C 33 H 19 O 2 N 5 Cl
		+6.1 / +3.4	27.0	C 32 H 17 O 2 N 6 Cl
		-17.5 / -9.7	22.0	C 30 H 22 O N 6 Cl 2
		+5.2 / +2.9	22.5	C 29 H 20 O N 7 Cl 2
		+0.9 / +0.5	27.5	C 32 H 18 O 3 N 5 S
		-19.5 / -10.7	27.5	C 31 H 18 O 2 N 7 S
		+0.0 / +0.0	23.0	C 29 H 21 O 2 N 6 Cl S
		+19.5 / +10.7	18.5	C 27 H 24 O 2 N 5 Cl 2 S
		-0.9 / -0.5	18.5	C 26 H 24 O N 7 Cl 2 S
		-5.2 / -2.9	23.5	C 29 H 22 O 3 N 5 S 2
		+17.6 / +9.7	24.0	C 28 H 20 O 3 N 6 S 2
		-6.1 / -3.4	19.0	C 26 H 25 O 2 N 6 Cl S 2
		+16.7 / +9.2	19.5	C 25 H 23 O 2 N 7 Cl S 2
		+13.4 / +7.4	14.5	C 24 H 28 O 2 N 5 Cl 2 S 2
		-7.0 / -3.9	14.5	C 23 H 28 O N 7 Cl 2 S 2
552.3075	20.5	+18.2 / +10.0	17.5	C 33 H 38 O 3 N 5
		-2.2 / -1.2	17.5	C 32 H 38 O 2 N 7
		-5.5 / -3.0	12.5	C 31 H 43 O 2 N 5 Cl
		+17.3 / +9.5	13.0	C 30 H 41 O 2 N 6 Cl
		-6.4 / -3.5	8.0	C 28 H 46 O N 6 Cl 2
		+16.4 / +9.1	8.5	C 27 H 44 O N 7 Cl 2
		+12.1 / +6.7	13.5	C 30 H 42 O 3 N 5 S
		-8.3 / -4.6	13.5	C 29 H 42 O 2 N 7 S
		-11.6 / -6.4	8.5	C 28 H 47 O 2 N 5 Cl S
		+11.2 / +6.2	9.0	C 27 H 45 O 2 N 6 Cl S
		-12.5 / -6.9	4.0	C 25 H 50 O N 6 Cl 2 S
		+10.3 / +5.7	4.5	C 24 H 48 O N 7 Cl 2 S
		+5.9 / +3.3	9.5	C 27 H 46 O 3 N 5 S 2
		-14.4 / -7.9	9.5	C 26 H 46 O 2 N 7 S 2
		-17.7 / -9.8	4.5	C 25 H 51 O 2 N 5 Cl S 2
		+5.1 / +2.8	5.0	C 24 H 49 O 2 N 6 Cl S 2
		-18.6 / -10.3	0.0	C 22 H 54 O N 6 Cl 2 S 2
		+4.2 / +2.3	0.5	C 21 H 52 O N 7 Cl 2 S 2
553.1185	100.0	+1.9 / +1.0	31.0	C 35 H 15 O 3 N 5
		-18.4 / -10.2	31.0	C 34 H 15 O 2 N 7
		+1.0 / +0.6	26.5	C 32 H 18 O 2 N 6 Cl
		+0.1 / +0.1	22.0	C 29 H 21 O N 7 Cl 2
		-4.2 / -2.3	27.0	C 32 H 19 O 3 N 5 S
		+18.5 / +10.2	27.5	C 31 H 17 O 3 N 6 S
		-5.1 / -2.8	22.5	C 29 H 22 O 2 N 6 Cl S
		+17.6 / +9.8	23.0	C 28 H 20 O 2 N 7 Cl S
		+14.3 / +7.9	18.0	C 27 H 25 O 2 N 5 Cl 2 S
		-6.0 / -3.3	18.0	C 26 H 25 O N 7 Cl 2 S
		-10.3 / -5.7	23.0	C 29 H 23 O 3 N 5 S 2
		+12.4 / +6.9	23.5	C 28 H 21 O 3 N 6 S 2
		-11.2 / -6.2	18.5	C 26 H 26 O 2 N 6 Cl S 2
		+11.5 / +6.4	19.0	C 25 H 24 O 2 N 7 Cl S 2
		+8.2 / +4.6	14.0	C 24 H 29 O 2 N 5 Cl 2 S 2
		-12.1 / -6.7	14.0	C 23 H 29 O N 7 Cl 2 S 2
553.3013	12.8	-7.2 / -4.0	17.0	C 33 H 39 O 3 N 5

```
[ Elemental Composition ]                                        Page: 2
                       +15.6 /  +8.6    17.5  C 32 H 37 O 3 N 6
                        -8.0 /  -4.4    12.5  C 30 H 42 O 2 N 6 Cl
                       +14.7 /  +8.1    13.0  C 29 H 40 O 2 N 7 Cl
                       +11.4 /  +6.3     8.0  C 28 H 45 O 2 N 5 Cl 2
                        -8.9 /  -4.9     8.0  C 27 H 45 O N 7 Cl 2
                       -13.3 /  -7.3    13.0  C 30 H 43 O 3 N 5 S
                        +9.5 /  +5.2    13.5  C 29 H 41 O 3 N 6 S
                       -14.1 /  -7.8     8.5  C 27 H 46 O 2 N 6 Cl S
                        +8.6 /  +4.8     9.0  C 26 H 44 O 2 N 7 Cl S
                        +5.3 /  +2.9     4.0  C 25 H 49 O 2 N 5 Cl 2 S
                       -15.0 /  -8.3     4.0  C 24 H 49 O N 7 Cl 2 S
                       +18.8 / +10.4    14.0  C 30 H 43 O N 5 S 2
                       -19.3 / -10.7     9.0  C 27 H 47 O 3 N 5 S 2
                        +3.4 /  +1.9     9.5  C 26 H 45 O 3 N 6 S 2
                        +2.5 /  +1.4     5.0  C 23 H 48 O 2 N 7 Cl S 2
                        -0.8 /  -0.4     0.0  C 22 H 53 O 2 N 5 Cl 2 S 2
```

```
[ Theoretical Ion Distribution ]                                 Page: 1
Molecular Formula : C29 H22 O2 N6 Cl S
                       (m/z   553.1213, MW   554.0517, U.S. 22.5)
Base Peak :   553.1213, Averaged MW :   554.0516(a),   554.0539(w)

    m/z        INT.
  553.1213 100.0000 ************************************************************
  554.1243  35.6544 *********************
  555.1196  42.9796 *************************
  556.1217  13.7795 ********
  557.1206   3.8992 **
  558.1209   0.8089
  559.1219   0.1270
  560.1232   0.0160
  561.1248   0.0017
  562.1265   0.0001
```

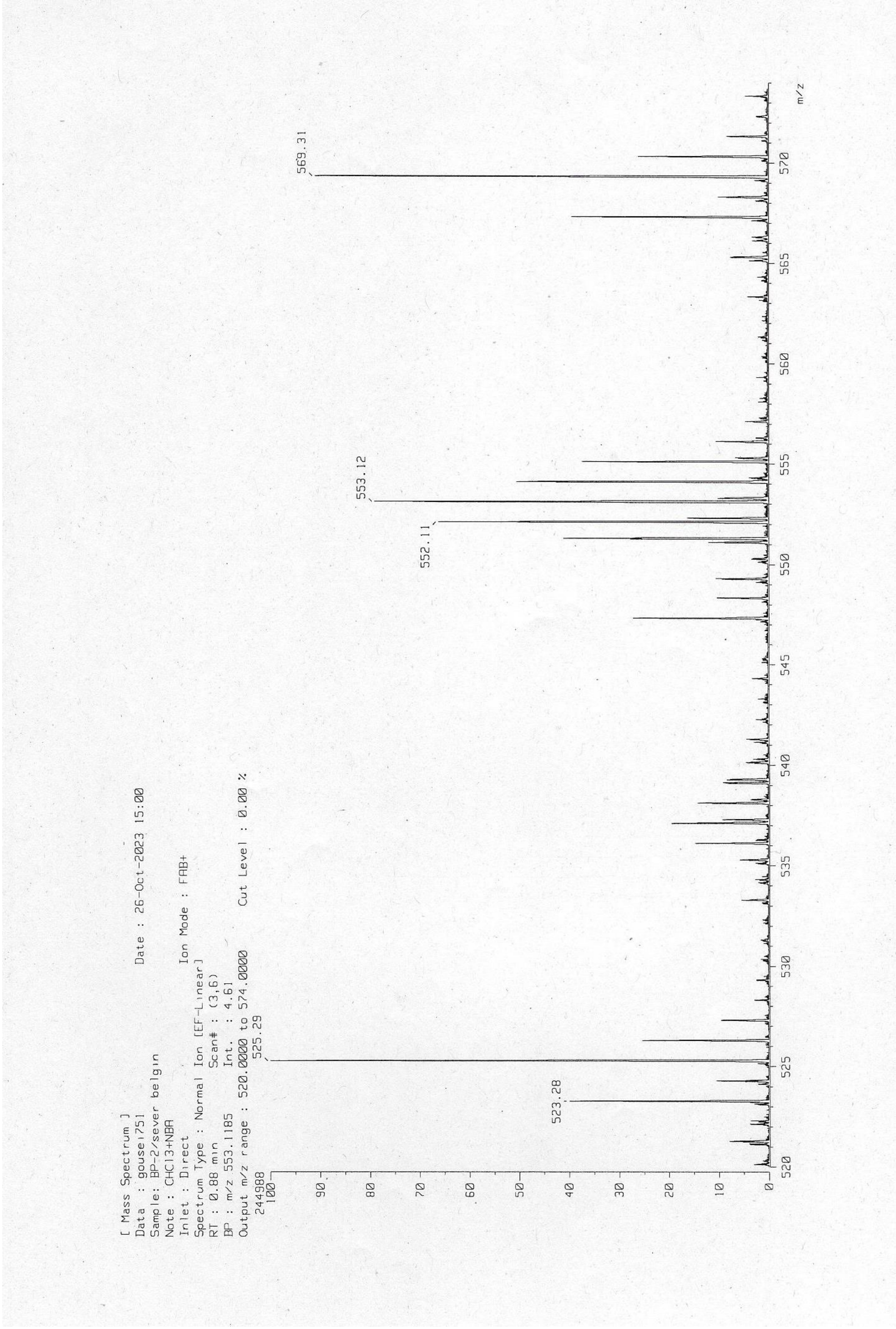
[Mass Spectrum]
Data : gousei751
Date : 26-Oct-2023 15:00
Sample: BP-2/sever belgin
Note : CHCl3+NBA
Inlet : Direct
Ion Mode : FAB+
Spectrum Type : Normal Ion [EF-Linear]
RT : 0.88 min
Scan# : (3,6)
BP : m/z 553.1185
Int. : 4.61
Output m/z range : 520.0000 to 574.0000
Cut Level : 0.00 %
244988
525.29
523.28
552.11
553.12
569.31
m/z

Figure S19: ^{1}H NMR Spectrum of **BP-3**

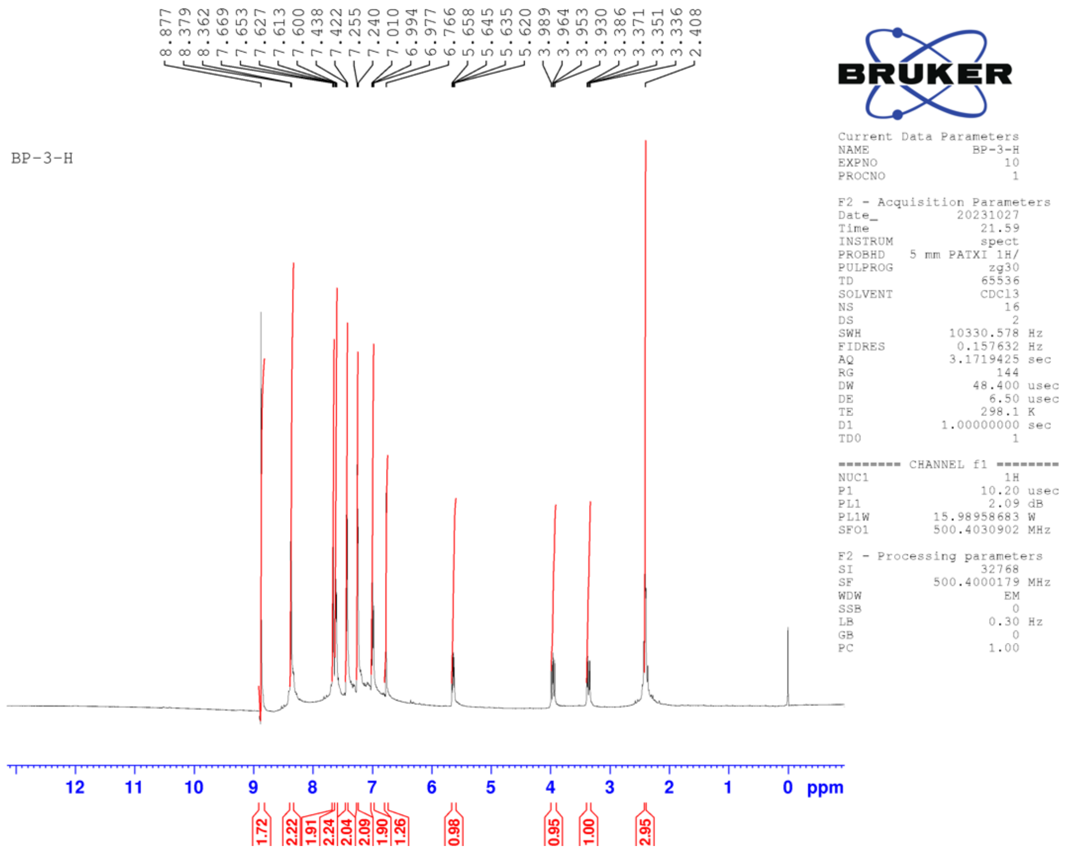

Figure S20: ^{13}C NMR Spectrum of **BP-3**

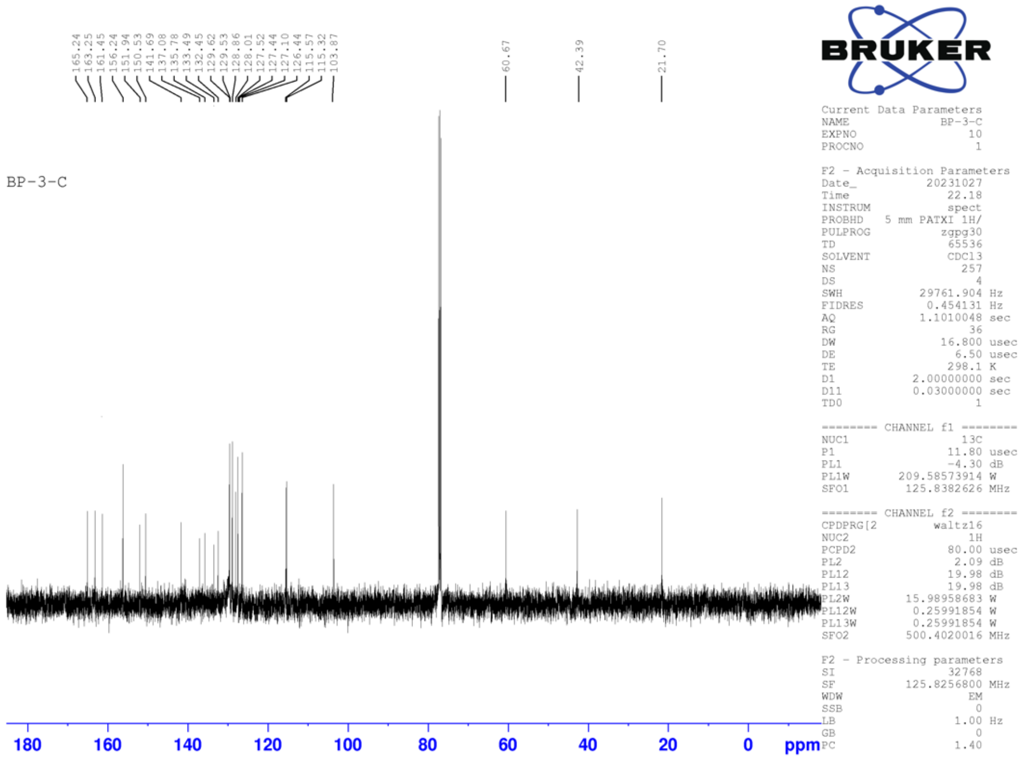

Figure S21: Mass Spectrum of **BP-3**

[Elemental Composition] Page: 1
Data : gousei755 Date : 30-Oct-2023 15:00
Sample: BP-3/sever belgin
Note : NBA
Inlet : Direct Ion Mode : FAB+
RT : 2.13 min Scan#: (7,12)
Elements : C 100/0, H 100/0, N 6/4, Cl 2/0, F 2/0, S 2/0
Mass Tolerance : 20ppm, 10mmu if m/z < 500, 20mmu if m/z > 1000
Unsaturation (U.S.) : -0.5 - 100.0

Observed m/z	Int%	Err[ppm / mmu]	U.S.	Composition
525.1202	89.4	+11.7 / +6.2	34.5	C 38 H 13 N 4
		-11.8 / -6.2	30.5	C 34 H 14 N 6 F
		-15.3 / -8.0	25.5	C 33 H 19 N 4 Cl F
		+8.6 / +4.5	26.0	C 32 H 17 N 5 Cl F
		-18.4 / -9.7	17.0	C 27 H 23 N 5 Cl 2 F 2
		+5.5 / +2.9	17.5	C 26 H 21 N 6 Cl 2 F 2
		+5.3 / +2.8	30.5	C 35 H 17 N 4 S
		-18.3 / -9.6	26.5	C 31 H 18 N 6 F S
		+2.2 / +1.2	22.0	C 29 H 21 N 5 Cl F S
		-0.9 / -0.5	13.5	C 23 H 25 N 6 Cl 2 F 2 S
		-1.1 / -0.6	26.5	C 32 H 21 N 4 S 2
		-4.2 / -2.2	18.0	C 26 H 25 N 5 Cl F S 2
		+19.7 / +10.4	18.5	C 25 H 23 N 6 Cl F S 2
		+16.2 / +8.5	13.5	C 24 H 28 N 4 Cl 2 F S 2
		-7.3 / -3.8	9.5	C 20 H 29 N 6 Cl 2 F 2 S 2
526.1259	100.0	+7.7 / +4.1	34.0	C 38 H 14 N 4
		-15.8 / -8.3	30.0	C 34 H 15 N 6 F
		-19.3 / -10.1	25.0	C 33 H 20 N 4 Cl F
		+4.6 / +2.4	25.5	C 32 H 18 N 5 Cl F
		+1.5 / +0.8	17.0	C 26 H 22 N 6 Cl 2 F 2
		+1.3 / +0.7	30.0	C 35 H 18 N 4 S
		-1.8 / -0.9	21.5	C 29 H 22 N 5 Cl F S
		+18.7 / +9.8	17.0	C 27 H 25 N 4 Cl 2 F S
		-4.9 / -2.6	13.0	C 23 H 26 N 6 Cl 2 F 2 S
		-5.1 / -2.7	26.0	C 32 H 22 N 4 S 2
		+18.8 / +9.9	26.5	C 31 H 20 N 5 S 2
		-8.2 / -4.3	17.5	C 26 H 26 N 5 Cl F S 2
		+15.7 / +8.3	18.0	C 25 H 24 N 6 Cl F S 2
		+12.2 / +6.4	13.0	C 24 H 29 N 4 Cl 2 F S 2
		-11.3 / -5.9	9.0	C 20 H 30 N 6 Cl 2 F 2 S 2

[Theoretical Ion Distribution] Page: 1
Molecular Formula : C29 H22 N5 Cl F S
(m/z 526.1268, MW 527.0446, U.S. 21.5)
Base Peak : 526.1268, Averaged MW : 527.0447(a), 527.0470(w)

m/z	INT.	
526.1268	100.0000	**
527.1298	35.2109	********************
528.1250	42.4221	*************************
529.1272	13.4484	********
530.1259	3.6683	**
531.1261	0.7378	
532.1271	0.1086	
533.1285	0.0124	
534.1300	0.0011	

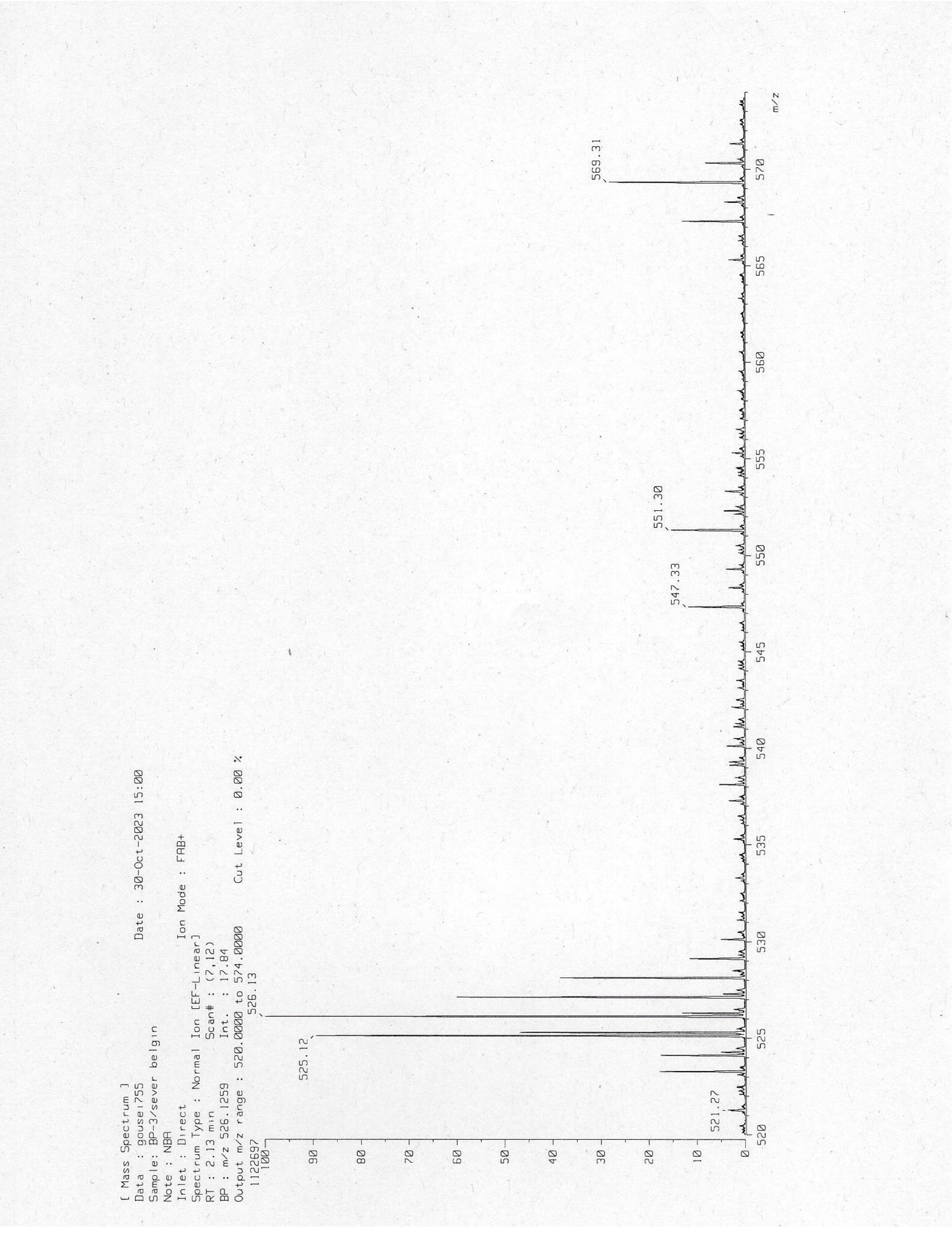
[Mass Spectrum]
Data : gouse1755
Date : 30-Oct-2023 15:00
Sample: BP-3/sever belgin
Note : NBA
Inlet : Direct
Ion Mode : FAB+
Spectrum Type : Normal Ion [EF-Linear]
RT : 2.13 min
Scan# : (7,12)
BP : m/z 526.1259
Int. : 17.84
Output m/z range : 520.0000 to 574.0000
Cut Level : 0.00 %
1122697
526.13
525.12
521.27
547.33
551.30
569.31
m/z

Figure S22: ^{1}H NMR Spectrum of **BP-4**

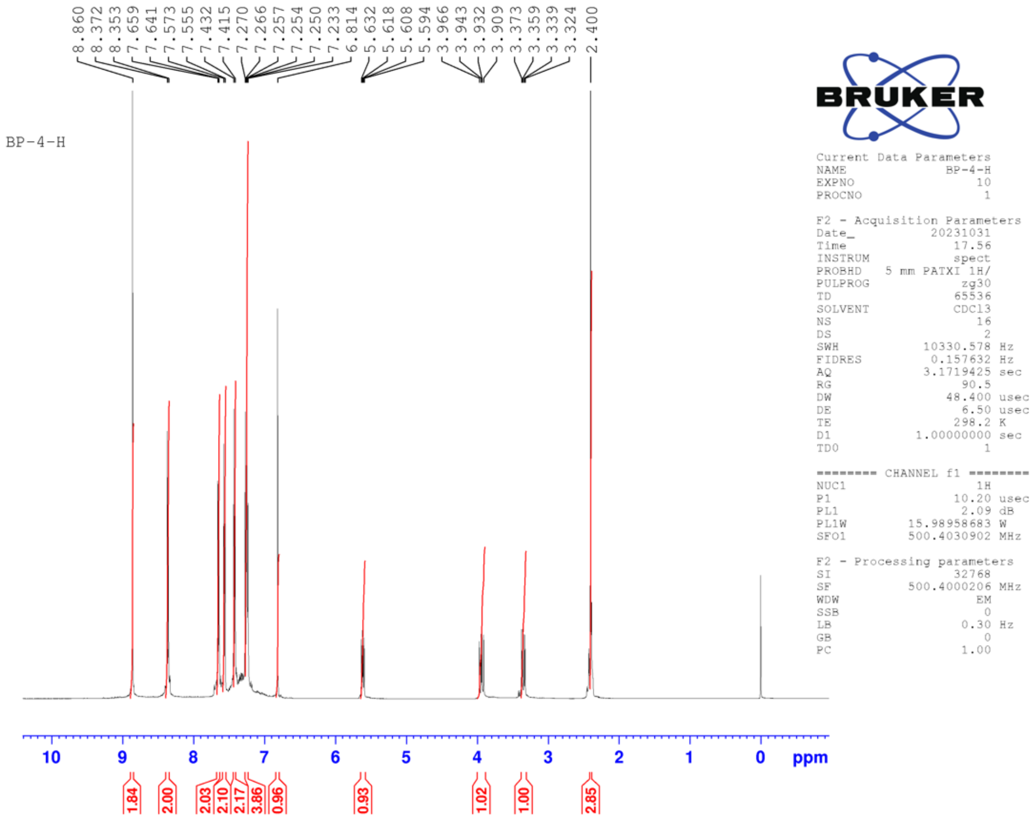

Figure S23: ^{13}C NMR Spectrum of **BP-4**

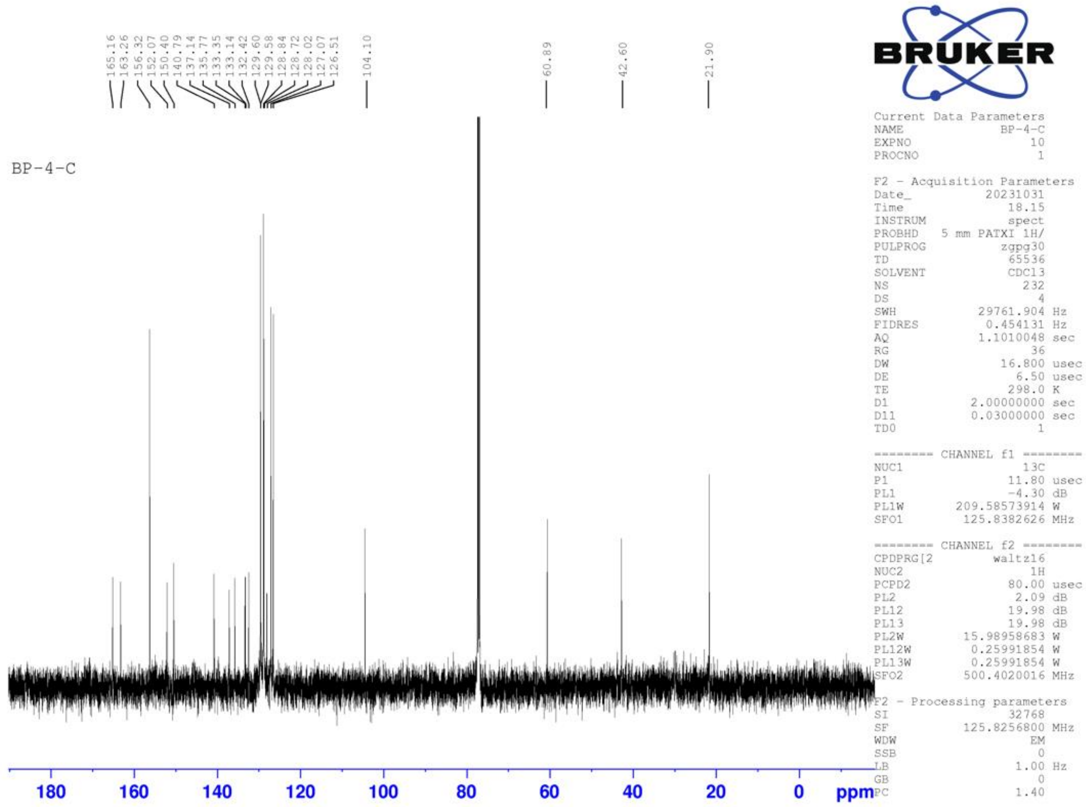

Figure S24: Mass Spectrum of **BP-4**

```
[ Elemental Composition ]                                              Page: 1
Data   : gousei758                       Date : 31-Oct-2023 14:10
Sample: Bp-4/sever belgin
Note : NBA
Inlet : Direct                           Ion Mode : FAB+
RT : 0.88 min                            Scan#: (2,7)
Elements : C 100/0, H 100/0, N 6/4, Cl 3/1, S 2/0
Mass Tolerance      : 20ppm, 10mmu if m/z < 500, 20mmu if m/z > 1000
Unsaturation (U.S.) : -0.5 - 100.0

Observed m/z Int%   Err[ppm / mmu]    U.S.  Composition
  541.0892    77.2    -14.1 /  -7.6   30.5  C 34 H 14 N 6 Cl
                      -17.5 /  -9.4   25.5  C 33 H 19 N 4 Cl 2
                       +5.8 /  +3.1   26.0  C 32 H 17 N 5 Cl 2
                       -0.4 /  -0.2   22.0  C 29 H 21 N 5 Cl 2 S
                      +19.4 / +10.5   17.5  C 27 H 24 N 4 Cl 3 S
                       -6.7 /  -3.6   18.0  C 26 H 25 N 5 Cl 2 S 2
                      +16.6 /  +9.0   18.5  C 25 H 23 N 6 Cl 2 S 2
                      +13.2 /  +7.1   13.5  C 24 H 28 N 4 Cl 3 S 2

  542.0945   100.0    -18.7 / -10.1   30.0  C 34 H 15 N 6 Cl
                       +1.1 /  +0.6   25.5  C 32 H 18 N 5 Cl 2
                       -5.1 /  -2.8   21.5  C 29 H 22 N 5 Cl 2 S
                      +18.1 /  +9.8   22.0  C 28 H 20 N 6 Cl 2 S
                      +14.7 /  +8.0   17.0  C 27 H 25 N 4 Cl 3 S
                      -11.3 /  -6.1   17.5  C 26 H 26 N 5 Cl 2 S 2
                      +11.9 /  +6.4   18.0  C 25 H 24 N 6 Cl 2 S 2
                       +8.5 /  +4.6   13.0  C 24 H 29 N 4 Cl 3 S 2
```

```
[ Theoretical Ion Distribution ]                                     Page: 1
Molecular Formula : C29 H22 N5 Cl2 S
                      (m/z   542.0973, MW   543.4992, U.S. 21.5)
Base Peak :    542.0973, Averaged MW :   543.4990(a),    543.5027(w)

    m/z       INT.
  542.0973 100.0000 ************************************************************
  543.1003  35.2109 *********************
  544.0950  74.4005 ********************************************
  545.0975  24.7083 ***************
  546.0933  17.2342 **********
  547.0949   5.0384 ***
  548.0938   1.2817 *
  549.0938   0.2483
  550.0948   0.0359
  551.0961   0.0040
  552.0976   0.0004
```

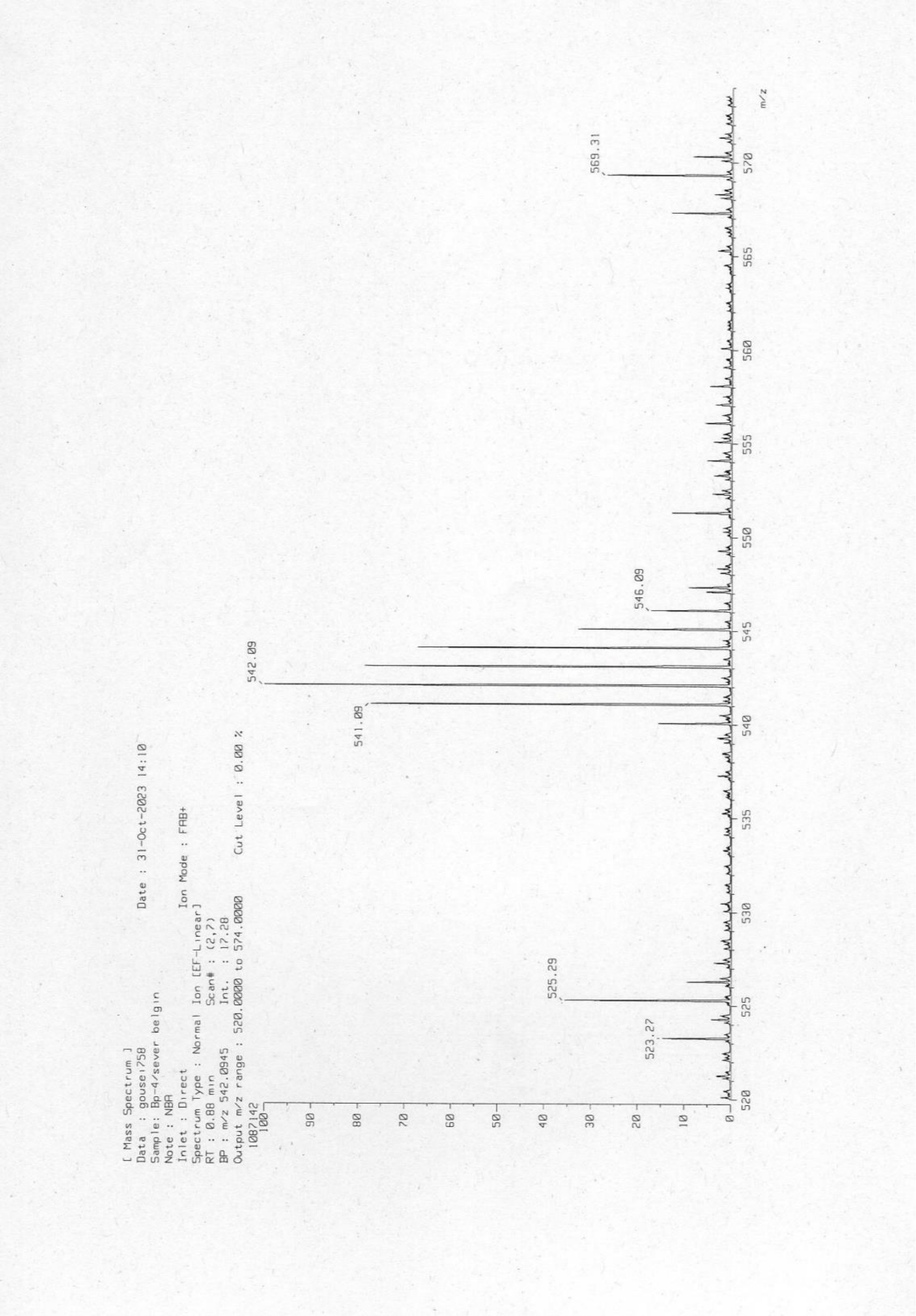
[Mass Spectrum]
Data : gouse1758
Date : 31-Oct-2023 14:10
Sample: Bp-4/sever belgin
Note : NBA
Inlet : Direct
Ion Mode : FAB+
Spectrum Type : Normal Ion [EF-Linear]
RT : 0.88 min
Scan# : (2,7)
BP : m/z 542.0945
Int. : 17.28
Output m/z range : 520.0000 to 574.0000
Cut Level : 0.00 %
1087142
523.27
525.29
541.09
542.09
546.09
569.31
m/z

Figure S25: ^{1}H NMR Spectrum of **BP-5**

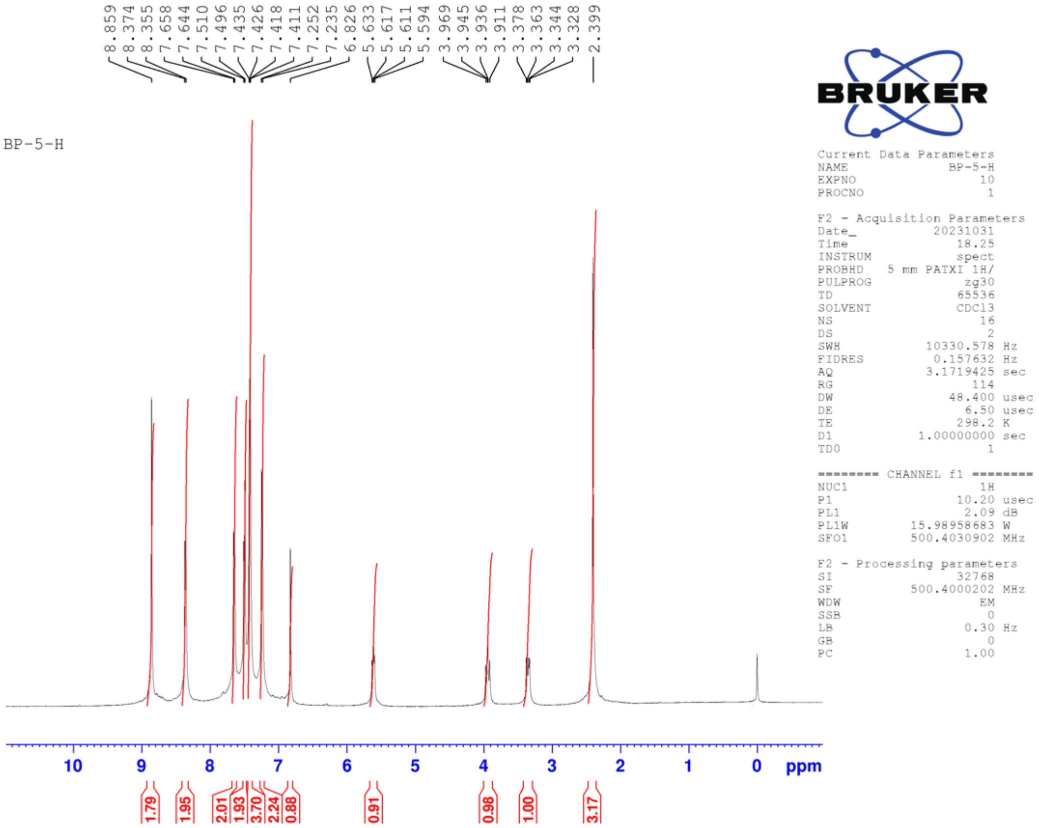

Figure S26: ^{13}C NMR Spectrum of **BP-5**

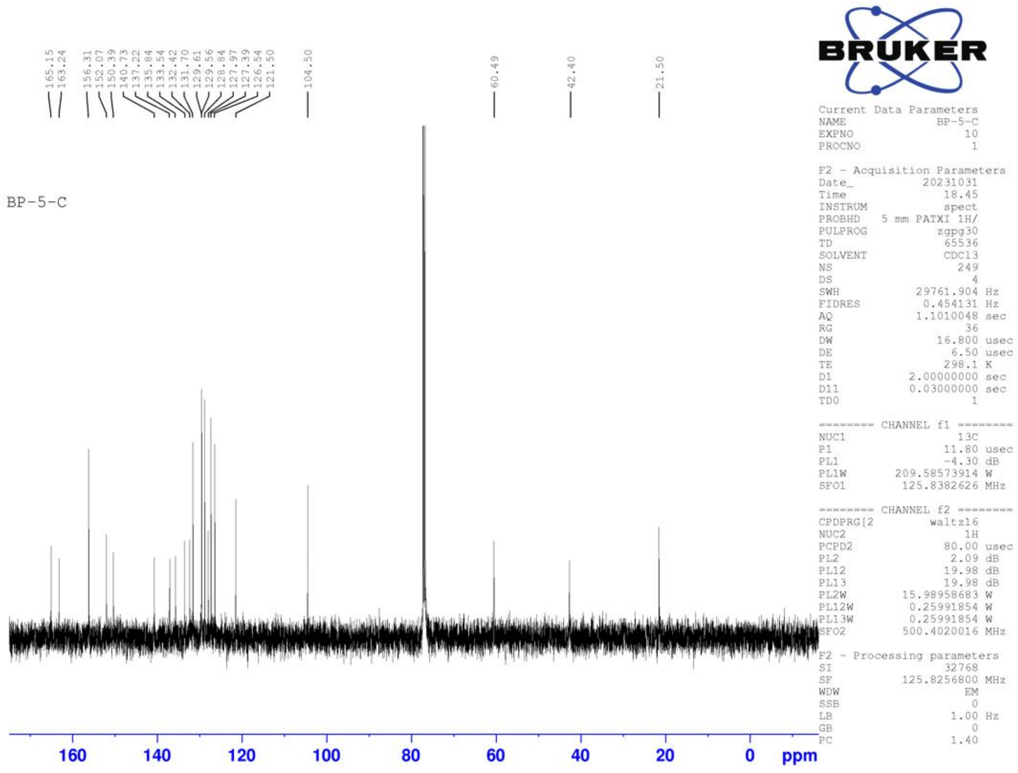

Figure S27: Mass Spectrum of **BP-5**

```
[ Elemental Composition ]                                   Page: 1
Data   : gousei759                  Date : 31-Oct-2023 14:30
Sample: BP-5/sever belgin
Note : NBA
Inlet : Direct                      Ion Mode : FAB+
RT : 1.75 min                       Scan#: (5,11)
Elements : C 100/0, H 100/0, N 6/4, Cl 3/1, Br 3/1, S 2/0
Mass Tolerance       : 20ppm, 10mmu if m/z < 500, 20mmu if m/z > 1000
Unsaturation (U.S.) : -0.5 - 100.0

Observed m/z Int%   Err[ppm / mmu]    U.S.  Composition
  585.0401    57.3    -13.8 /  -8.1    25.5  C 33 H 19 N 4 Cl Br
                       +7.7 /  +4.5    26.0  C 32 H 17 N 5 Cl Br
                      -18.7 / -10.9     5.5  C 20 H 33 N 6 Cl 2 Br 2
                       -0.3 /  -0.2     1.0  C 18 H 36 N 5 Cl 3 Br 2
                      +14.1 /  +8.3    -0.5  C 16 H 37 N 6 Cl Br 3
                      -19.5 / -11.4    21.5  C 30 H 23 N 4 Cl Br S
                       +2.0 /  +1.1    22.0  C 29 H 21 N 5 Cl Br S
                       -3.8 /  -2.2    18.0  C 26 H 25 N 5 Cl Br S 2
                      +17.7 / +10.3    18.5  C 25 H 23 N 6 Cl Br S 2
                      +14.6 /  +8.5    13.5  C 24 H 28 N 4 Cl 2 Br S 2

  586.0435    72.8     +0.2 /  +0.1    25.5  C 32 H 18 N 5 Cl Br
                      +18.5 / +10.9    21.0  C 30 H 21 N 4 Cl 2 Br
                       -7.9 /  -4.6     0.5  C 18 H 37 N 5 Cl 3 Br 2
                      +13.6 /  +8.0     1.0  C 17 H 35 N 6 Cl 3 Br 2
                       -5.6 /  -3.3    21.5  C 29 H 22 N 5 Cl Br S
                      +15.9 /  +9.3    22.0  C 28 H 20 N 6 Cl Br S
                      +12.8 /  +7.5    17.0  C 27 H 25 N 4 Cl 2 Br S
                      -11.3 /  -6.6    17.5  C 26 H 26 N 5 Cl Br S 2
                      +10.1 /  +5.9    18.0  C 25 H 24 N 6 Cl Br S 2
                       +7.0 /  +4.1    13.0  C 24 H 29 N 4 Cl 2 Br S 2
```

```
[ Theoretical Ion Distribution ]                            Page: 1
Molecular Formula : C29 H22 N5 Cl Br S
                     (m/z   586.0468, MW   587.9502, U.S. 21.5)
Base Peak :    588.0448, Averaged MW :   587.9498(a),   587.9536(w)

    m/z        INT.
  586.0468  71.5821 ******************************************
  587.0497  25.2047 ***************
  588.0448 100.0000 **********************************************************
  589.0475  34.1452 ********************
  590.0432  32.1658 *******************
  591.0451   9.8927 ******
  592.0439   2.6321 **
  593.0440   0.5226
  594.0451   0.0764
  595.0464   0.0087
  596.0479   0.0008
```

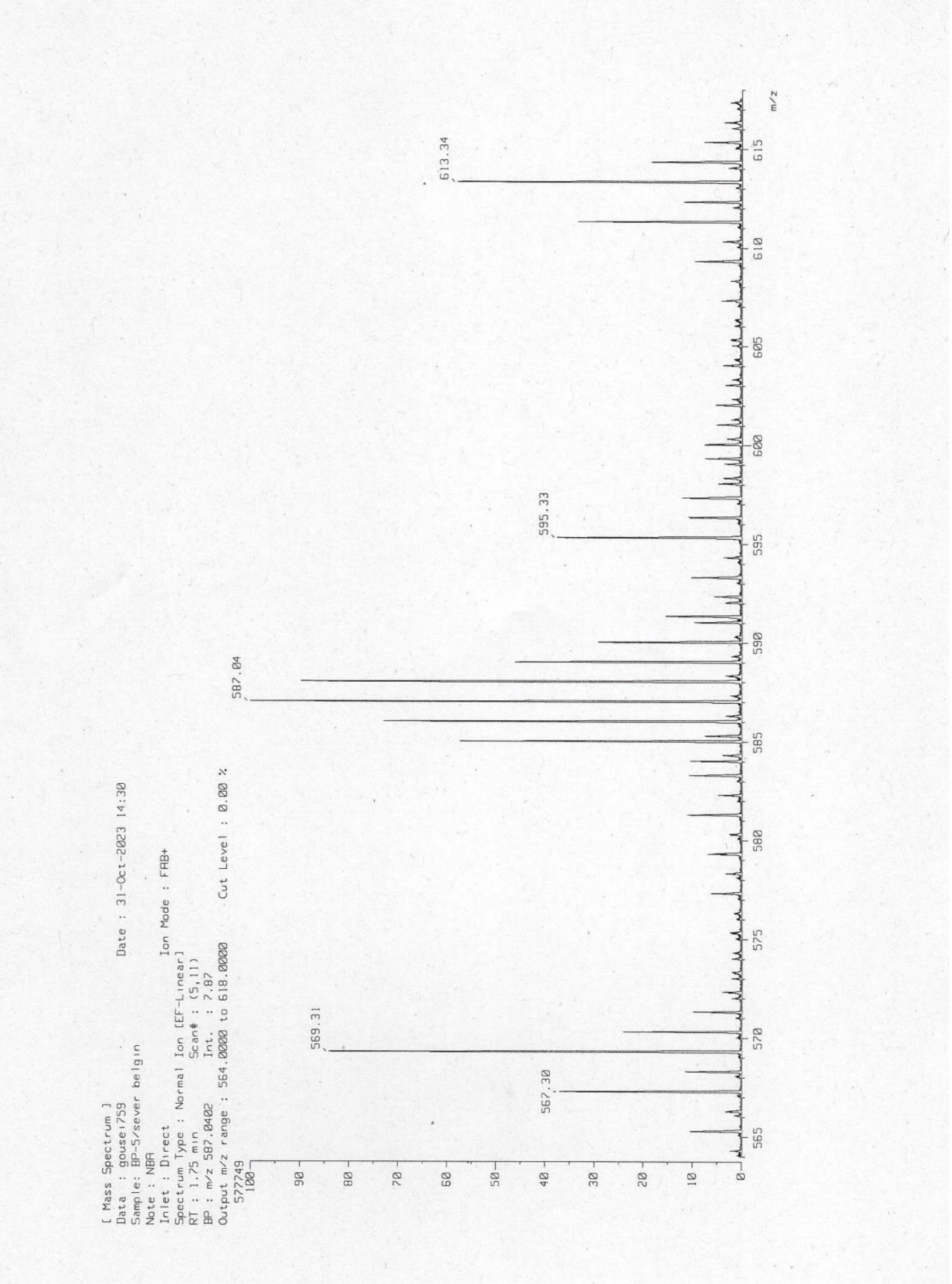
[Mass Spectrum]
Data : gouse1759
Date : 31-Oct-2023 14:30
Sample: BP-5/sever belgin
Note : NBA
Inlet : Direct
Ion Mode : FAB+
Spectrum Type : Normal Ion [EF-Linear]
RT : 1.75 min
Scan# : (5,11)
BP : m/z 587.0402
Int. : 7.87
Output m/z range : 564.0000 to 618.0000
Cut Level : 0.00 %
577749
567.30
569.31
587.04
595.33
613.34
m/z

Figure S28. ^{1}H NMR Spectrum of **BP-6**

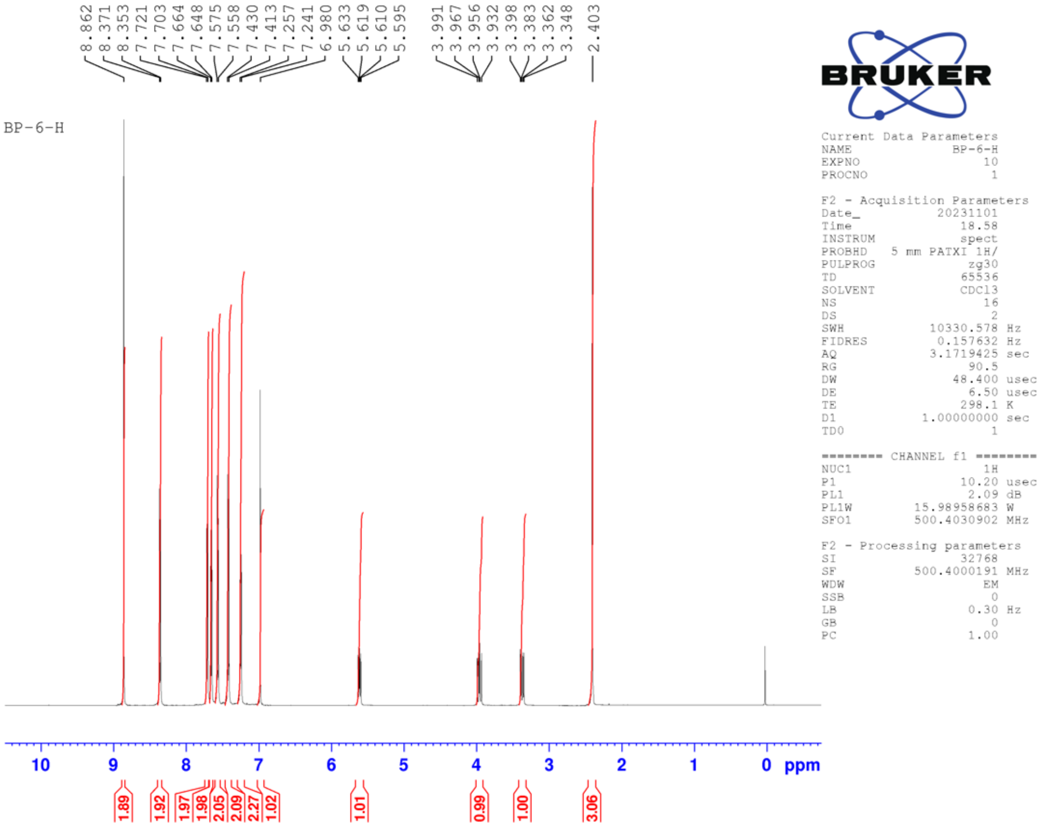

Figure S29. ^{13}C NMR Spectrum of **BP-6**

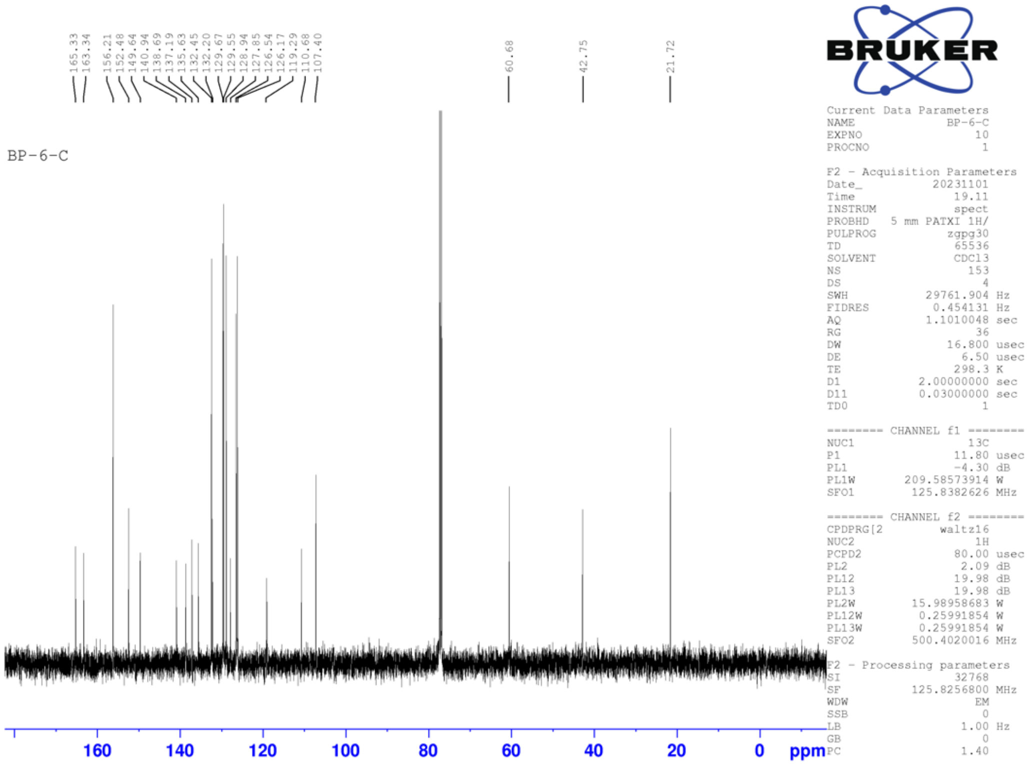

Figure S30: Mass Spectrum of **BP-6**

[Elemental Composition] Page: 1

Data : gousei762 Date : 01-Nov-2023 14:13
Sample: BP-6/sever belgin
Note : NBA
Inlet : Direct Ion Mode : FAB+
RT : 1.13 min Scan#: (3,8)
Elements : C 100/0, H 100/0, N 7/5, Cl 2/0, S 2/0
Mass Tolerance : 20ppm, 10mmu if m/z < 500, 20mmu if m/z > 1000
Unsaturation (U.S.) : -0.5 - 100.0

Observed m/z	Int%	Err[ppm / mmu]	U.S.	Composition
533.1304	100.0	-16.0 / -8.5	32.0	C 35 H 15 N 7
		-19.4 / -10.4	27.0	C 34 H 20 N 5 Cl
		+4.2 / +2.2	27.5	C 33 H 18 N 6 Cl
		-2.2 / -1.1	23.5	C 30 H 22 N 6 Cl S
		+18.0 / +9.6	19.0	C 28 H 25 N 5 Cl 2 S
		-8.5 / -4.5	19.5	C 27 H 26 N 6 Cl S 2
		+15.1 / +8.1	20.0	C 26 H 24 N 7 Cl S 2
		+11.7 / +6.2	15.0	C 25 H 29 N 5 Cl 2 S 2
532.1250	95.2	-11.4 / -6.1	32.5	C 35 H 14 N 7
		-14.8 / -7.9	27.5	C 34 H 19 N 5 Cl
		+8.8 / +4.7	28.0	C 33 H 17 N 6 Cl
		-17.7 / -9.4	28.5	C 32 H 18 N 7 S
		+2.5 / +1.3	24.0	C 30 H 21 N 6 Cl S
		-3.9 / -2.1	20.0	C 27 H 25 N 6 Cl S 2
		+19.8 / +10.5	20.5	C 26 H 23 N 7 Cl S 2
		+16.3 / +8.7	15.5	C 25 H 28 N 5 Cl 2 S 2

[Theoretical Ion Distribution] Page: 1

Molecular Formula : C30 H22 N6 Cl S
(m/z 533.1315, MW 534.0639, U.S. 23.5)
Base Peak : 533.1315, Averaged MW : 534.0640(a), 534.0664(w)

m/z	INT.	
533.1315	100.0000	**
534.1344	36.6905	**********************
535.1298	42.9472	**************************
536.1318	14.0775	********
537.1307	3.8691	**
538.1308	0.7926	
539.1319	0.1197	
540.1332	0.0140	
541.1347	0.0013	
542.1364	0.0001	

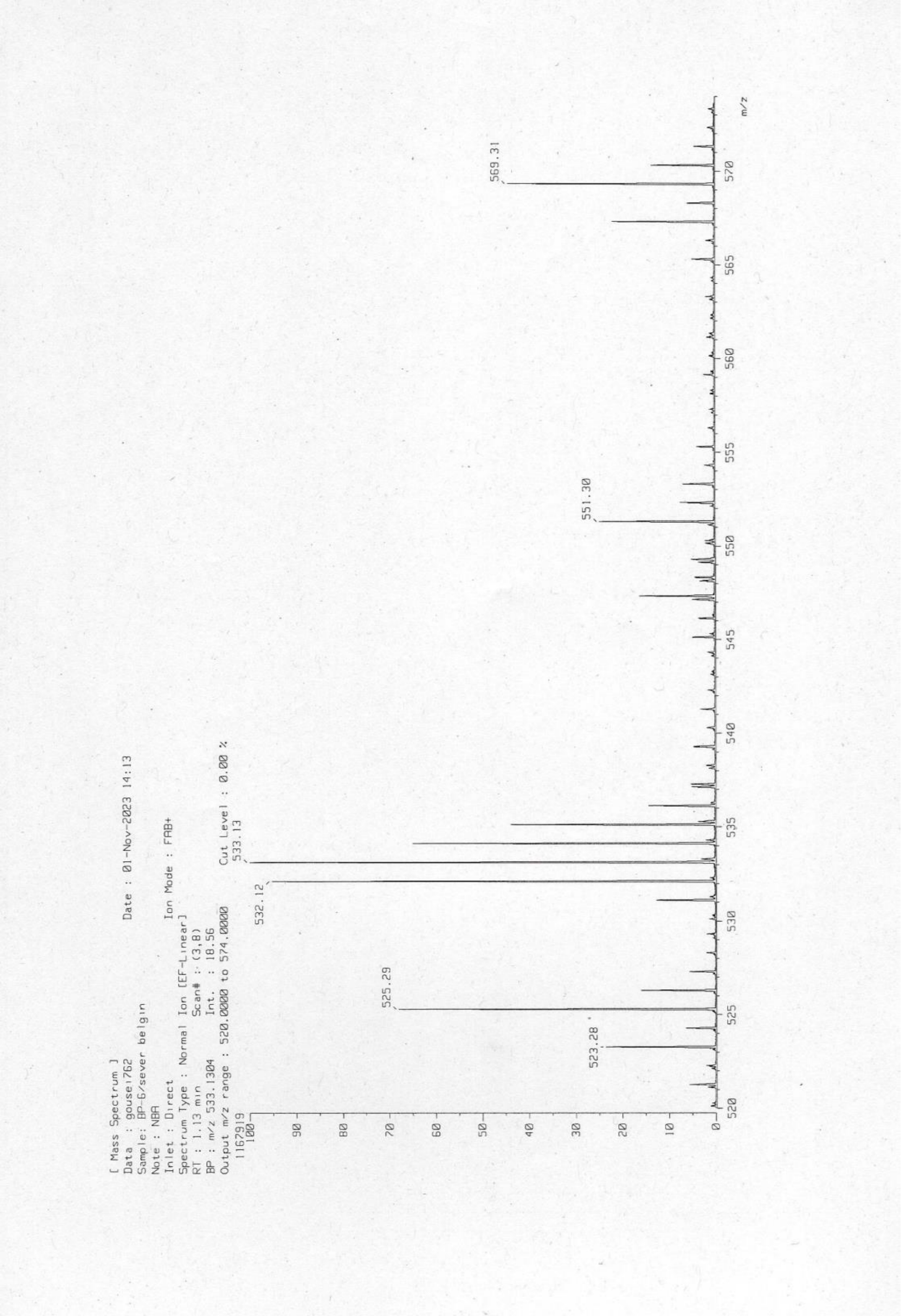
[Mass Spectrum]
Data : gouse1762
Sample: BP-6/sever belgin
Note : NBA
Inlet : Direct
Ion Mode : FAB+
Spectrum Type : Normal Ion [EF-Linear]
RT : 1.13 min
Scan# : (3,8)
BP : m/z 533.1304
Int. : 18.56
Output m/z range : 520.0000 to 574.0000
Cut Level : 0.00 %
Date : 01-Nov-2023 14:13
1167919
523.28
525.29
532.12
533.13
551.30
569.31
m/z

Figure S31: ^{1}H NMR Spectrum of **BP-7**

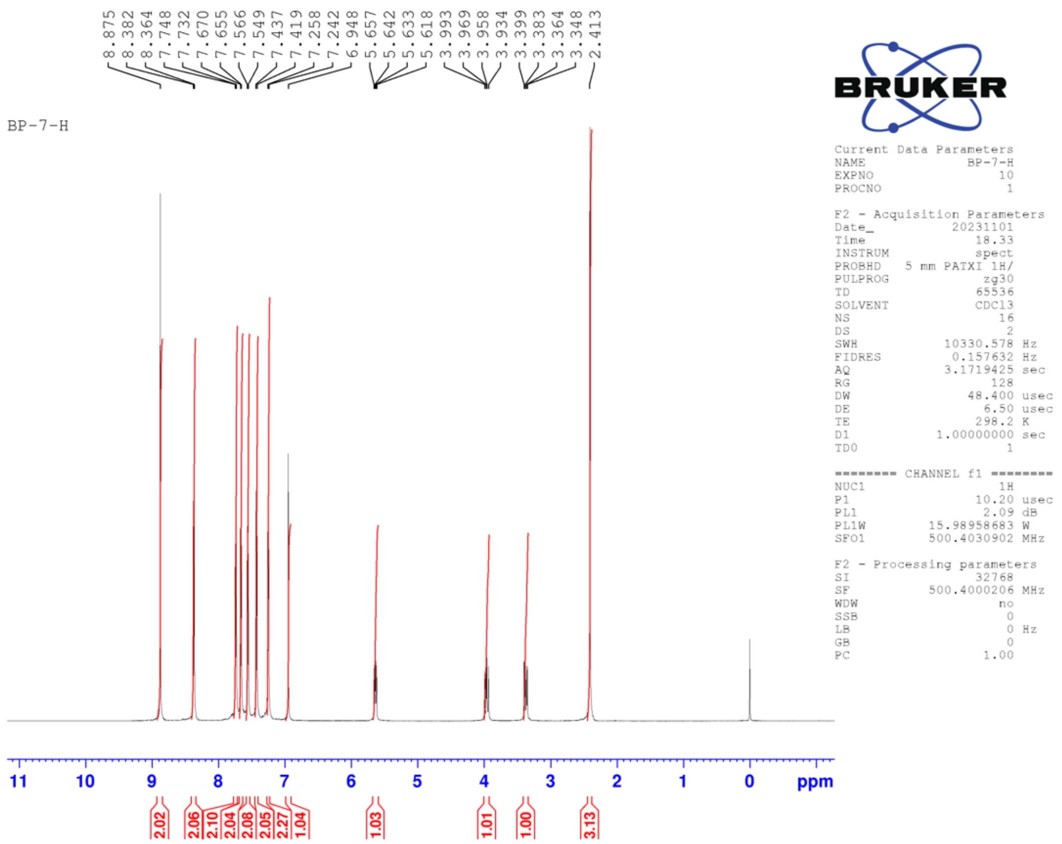

Figure S32: ^{13}C NMR Spectrum of **BP-7**

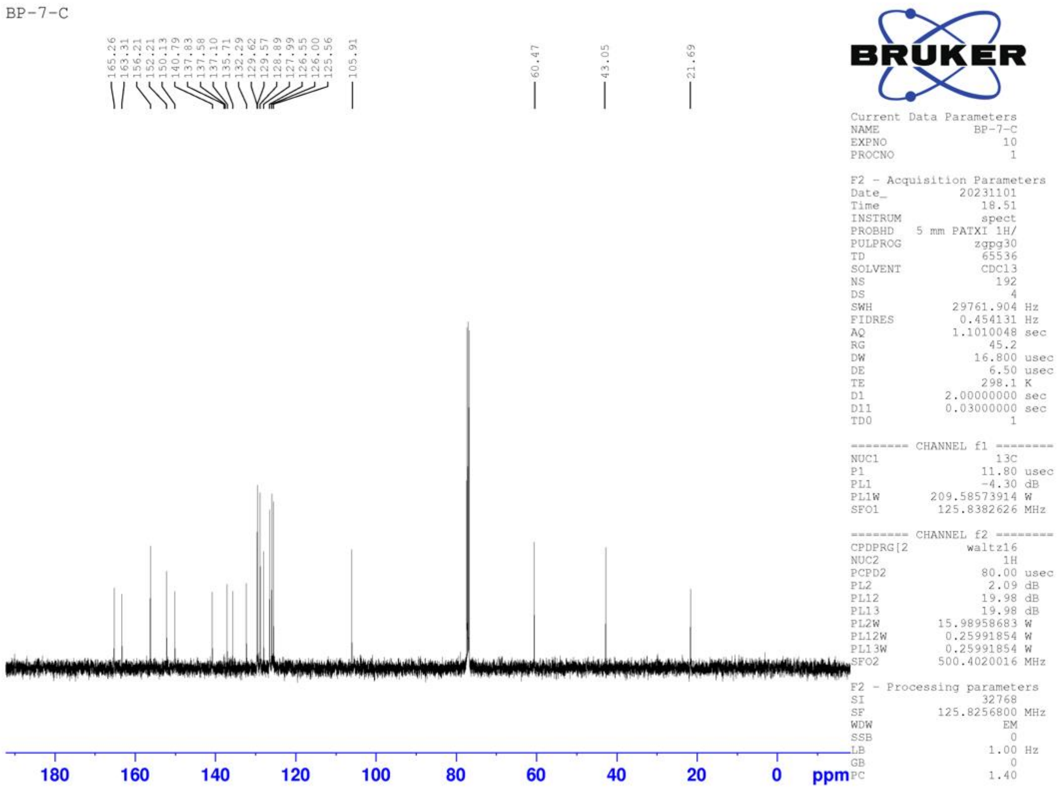

Figure S33: Mass Spectrum of **BP-7**

[Elemental Composition] Page: 1

Data : gousei763 Date : 01-Nov-2023 14:28
Sample: BP-7/sever belgin
Note : NBA
Inlet : Direct Ion Mode : FAB+
RT : 1.25 min Scan#: (3,9)
Elements : C 100/0, H 100/0, N 6/4, Cl 2/0, F 4/2, S 2/0
Mass Tolerance : 20ppm, 10mmu if m/z < 500, 20mmu if m/z > 1000
Unsaturation (U.S.) : -0.5 - 100.0

Observed m/z	Int%	Err[ppm / mmu]	U.S.	Composition
575.1167	94.8	+10.2 / +5.9	34.5	C 39 H 13 N 4 F 2
		-11.3 / -6.5	30.5	C 35 H 14 N 6 F 3
		-14.5 / -8.3	25.5	C 34 H 19 N 4 Cl F 3
		+7.4 / +4.2	26.0	C 33 H 17 N 5 Cl F 3
		-17.3 / -10.0	17.0	C 28 H 23 N 5 Cl 2 F 4
		+4.5 / +2.6	17.5	C 27 H 21 N 6 Cl 2 F 4
		+4.3 / +2.5	30.5	C 36 H 17 N 4 F 2 S
		-17.2 / -9.9	26.5	C 32 H 18 N 6 F 3 S
		+1.5 / +0.9	22.0	C 30 H 21 N 5 Cl F 3 S
		-1.3 / -0.8	13.5	C 24 H 25 N 6 Cl 2 F 4 S
		-1.5 / -0.9	26.5	C 33 H 21 N 4 F 2 S 2
		-4.4 / -2.5	18.0	C 27 H 25 N 5 Cl F 3 S 2
		+17.5 / +10.1	18.5	C 26 H 23 N 6 Cl F 3 S 2
		+14.3 / +8.2	13.5	C 25 H 28 N 4 Cl 2 F 3 S 2
		-7.2 / -4.1	9.5	C 21 H 29 N 6 Cl 2 F 4 S 2
576.1225	100.0	+6.7 / +3.8	34.0	C 39 H 14 N 4 F 2
		-14.8 / -8.5	30.0	C 35 H 15 N 6 F 3
		-18.0 / -10.4	25.0	C 34 H 20 N 4 Cl F 3
		+3.8 / +2.2	25.5	C 33 H 18 N 5 Cl F 3
		+1.0 / +0.6	17.0	C 27 H 22 N 6 Cl 2 F 4
		+0.8 / +0.5	30.0	C 36 H 18 N 4 F 2 S
		-2.0 / -1.2	21.5	C 30 H 22 N 5 Cl F 3 S
		+19.8 / +11.4	22.0	C 29 H 20 N 6 Cl F 3 S
		+16.6 / +9.6	17.0	C 28 H 25 N 4 Cl 2 F 3 S
		-4.8 / -2.8	13.0	C 24 H 26 N 6 Cl 2 F 4 S
		-5.0 / -2.9	26.0	C 33 H 22 N 4 F 2 S 2
		+16.8 / +9.7	26.5	C 32 H 20 N 5 F 2 S 2
		-7.9 / -4.5	17.5	C 27 H 26 N 5 Cl F 3 S 2
		+14.0 / +8.0	18.0	C 26 H 24 N 6 Cl F 3 S 2
		+10.8 / +6.2	13.0	C 25 H 29 N 4 Cl 2 F 3 S 2
		-10.7 / -6.2	9.0	C 21 H 30 N 6 Cl 2 F 4 S 2

[Theoretical Ion Distribution] Page: 1

Molecular Formula : C30 H22 N5 Cl F3 S
(m/z 576.1237, MW 577.0524, U.S. 21.5)
Base Peak : 576.1237, Averaged MW : 577.0525(a), 577.0547(w)

m/z	INT.	
576.1237	100.0000	**
577.1266	36.3231	*********************
578.1219	42.8138	*************************
579.1240	13.9203	********
580.1229	3.8179	**
581.1230	0.7786	
582.1241	0.1168	
583.1255	0.0136	
584.1270	0.0013	

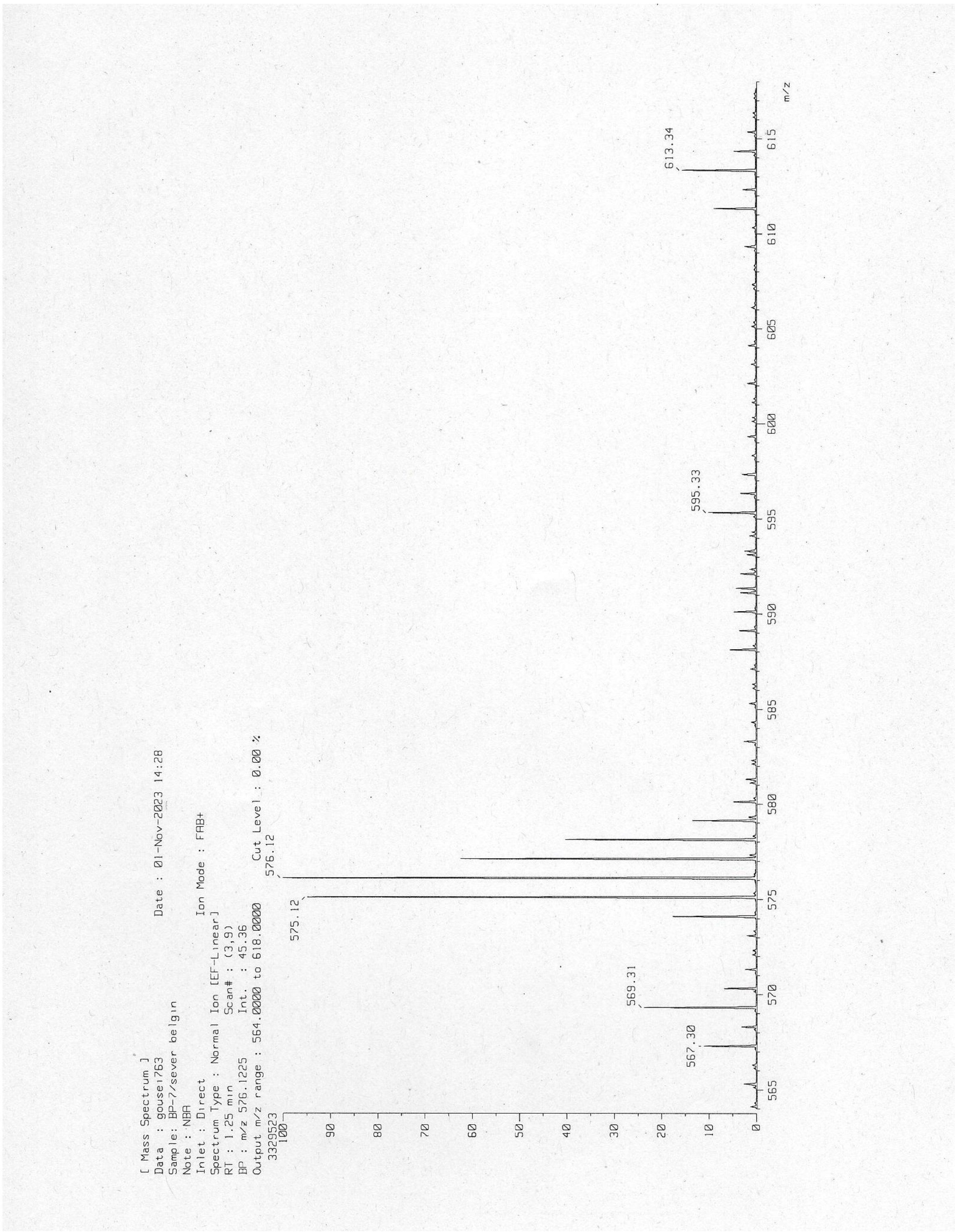
[Mass Spectrum]
Data : gousei763
Date : 01-Nov-2023 14:28
Sample: BP-7/sever belgin
Note : NBA
Inlet : Direct
Ion Mode : FAB+
Spectrum Type : Normal Ion [EF-Linear]
RT : 1.25 min
Scan# : (3,9)
BP : m/z 576.1225
Int. : 45.36
Output m/z range : 564.0000 to 618.0000
Cut Level : 0.00 %
3329523
576.12
575.12
569.31
567.30
595.33
613.34
m/z

Figure S34: ^{1}H NMR Spectrum of **BP-8**

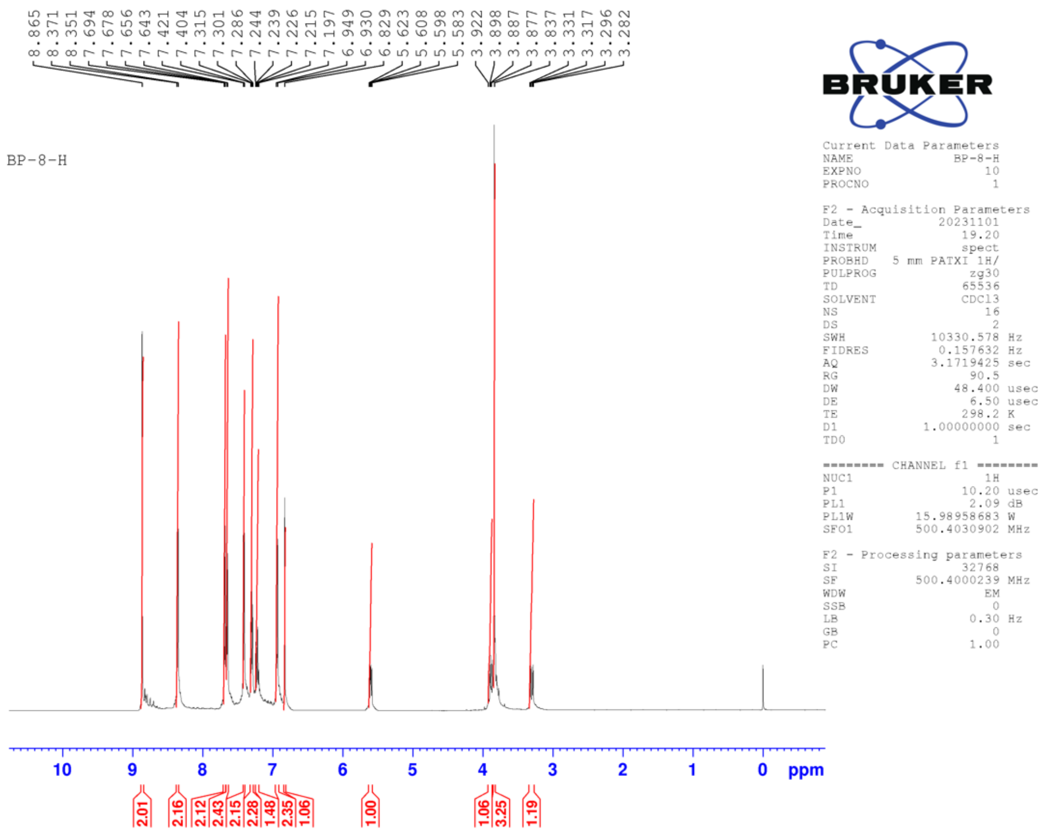

Figure S35: ^{13}C NMR Spectrum of **BP-8**

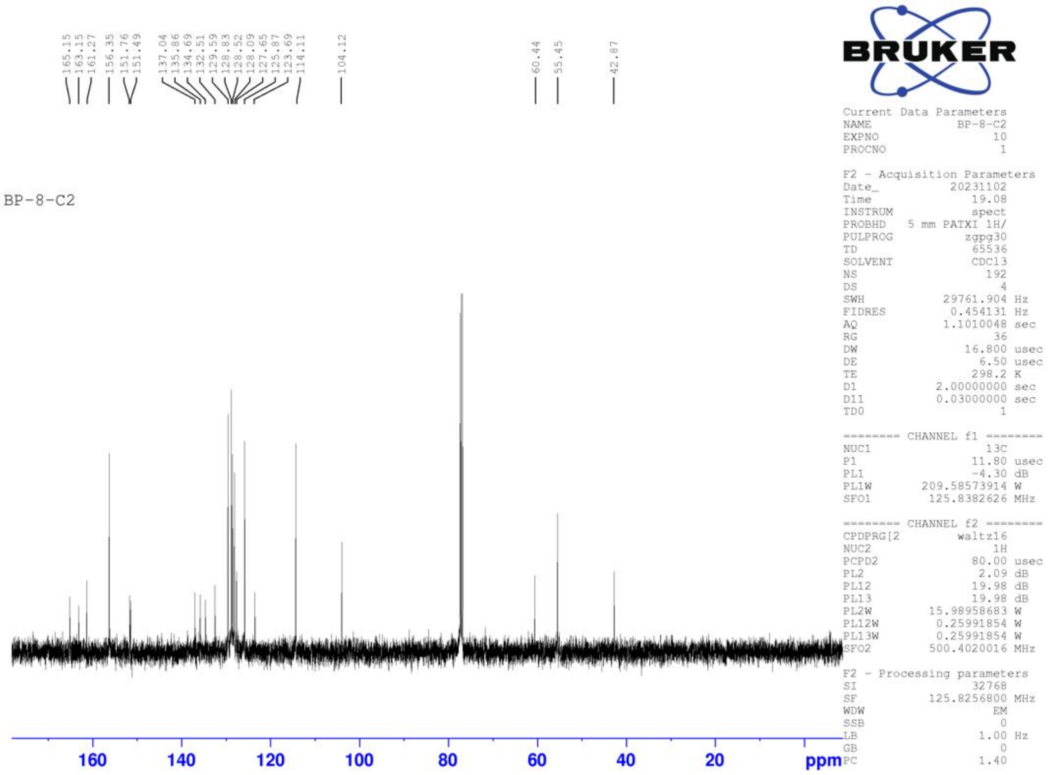

Figure S36: Mass Spectrum of **BP-8**

[Elemental Composition] Page: 1
Data : gousei766 Date : 02-Nov-2023 14:41
Sample: BP-8/sever belgin
Note : NBA
Inlet : Direct Ion Mode : FAB+
RT : 1.25 min Scan#: (4,8)
Elements : C 100/0, H 100/0, O 2/0, N 6/4, Cl 2/0, S 2/0
Mass Tolerance : 20ppm, 10mmu if m/z < 500, 20mmu if m/z > 1000
Unsaturation (U.S.) : -0.5 - 100.0

Observed m/z	Int%	Err[ppm / mmu]	U.S.	Composition
523.1227	66.1	+6.1 / +3.2	30.5	C 35 H 15 O 2 N 4
		-15.4 / -8.1	30.5	C 34 H 15 O N 6
		-18.9 / -9.9	25.5	C 33 H 20 O N 4 Cl
		+5.1 / +2.7	26.0	C 32 H 18 O N 5 Cl
		-19.8 / -10.4	21.0	C 30 H 23 N 5 Cl 2
		+4.2 / +2.2	21.5	C 29 H 21 N 6 Cl 2
		-0.4 / -0.2	26.5	C 32 H 19 O 2 N 4 S
		-1.3 / -0.7	22.0	C 29 H 22 O N 5 Cl S
		+19.2 / +10.1	17.5	C 27 H 25 O N 4 Cl 2 S
		-2.2 / -1.2	17.5	C 26 H 25 N 6 Cl 2 S
		-6.8 / -3.6	22.5	C 29 H 23 O 2 N 4 S 2
		+17.2 / +9.0	23.0	C 28 H 21 O 2 N 5 S 2
		-7.7 / -4.1	18.0	C 26 H 26 O N 5 Cl S 2
		+16.3 / +8.5	18.5	C 25 H 24 O N 6 Cl S 2
		+12.8 / +6.7	13.5	C 24 H 29 O N 4 Cl 2 S 2
		-8.7 / -4.5	13.5	C 23 H 29 N 6 Cl 2 S 2
524.1298	80.9	+4.8 / +2.5	30.0	C 35 H 16 O 2 N 4
		-16.7 / -8.7	30.0	C 34 H 16 O N 6
		+3.8 / +2.0	25.5	C 32 H 19 O N 5 Cl
		+2.9 / +1.5	21.0	C 29 H 22 N 6 Cl 2
		-1.7 / -0.9	26.0	C 32 H 20 O 2 N 4 S
		-2.6 / -1.4	21.5	C 29 H 23 O N 5 Cl S
		+17.9 / +9.4	17.0	C 27 H 26 O N 4 Cl 2 S
		-3.5 / -1.9	17.0	C 26 H 26 N 6 Cl 2 S
		-8.1 / -4.2	22.0	C 29 H 24 O 2 N 4 S 2
		+15.9 / +8.3	22.5	C 28 H 22 O 2 N 5 S 2
		-9.0 / -4.7	17.5	C 26 H 27 O N 5 Cl S 2
		+15.0 / +7.8	18.0	C 25 H 25 O N 6 Cl S 2
		+11.5 / +6.0	13.0	C 24 H 30 O N 4 Cl 2 S 2
		-10.0 / -5.2	13.0	C 23 H 30 N 6 Cl 2 S 2

[Theoretical Ion Distribution] Page: 1
Molecular Formula : C29 H23 O N5 Cl S
(m/z 524.1312, MW 525.0535, U.S. 21.5)
Base Peak : 524.1312, Averaged MW : 525.0536(a), 525.0559(w)

m/z	INT.	
524.1312	100.0000	**
525.1342	35.2640	*********************
526.1294	42.6413	**************************
527.1315	13.5416	********
528.1303	3.7605	**
529.1306	0.7667	
530.1317	0.1163	
531.1330	0.0139	
532.1346	0.0014	
533.1363	0.0001	

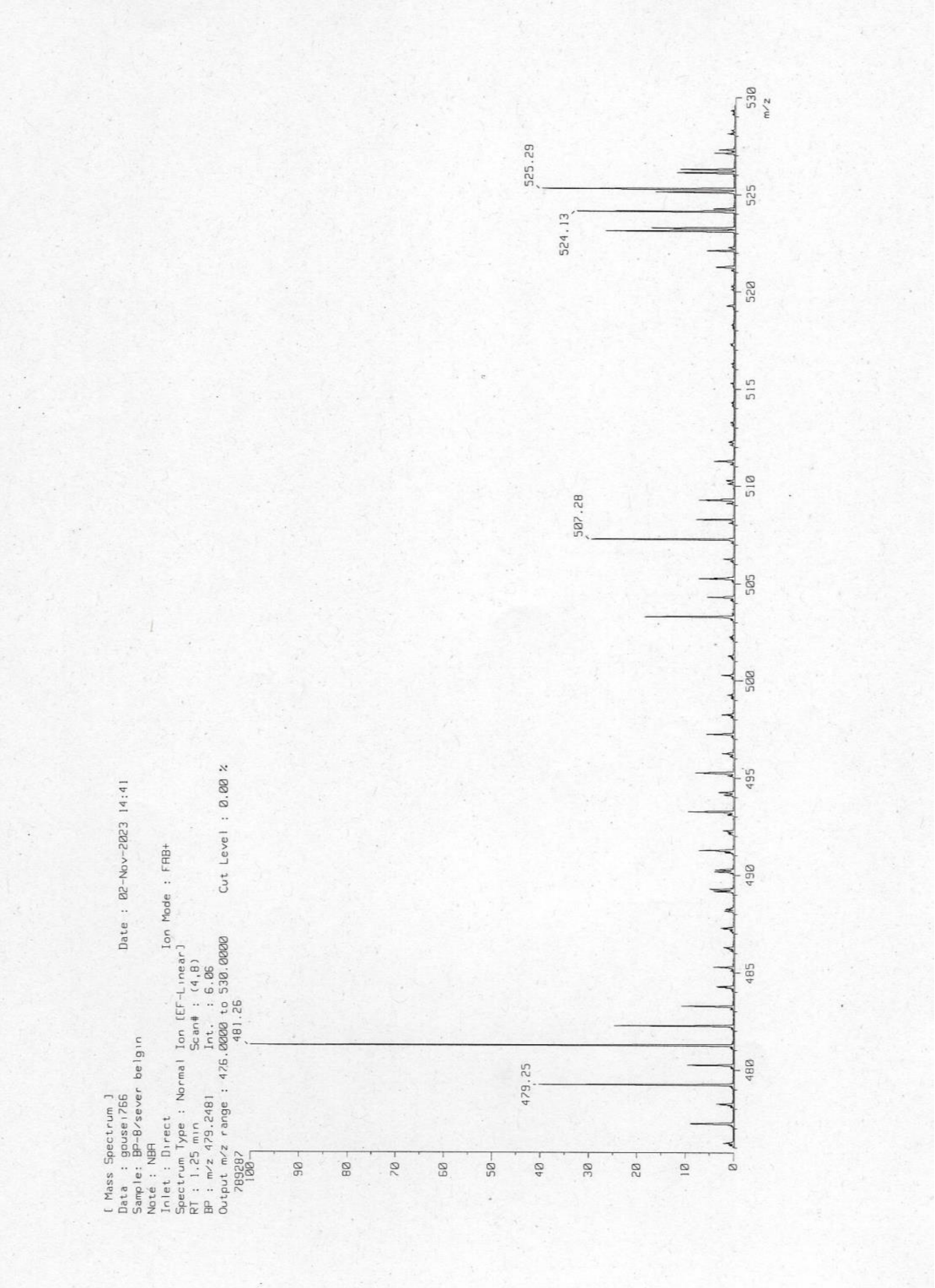
[Mass Spectrum]
Data : gouse1766
Date : 02-Nov-2023 14:41
Sample: BP-8/sever belgin
Note : NBA
Inlet : Direct
Ion Mode : FAB+
Spectrum Type : Normal Ion [EF-Linear]
RT : 1.25 min
Scan# : (4,8)
BP : m/z 479.2481
Int. : 6.06
Output m/z range : 476.0000 to 530.0000
Cut Level : 0.00 %
789287
479.25
481.26
507.28
524.13
525.29
m/z

Figure S37: ^{1}H NMR Spectrum of **BP-9**

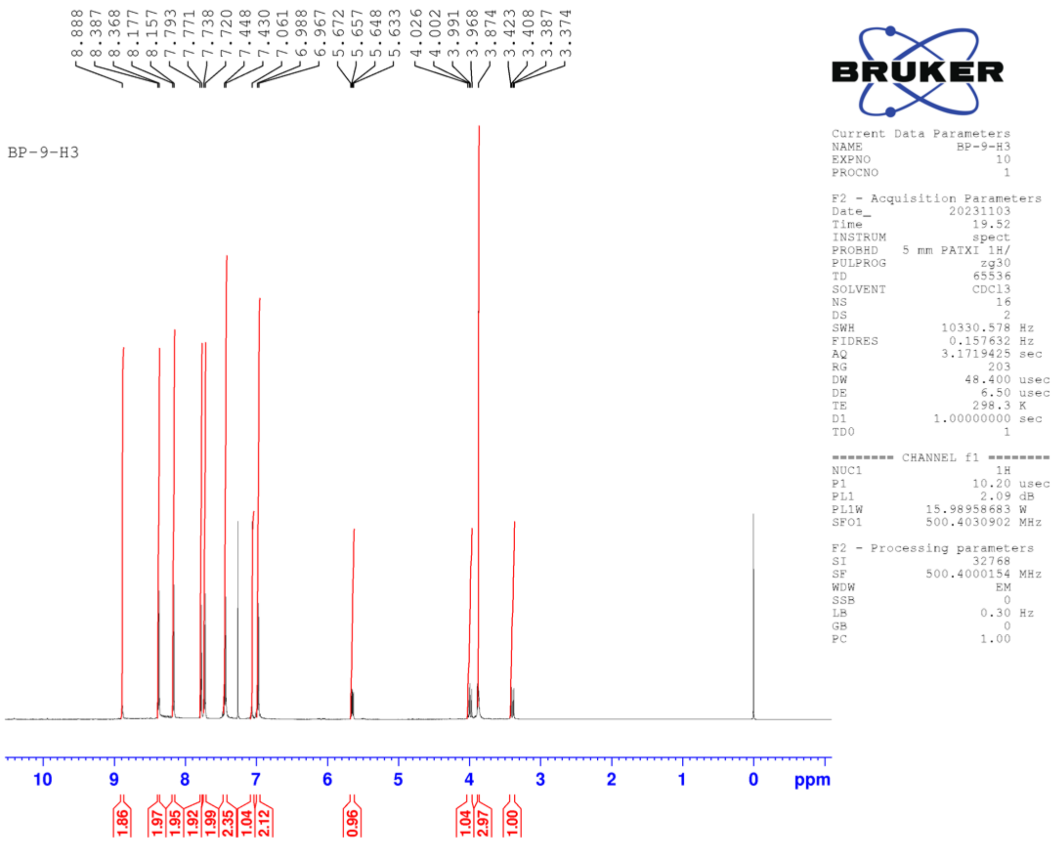

Figure S38: ^{13}C NMR Spectrum of **BP-9**

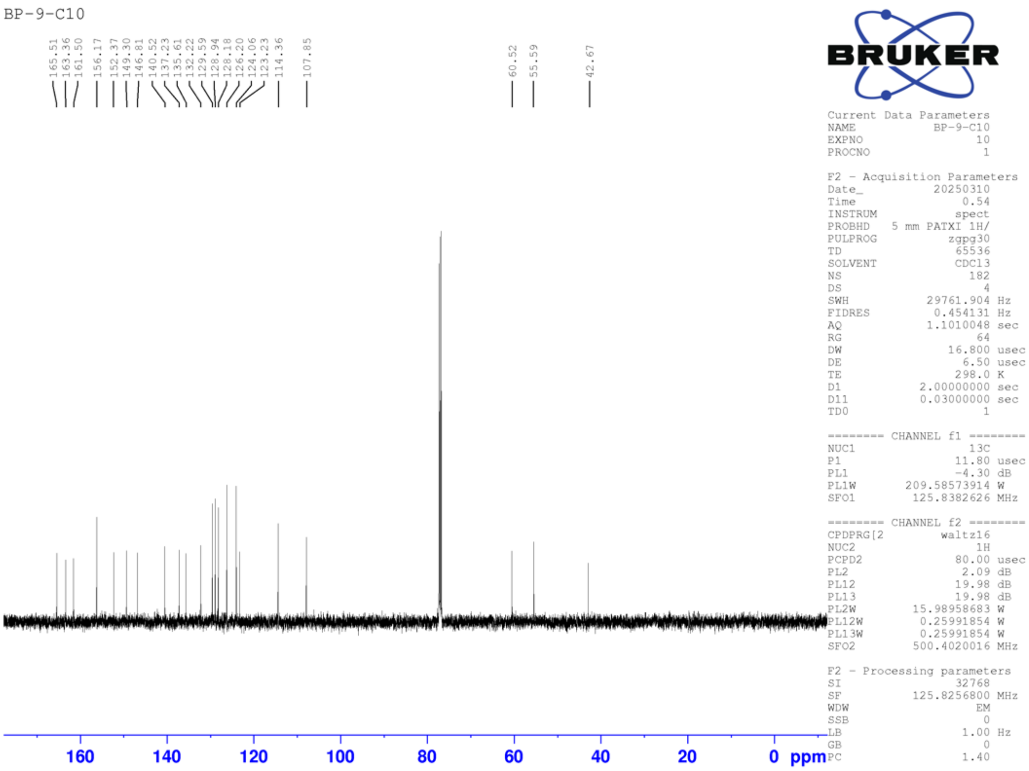

Figure S39: Mass Spectrum of **BP-9**

[Elemental Composition] Page: 1
Data : gousei768 Date : 08-Nov-2023 10:29
Sample: BP-9/sever belgin
Note : NBA
Inlet : Direct Ion Mode : FAB+
RT : 1.63 min Scan#: (4,11)
Elements : C 100/0, H 100/0, O 4/2, N 7/5, Cl 2/0, F 2/0, S 2/0
Mass Tolerance : 5ppm, 10mmu if m/z > 2000
Unsaturation (U.S.) : -0.5 - 100.0

Observed m/z	Int%	Err[ppm / mmu]	U.S.	Composition
568.1094	29.4	+1.7 / +1.0	32.0	C 35 H 13 O 2 N 6 F
		+4.7 / +2.7	18.5	C 26 H 21 O 3 N 7 Cl 2 F
		-0.3 / -0.2	28.0	C 32 H 14 O 3 N 6 F 2
		-1.2 / -0.7	23.5	C 29 H 17 O 2 N 7 Cl F 2
		-4.4 / -2.5	18.5	C 28 H 22 O 2 N 5 Cl 2 F 2
		+2.7 / +1.5	14.5	C 23 H 22 O 4 N 7 Cl 2 F 2
		+2.5 / +1.4	27.5	C 32 H 18 O 4 N 5 S
		+1.6 / +0.9	23.0	C 29 H 21 O 3 N 6 Cl S
		+0.8 / +0.4	18.5	C 26 H 24 O 2 N 7 Cl 2 S
		-4.2 / -2.4	28.0	C 32 H 17 O 2 N 6 F S
		-0.4 / -0.2	19.0	C 26 H 22 O 4 N 6 Cl F S
		-1.2 / -0.7	14.5	C 23 H 25 O 3 N 7 Cl 2 F S
		-3.2 / -1.8	10.5	C 20 H 26 O 4 N 7 Cl 2 F 2 S
		-3.4 / -1.9	23.5	C 29 H 22 O 4 N 5 S 2
		-4.3 / -2.4	19.0	C 26 H 25 O 3 N 6 Cl S 2
569.1144	30.0	+3.5 / +2.0	31.0	C 35 H 15 O 4 N 5
		+2.6 / +1.5	26.5	C 32 H 18 O 3 N 6 Cl
		+1.8 / +1.0	22.0	C 29 H 21 O 2 N 7 Cl 2
		-3.2 / -1.8	31.5	C 35 H 14 O 2 N 6 F
		+0.6 / +0.3	22.5	C 29 H 19 O 4 N 6 Cl F
		-0.2 / -0.1	18.0	C 26 H 22 O 3 N 7 Cl 2 F
		-2.3 / -1.3	14.0	C 23 H 23 O 4 N 7 Cl 2 F 2
		-2.5 / -1.4	27.0	C 32 H 19 O 4 N 5 S
		-3.3 / -1.9	22.5	C 29 H 22 O 3 N 6 Cl S
		-4.2 / -2.4	18.0	C 26 H 25 O 2 N 7 Cl 2 S
		+3.8 / +2.2	19.0	C 27 H 25 O 2 N 5 Cl F S 2
		+5.0 / +2.8	20.0	C 25 H 21 O 3 N 7 F 2 S 2
		+1.8 / +1.0	15.0	C 24 H 26 O 3 N 5 Cl F 2 S 2
		+0.9 / +0.5	10.5	C 21 H 29 O 2 N 6 Cl 2 F 2 S 2

[Theoretical Ion Distribution] Page: 1
Molecular Formula : C29 H22 O3 N6 Cl S
(m/z 569.1163, MW 570.0511, U.S. 22.5)
Base Peak : 569.1163, Averaged MW : 570.0509(a), 570.0531(w)

m/z	INT.	
569.1163	100.0000	**
570.1192	35.6925	*********************
571.1145	43.1936	**************************
572.1166	13.8673	********
573.1155	3.9906	**
574.1160	0.8380	
575.1170	0.1351	
576.1183	0.0176	
577.1199	0.0019	
578.1216	0.0002	

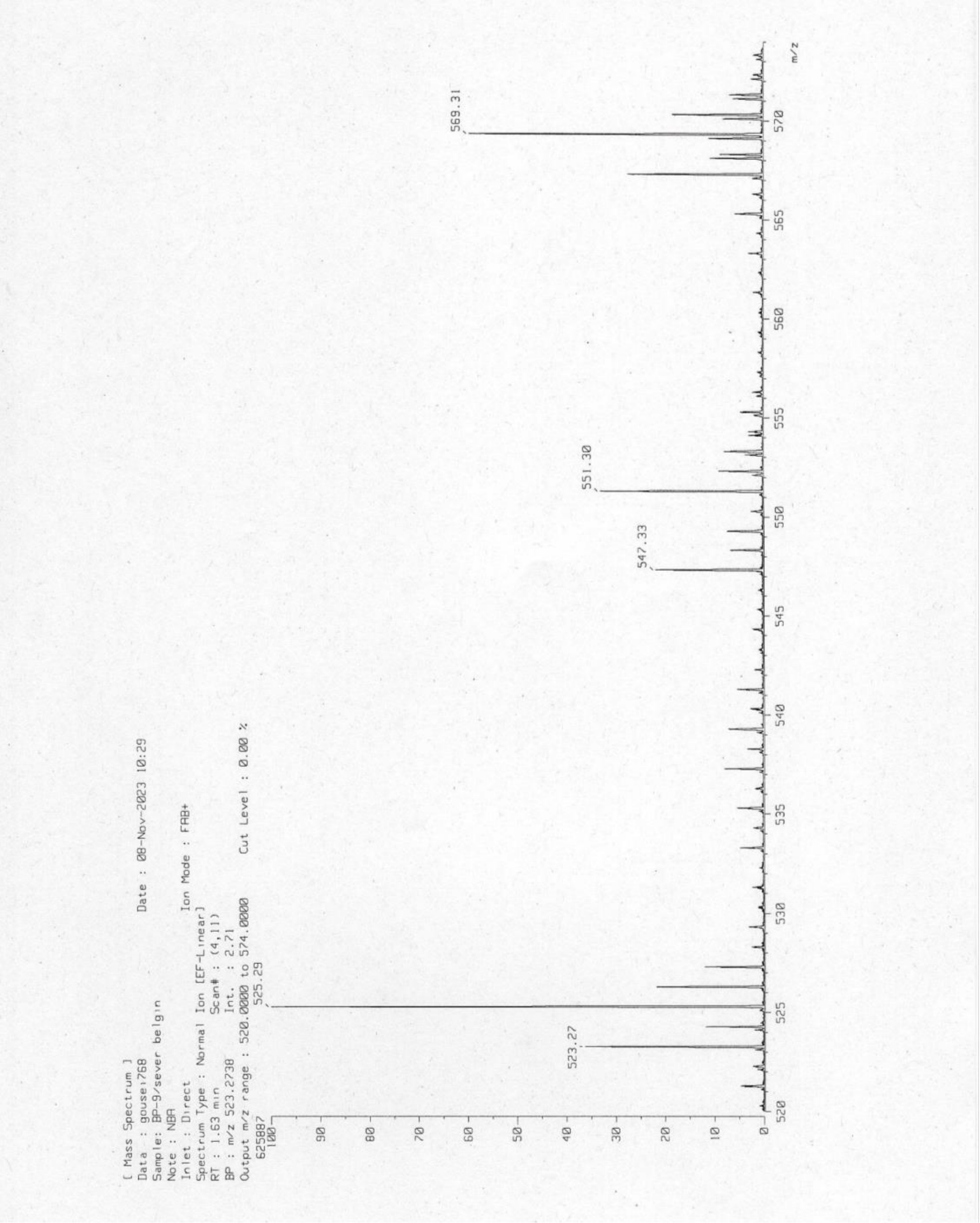
[Mass Spectrum]
Data : gouse1768
Date : 08-Nov-2023 10:29
Sample: BP-9/sever belgin
Note : NBA
Inlet : Direct
Ion Mode : FAB+
Spectrum Type : Normal Ion [EF-Linear]
RT : 1.63 min
Scan# : (4,11)
BP : m/z 523.2738
Int. : 2.71
Output m/z range : 520.0000 to 574.0000
Cut Level : 0.00 %
625887
100
90
80
70
60
50
40
30
20
10
0
523.27
525.29
547.33
551.30
569.31
520
525
530
535
540
545
550
555
560
565
570
m/z

Figure S40: ^{1}H NMR Spectrum of **BP-10**

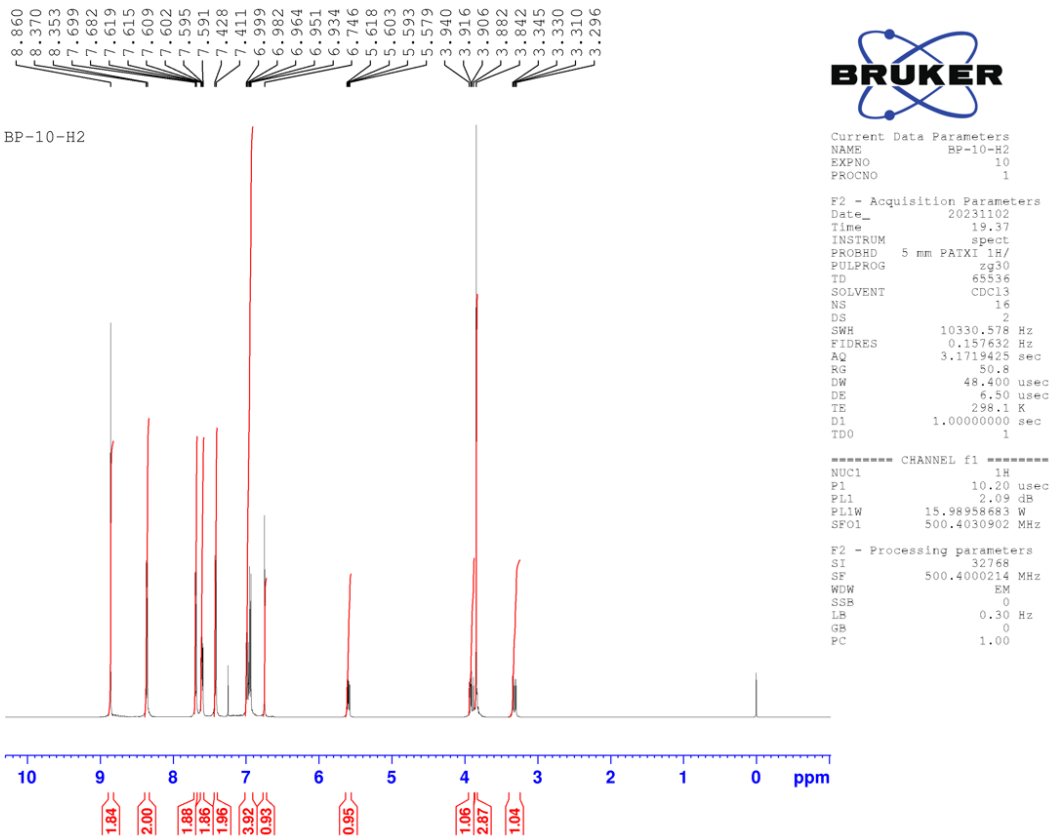

Figure S41: ^{13}C NMR Spectrum of **BP-10**

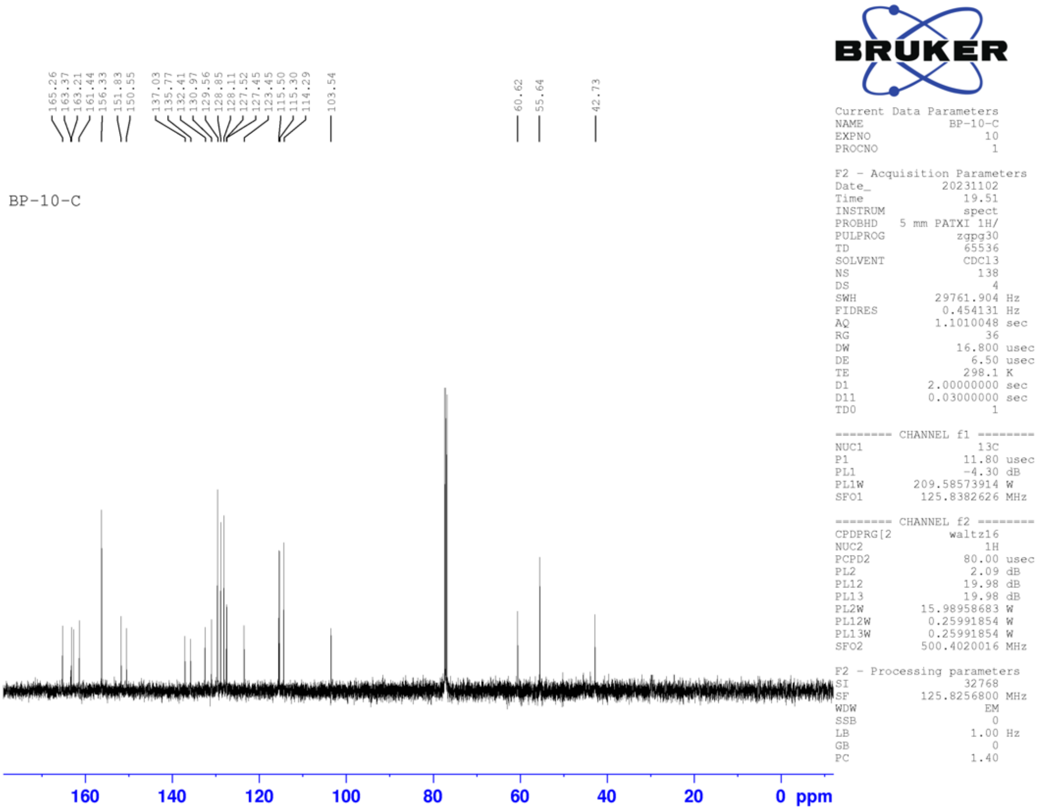

Figure S42: Mass Spectrum of **BP-10**

[Elemental Composition] Page: 1
Data : gousei767 Date : 02-Nov-2023 14:56
Sample: BP-10/sever belgin
Note : NBA
Inlet : Direct Ion Mode : FAB+
RT : 1.50 min Scan#: (4,10)
Elements : C 100/0, H 100/0, O 2/0, N 6/4, Cl 2/0, F 2/0, S 2/0
Mass Tolerance : 20ppm, 10mmu if m/z < 500, 20mmu if m/z > 1000
Unsaturation (U.S.) : -0.5 - 100.0

Observed m/z	Int%	Err[ppm / mmu]	U.S.	Composition
541.1155	100.0	+12.2 / +6.6	34.5	C 38 H 13 O N 4
		-8.6 / -4.6	34.5	C 37 H 13 N 6
		-11.9 / -6.5	29.5	C 36 H 18 N 4 Cl
		+11.3 / +6.1	30.0	C 35 H 16 N 5 Cl
		-4.5 / -2.4	25.5	C 31 H 18 O 2 N 6 Cl
		-7.9 / -4.3	20.5	C 30 H 23 O 2 N 4 Cl 2
		+15.4 / +8.3	21.0	C 29 H 21 O 2 N 5 Cl 2
		+10.1 / +5.5	30.5	C 35 H 14 O 2 N 4 F
		-10.7 / -5.8	30.5	C 34 H 14 O N 6 F
		-14.1 / -7.6	25.5	C 33 H 19 O N 4 Cl F
		+9.2 / +5.0	26.0	C 32 H 17 O N 5 Cl F
		-15.0 / -8.1	21.0	C 30 H 22 N 5 Cl 2 F
		+8.3 / +4.5	21.5	C 29 H 20 N 6 Cl 2 F
		+3.0 / +1.6	31.0	C 35 H 13 N 5 F 2
		-12.8 / -6.9	26.5	C 31 H 15 O 2 N 6 F 2
		-16.2 / -8.7	21.5	C 30 H 20 O 2 N 4 Cl F 2
		+7.1 / +3.8	22.0	C 29 H 18 O 2 N 5 Cl F 2
		-17.1 / -9.2	17.0	C 27 H 23 O N 5 Cl 2 F 2
		+6.2 / +3.3	17.5	C 26 H 21 O N 6 Cl 2 F 2
		+6.0 / +3.2	30.5	C 35 H 17 O N 4 S
		-14.8 / -8.0	30.5	C 34 H 17 N 6 S
		-18.2 / -9.8	25.5	C 33 H 22 N 4 Cl S
		+5.1 / +2.7	26.0	C 32 H 20 N 5 Cl S
		-10.7 / -5.8	21.5	C 28 H 22 O 2 N 6 Cl S
		-14.1 / -7.6	16.5	C 27 H 27 O 2 N 4 Cl 2 S
		+9.1 / +4.9	17.0	C 26 H 25 O 2 N 5 Cl 2 S
		+3.9 / +2.1	26.5	C 32 H 18 O 2 N 4 F S
		-16.9 / -9.1	26.5	C 31 H 18 O N 6 F S
		+3.0 / +1.6	22.0	C 29 H 21 O N 5 Cl F S
		+2.1 / +1.1	17.5	C 26 H 24 N 6 Cl 2 F S
		-3.2 / -1.7	27.0	C 32 H 17 N 5 F 2 S
		-19.0 / -10.3	22.5	C 28 H 19 O 2 N 6 F 2 S
		+16.7 / +9.0	22.5	C 30 H 20 N 4 Cl F 2 S
		+0.8 / +0.5	18.0	C 26 H 22 O 2 N 5 Cl F 2 S
		-0.1 / +0.0	13.5	C 23 H 25 O N 6 Cl 2 F 2 S
		-0.3 / -0.1	26.5	C 32 H 21 O N 4 S 2
		-1.2 / -0.6	22.0	C 29 H 24 N 5 Cl S 2
		-17.0 / -9.2	17.5	C 25 H 26 O 2 N 6 Cl S 2
		+18.7 / +10.1	17.5	C 27 H 27 N 4 Cl 2 S 2
		+2.9 / +1.6	13.0	C 23 H 29 O 2 N 5 Cl 2 S 2
		-2.4 / -1.3	22.5	C 29 H 22 O 2 N 4 F S 2
		-3.3 / -1.8	18.0	C 26 H 25 O N 5 Cl F S 2
		+20.0 / +10.8	18.5	C 25 H 23 O N 6 Cl F S 2
		+16.6 / +9.0	13.5	C 24 H 28 O N 4 Cl 2 F S 2
		-4.2 / -2.3	13.5	C 23 H 28 N 6 Cl 2 F S 2
		-9.4 / -5.1	23.0	C 29 H 21 N 5 F 2 S 2
		+13.8 / +7.5	23.5	C 28 H 19 N 6 F 2 S 2
		+10.4 / +5.6	18.5	C 27 H 24 N 4 Cl F 2 S 2
		-5.4 / -2.9	14.0	C 23 H 26 O 2 N 5 Cl F 2 S 2
		+17.9 / +9.7	14.5	C 22 H 24 O 2 N 6 Cl F 2 S 2
		+14.5 / +7.8	9.5	C 21 H 29 O 2 N 4 Cl 2 F 2 S 2
		-6.3 / -3.4	9.5	C 20 H 29 O N 6 Cl 2 F 2 S 2
542.1211	95.7	+8.0 / +4.3	34.0	C 38 H 14 O N 4
		-12.7 / -6.9	34.0	C 37 H 14 N 6

[Elemental Composition]   Page: 2

-16.1 /	-8.7	29.0	C 36 H 19 N 4 Cl
+7.1 /	+3.8	29.5	C 35 H 17 N 5 Cl
-8.7 /	-4.7	25.0	C 31 H 19 O 2 N 6 Cl
-12.1 /	-6.5	20.0	C 30 H 24 O 2 N 4 Cl 2
+11.1 /	+6.0	20.5	C 29 H 22 O 2 N 5 Cl 2
+5.9 /	+3.2	30.0	C 35 H 15 O 2 N 4 F
-14.8 /	-8.0	30.0	C 34 H 15 O N 6 F
-18.2 /	-9.9	25.0	C 33 H 20 O N 4 Cl F
+5.0 /	+2.7	25.5	C 32 H 18 O N 5 Cl F
-19.1 /	-10.4	20.5	C 30 H 23 N 5 Cl 2 F
+4.1 /	+2.2	21.0	C 29 H 21 N 6 Cl 2 F
-1.2 /	-0.6	30.5	C 35 H 14 N 5 F 2
-16.9 /	-9.2	26.0	C 31 H 16 O 2 N 6 F 2
+18.7 /	+10.1	26.0	C 33 H 17 N 4 Cl F 2
+2.9 /	+1.6	21.5	C 29 H 19 O 2 N 5 Cl F 2
+2.0 /	+1.1	17.0	C 26 H 22 O N 6 Cl 2 F 2
+1.8 /	+1.0	30.0	C 35 H 18 O N 4 S
-18.9 /	-10.3	30.0	C 34 H 18 N 6 S
+0.9 /	+0.5	25.5	C 32 H 21 N 5 Cl S
-14.9 /	-8.1	21.0	C 28 H 23 O 2 N 6 Cl S
-18.3 /	-9.9	16.0	C 27 H 28 O 2 N 4 Cl 2 S
+4.9 /	+2.7	16.5	C 26 H 26 O 2 N 5 Cl 2 S
-0.3 /	-0.2	26.0	C 32 H 19 O 2 N 4 F S
-1.2 /	-0.7	21.5	C 29 H 22 O N 5 Cl F S
+18.6 /	+10.1	17.0	C 27 H 25 O N 4 Cl 2 F S
-2.1 /	-1.2	17.0	C 26 H 25 N 6 Cl 2 F S
-7.4 /	-4.0	26.5	C 32 H 18 N 5 F 2 S
+15.8 /	+8.6	27.0	C 31 H 16 N 6 F 2 S
+12.4 /	+6.7	22.0	C 30 H 21 N 4 Cl F 2 S
-3.3 /	-1.8	17.5	C 26 H 23 O 2 N 5 Cl F 2 S
+19.9 /	+10.8	18.0	C 25 H 21 O 2 N 6 Cl F 2 S
+16.5 /	+8.9	13.0	C 24 H 26 O 2 N 4 Cl 2 F 2 S
-4.2 /	-2.3	13.0	C 23 H 26 O N 6 Cl 2 F 2 S
-4.4 /	-2.4	26.0	C 32 H 22 O N 4 S 2
+18.7 /	+10.2	26.5	C 31 H 20 O N 5 S 2
-5.3 /	-2.9	21.5	C 29 H 25 N 5 Cl S 2
+17.9 /	+9.7	22.0	C 28 H 23 N 6 Cl S 2
+14.5 /	+7.8	17.0	C 27 H 28 N 4 Cl 2 S 2
-1.3 /	-0.7	12.5	C 23 H 30 O 2 N 5 Cl 2 S 2
-6.6 /	-3.6	22.0	C 29 H 23 O 2 N 4 F S 2
+16.6 /	+9.0	22.5	C 28 H 21 O 2 N 5 F S 2
-7.5 /	-4.0	17.5	C 26 H 26 O N 5 Cl F S 2
+15.7 /	+8.5	18.0	C 25 H 24 O N 6 Cl F S 2
+12.4 /	+6.7	13.0	C 24 H 29 O N 4 Cl 2 F S 2
-8.4 /	-4.5	13.0	C 23 H 29 N 6 Cl 2 F S 2
-13.6 /	-7.4	22.5	C 29 H 22 N 5 F 2 S 2
+9.6 /	+5.2	23.0	C 28 H 20 N 6 F 2 S 2
+6.2 /	+3.4	18.0	C 27 H 25 N 4 Cl F 2 S 2
-9.6 /	-5.2	13.5	C 23 H 27 O 2 N 5 Cl F 2 S 2
+13.6 /	+7.4	14.0	C 22 H 25 O 2 N 6 Cl F 2 S 2
+10.3 /	+5.6	9.0	C 21 H 30 O 2 N 4 Cl 2 F 2 S 2
-10.5 /	-5.7	9.0	C 20 H 30 O N 6 Cl 2 F 2 S 2

[Theoretical Ion Distribution]   Page: 1

Molecular Formula : C29 H21 O N5 Cl F S
(m/z 541.1139, MW 542.0360, U.S. 22.0)
Base Peak : 541.1139, Averaged MW : 542.0360(a), 542.0383(w)

m/z	INT.	
541.1139	100.0000	***
542.1169	35.2340	*******************
543.1121	42.6307	***********************
544.1143	13.5288	********
545.1131	3.7565	**
546.1133	0.7656	
547.1144	0.1161	
548.1158	0.0139	
549.1173	0.0014	
550.1191	0.0001	

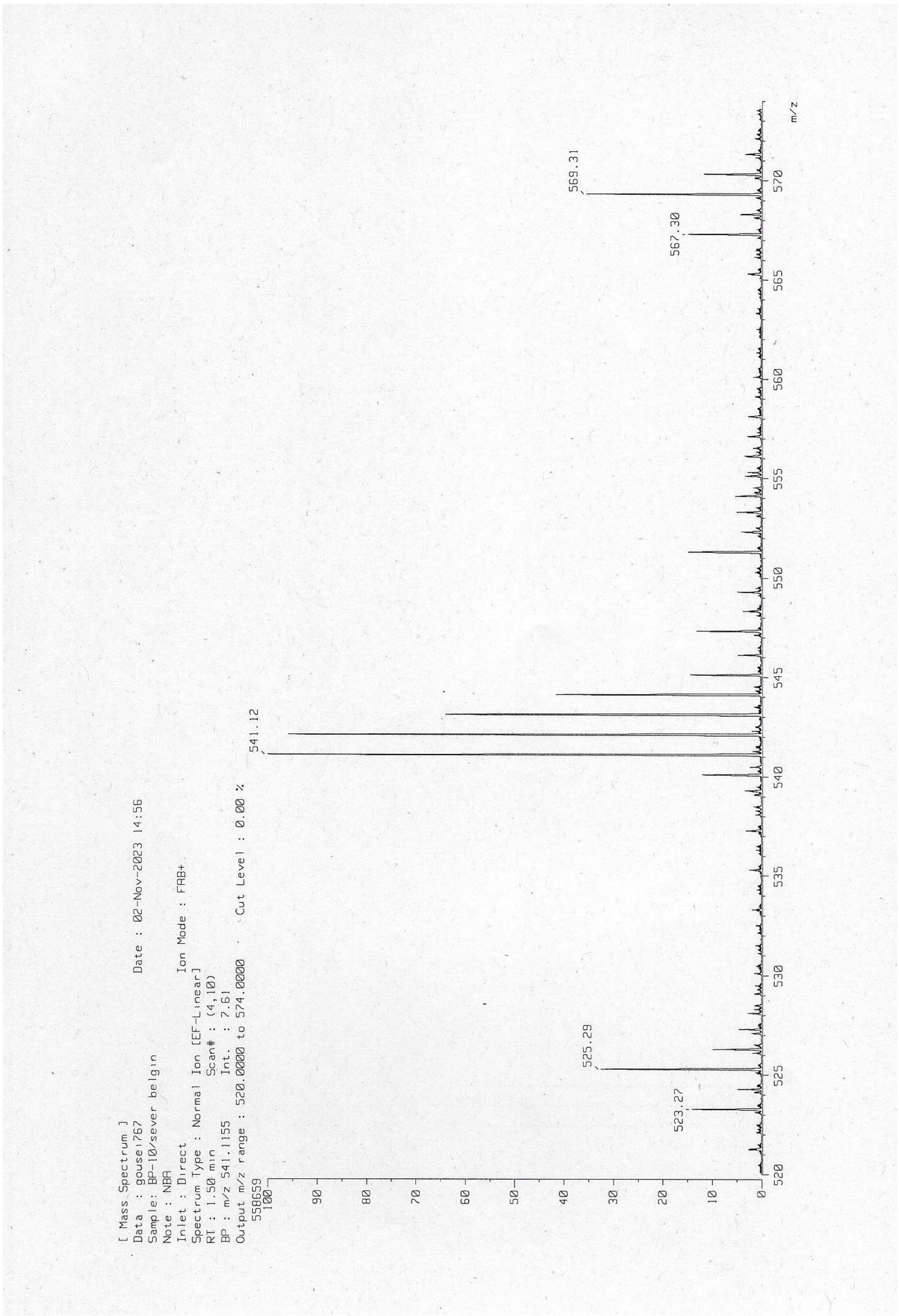
[Mass Spectrum]
Data : gouse1767
Date : 02-Nov-2023 14:56
Sample: BP-10/sever belgin
Note : NBA
Inlet : Direct
Ion Mode : FAB+
Spectrum Type : Normal Ion [EF-Linear]
RT : 1.50 min
Scan# : (4,10)
BP : m/z 541.1155
Int. : 2.61
Output m/z range : 520.0000 to 574.0000
Cut Level : 0.00 %
558659
523.27
525.29
541.12
567.30
569.31
m/z

Figure S43: ^{1}H NMR Spectrum of **BP-11**

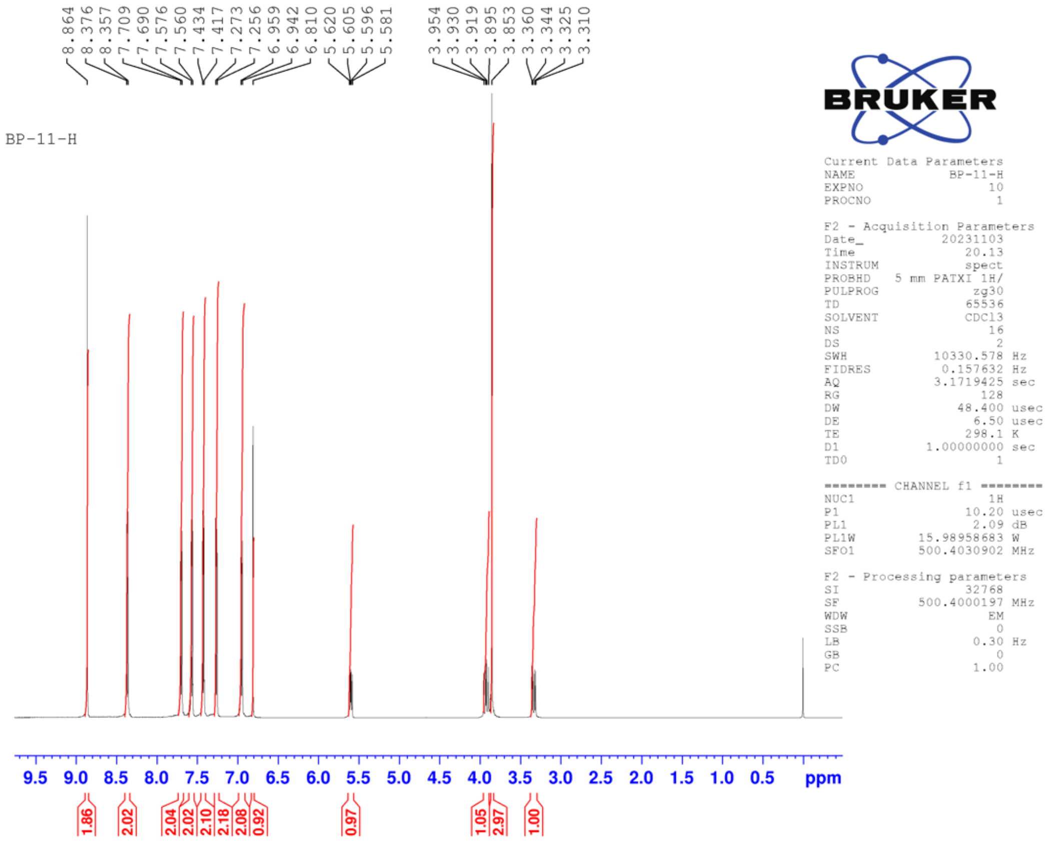

Figure S44: ^{13}C NMR Spectrum of **BP-11**

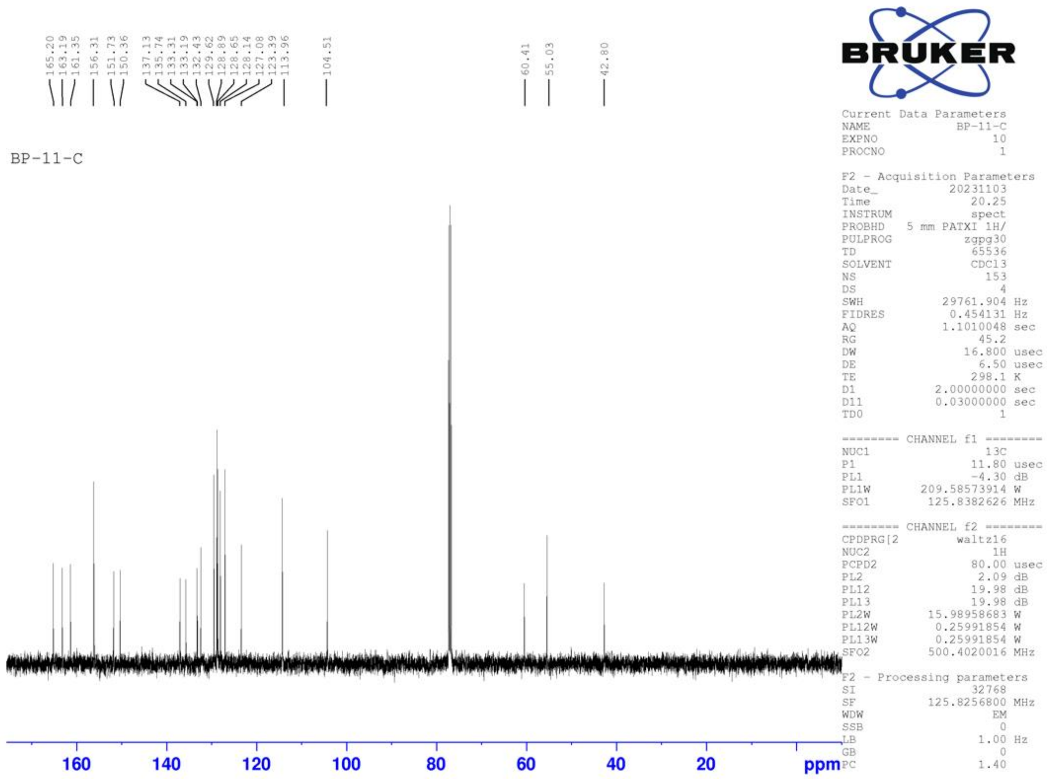

Figure S45: Mass Spectrum of **BP-11**

```
[ Elemental Composition ]                                              Page: 1
Data   : gousei769                         Date : 08-Nov-2023 10:45
Sample: BP-11/sever belgin
Note : NBA
Inlet : Direct                             Ion Mode : FAB+
RT : 1.88 min                              Scan#: (6,11)
Elements : C 100/0, H 100/0, O 2/0, N 6/4, Cl 3/1, S 2/0
Mass Tolerance      : 5ppm, 10mmu if m/z > 2000
Unsaturation (U.S.) : -0.5 - 100.0

Observed m/z Int%    Err[ppm / mmu]     U.S.  Composition
  557.0859    93.8      +3.7 /  +2.0    26.5  C 32 H 18 O 2 N 4 Cl S
                        +2.8 /  +1.5    22.0  C 29 H 21 O N 5 Cl 2 S
                        +1.9 /  +1.1    17.5  C 26 H 24 N 6 Cl 3 S
                        -2.4 /  -1.3    22.5  C 29 H 22 O 2 N 4 Cl S 2
                        -3.3 /  -1.8    18.0  C 26 H 25 O N 5 Cl 2 S 2
                        -4.1 /  -2.3    13.5  C 23 H 28 N 6 Cl 3 S 2

  558.0909   100.0      +4.6 /  +2.6    30.0  C 35 H 15 O 2 N 4 Cl
                        +3.8 /  +2.1    25.5  C 32 H 18 O N 5 Cl 2
                        +2.9 /  +1.6    21.0  C 29 H 21 N 6 Cl 3
                        -1.4 /  -0.8    26.0  C 32 H 19 O 2 N 4 Cl S
                        -2.3 /  -1.3    21.5  C 29 H 22 O N 5 Cl 2 S
                        -3.1 /  -1.8    17.0  C 26 H 25 N 6 Cl 3 S
```

```
[ Theoretical Ion Distribution ]                                       Page: 1
Molecular Formula : C29 H22 O N5 Cl2 S
                      (m/z   558.0922, MW   559.4986, U.S. 21.5)
Base Peak :    558.0922, Averaged MW :    559.4983(a),   559.5019(w)

    m/z        INT.
  558.0922 100.0000 ************************************************************
  559.0952  35.2490 *********************
  560.0899  74.6144 *********************************************
  561.0924  24.8072 ***************
  562.0883  17.3928 **********
  563.0899   5.0945 ***
  564.0888   1.3181 *
  565.0890   0.2589
  566.0899   0.0385
  567.0912   0.0046
  568.0928   0.0004
```

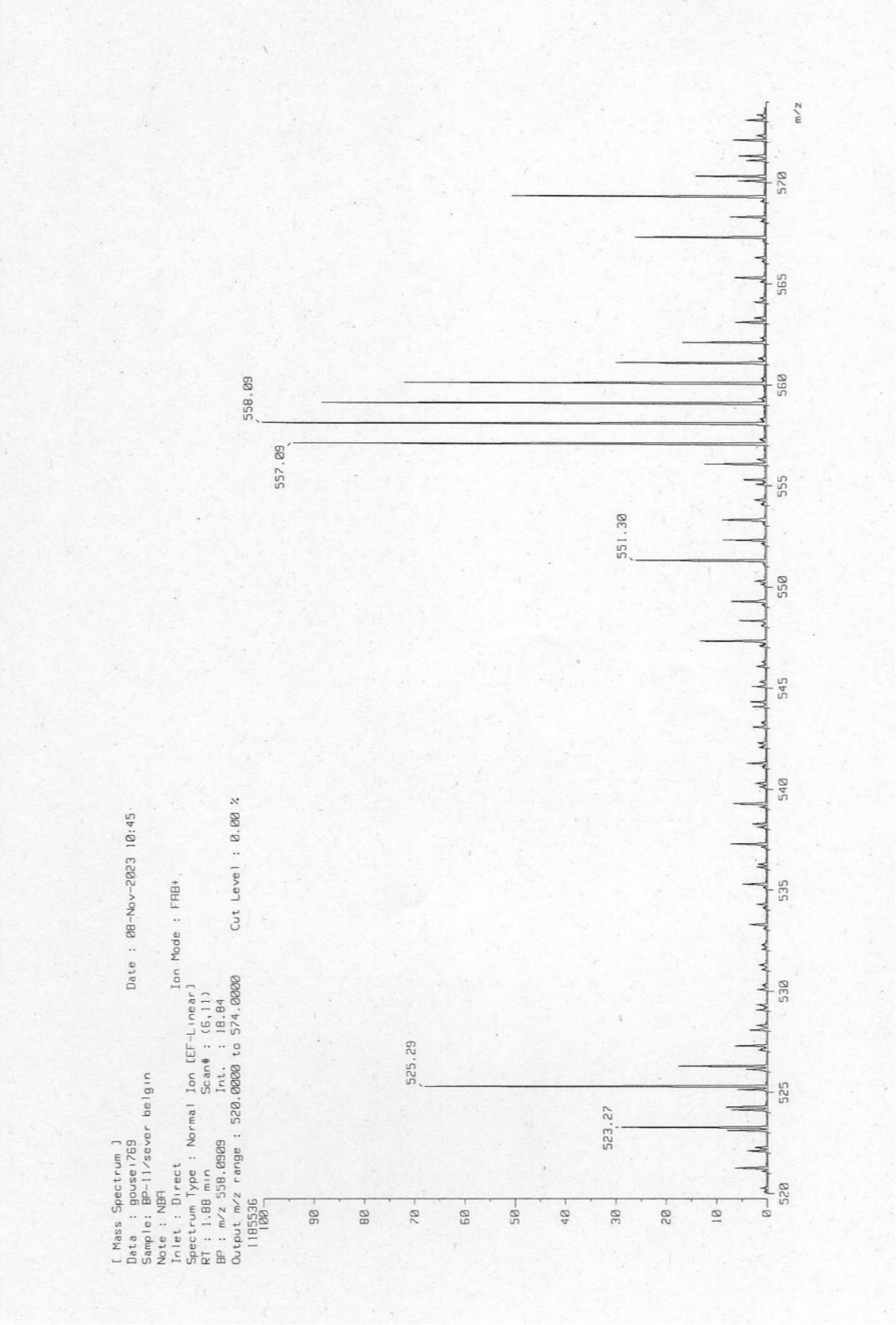
[Mass Spectrum]
Data : gouse1769
Sample: BP-11/sever belgin
Note : NBA
Inlet : Direct
Ion Mode : FAB+
Spectrum Type : Normal Ion [EF-Linear]
RT : 1.88 min
Scan# : (6,11)
BP : m/z 558.0909
Int. : 18.84
Output m/z range : 520.0000 to 574.0000
Cut Level : 0.00 %
Date : 08-Nov-2023 10:45
1185536
523.27
525.29
551.30
557.09
558.09
m/z

Figure S46: ^{1}H NMR Spectrum of **BP-12**

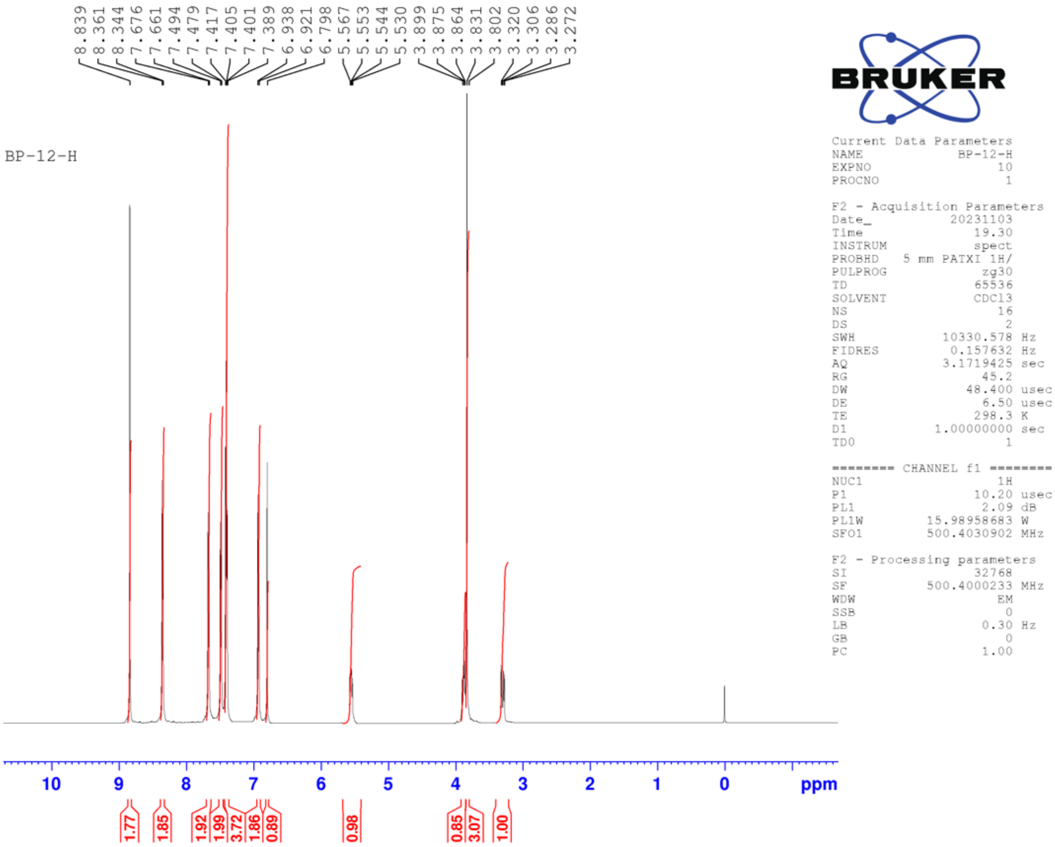

Figure S47: ^{13}C NMR Spectrum of **BP-12**

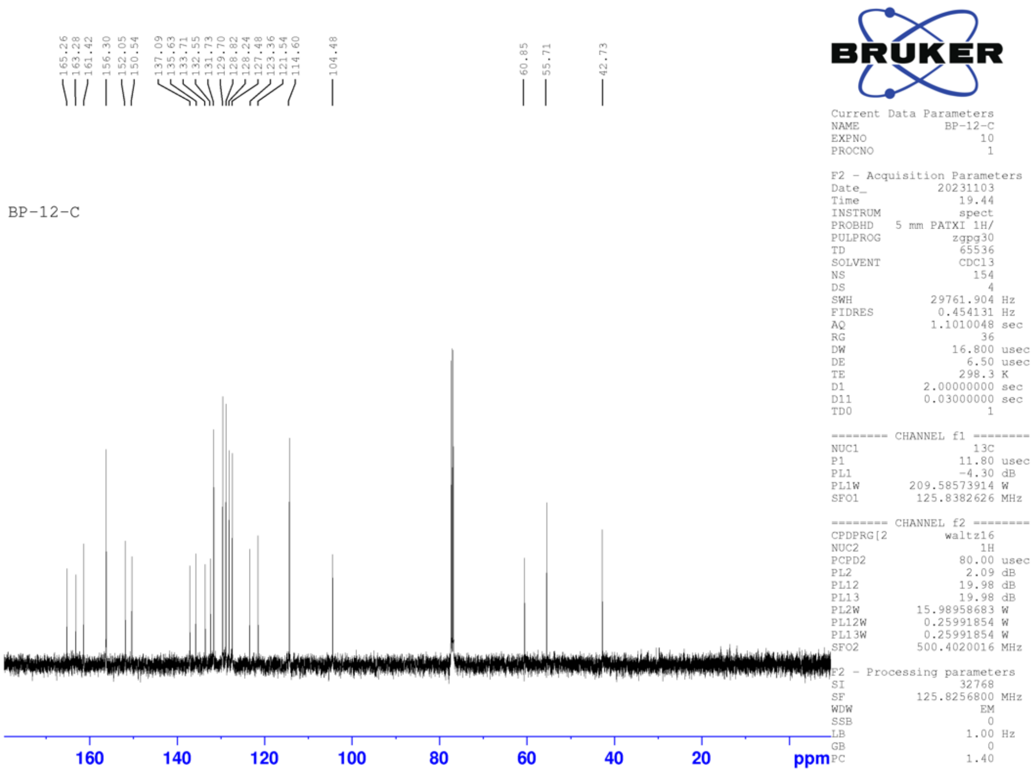

Figure S48: Mass Spectrum of **BP-12**

[Elemental Composition] Page: 1
Data : gousei770 Date : 08-Nov-2023 11:01
Sample: BP-12/sever belgin
Note : NBA
Inlet : Direct Ion Mode : FAB+
RT : 1.13 min Scan#: (3,8)
Elements : C 100/0, H 100/0, O 2/0, N 6/4, Cl 2/0, Br 2/0, S 2/0
Mass Tolerance : 5ppm, 10mmu if m/z > 2000
Unsaturation (U.S.) : -0.5 - 100.0

Observed m/z	Int%	Err[ppm / mmu]	U.S.	Composition
601.0350	64.8	-2.7 / -1.6	37.0	C 38 H 8 O 2 N 5 Cl
		-3.5 / -2.1	32.5	C 35 H 11 O N 6 Cl 2
		-0.1 / -0.1	19.5	C 28 H 23 N 6 Br 2
		-3.1 / -1.9	14.5	C 27 H 28 N 4 Cl Br 2
		+3.6 / +2.1	10.5	C 22 H 28 O 2 N 6 Cl Br 2
		+0.5 / +0.3	5.5	C 21 H 33 O 2 N 4 Cl 2 Br 2
		+2.7 / +1.7	26.5	C 32 H 18 O 2 N 4 Br S
		+1.9 / +1.2	22.0	C 29 H 21 O N 5 Cl Br S
		+1.1 / +0.7	17.5	C 26 H 24 N 6 Cl 2 Br S
		-2.1 / -1.2	6.5	C 19 H 32 O 2 N 6 Cl Br 2 S
		+3.4 / +2.0	38.5	C 37 H 9 N 6 S 2
		+0.3 / +0.2	33.5	C 36 H 14 N 4 Cl S 2
		+4.0 / +2.4	24.5	C 30 H 19 O 2 N 4 Cl 2 S 2
		-2.9 / -1.7	22.5	C 29 H 22 O 2 N 4 Br S 2
		-3.7 / -2.2	18.0	C 26 H 25 O N 5 Cl Br S 2
		-4.5 / -2.7	13.5	C 23 H 28 N 6 Cl 2 Br S 2
602.0392	74.6	+2.3 / +1.4	30.0	C 35 H 15 O 2 N 4 Br
		+1.5 / +0.9	25.5	C 32 H 18 O N 5 Cl Br
		+0.7 / +0.4	21.0	C 29 H 21 N 6 Cl 2 Br
		-2.5 / -1.5	10.0	C 22 H 29 O 2 N 6 Cl Br 2
		+3.0 / +1.8	42.0	C 40 H 6 N 6 S
		-0.1 / -0.1	37.0	C 39 H 11 N 4 Cl S
		+3.6 / +2.1	28.0	C 33 H 16 O 2 N 4 Cl 2 S
		-3.3 / -2.0	26.0	C 32 H 19 O 2 N 4 Br S
		-4.1 / -2.5	21.5	C 29 H 22 O N 5 Cl Br S
		-4.9 / -2.9	17.0	C 26 H 25 N 6 Cl 2 Br S
		-2.6 / -1.6	38.0	C 37 H 10 N 6 S 2
		+1.0 / +0.6	29.0	C 31 H 15 O 2 N 6 Cl S 2
		-2.0 / -1.2	24.0	C 30 H 20 O 2 N 4 Cl 2 S 2
		+1.4 / +0.8	11.0	C 23 H 32 O N 4 Br 2 S 2
		+0.5 / +0.3	6.5	C 20 H 35 N 5 Cl Br 2 S 2

[Theoretical Ion Distribution] Page: 1
Molecular Formula : C29 H22 O N5 Cl Br S
(m/z 602.0417, MW 603.9496, U.S. 21.5)
Base Peak : 604.0397, Averaged MW : 603.9491(a), 603.9528(w)

m/z	INT.	
602.0417	71.4727	***
603.0447	25.1934	****************
604.0397	100.0000	***
605.0425	34.1815	*********************
606.0381	32.3298	********************
607.0401	9.9582	******
608.0389	2.6962	**
609.0391	0.5426	
610.0402	0.0818	
611.0415	0.0098	
612.0431	0.0010	

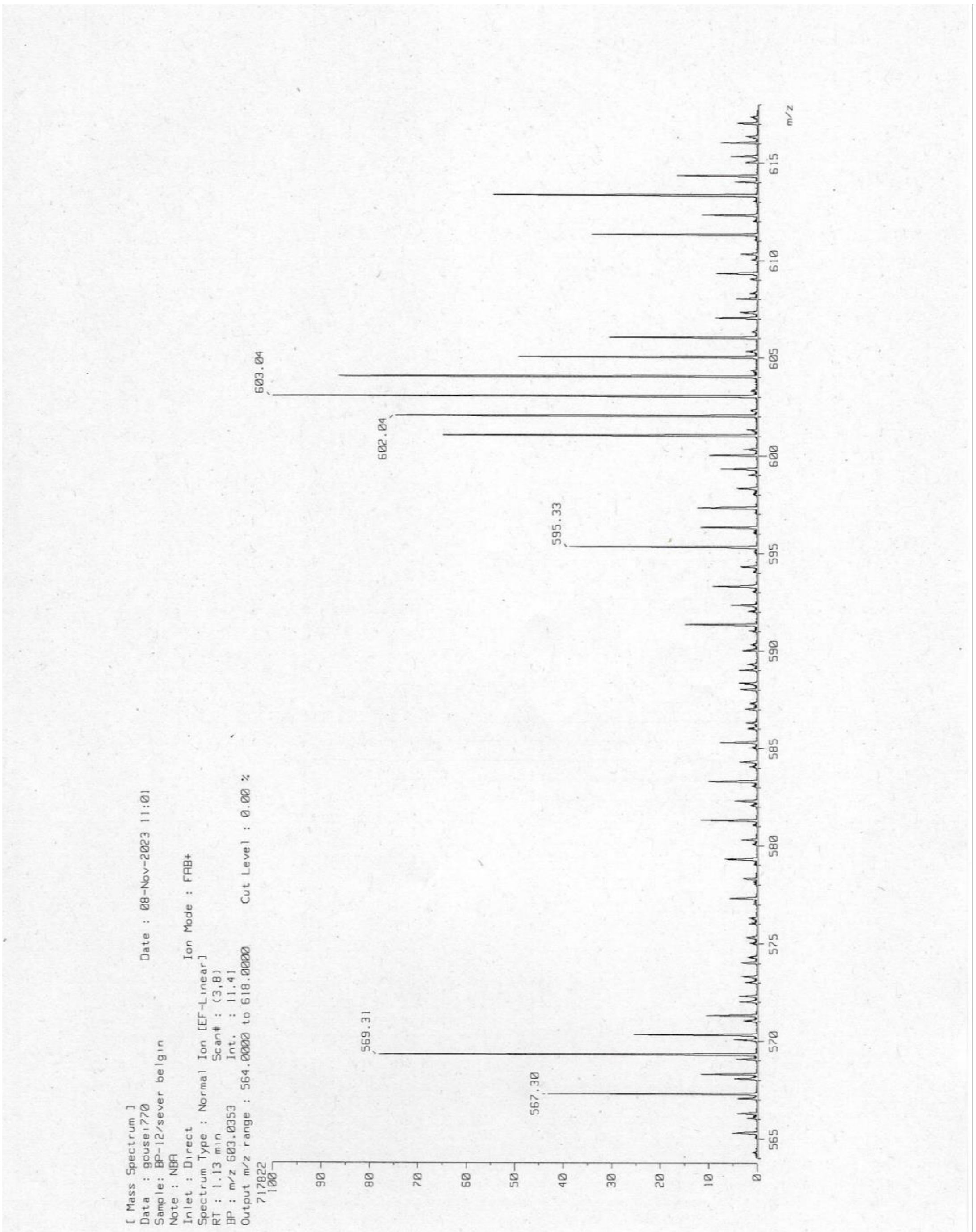
[Mass Spectrum]
Data : gouse1770
Date : 08-Nov-2023 11:01
Sample: BP-12/sever belgin
Note : NBA
Inlet : Direct
Ion Mode : FAB+
Spectrum Type : Normal Ion [EF-Linear]
RT : 1.13 min
Scan# : (3,8)
BP : m/z 603.0353
Int. : 11.41
Output m/z range : 564.0000 to 618.0000
Cut Level : 0.00 %
717822
567.30
569.31
595.33
602.04
603.04
m/z

Figure S49: ^{1}H NMR Spectrum of **BP-13**

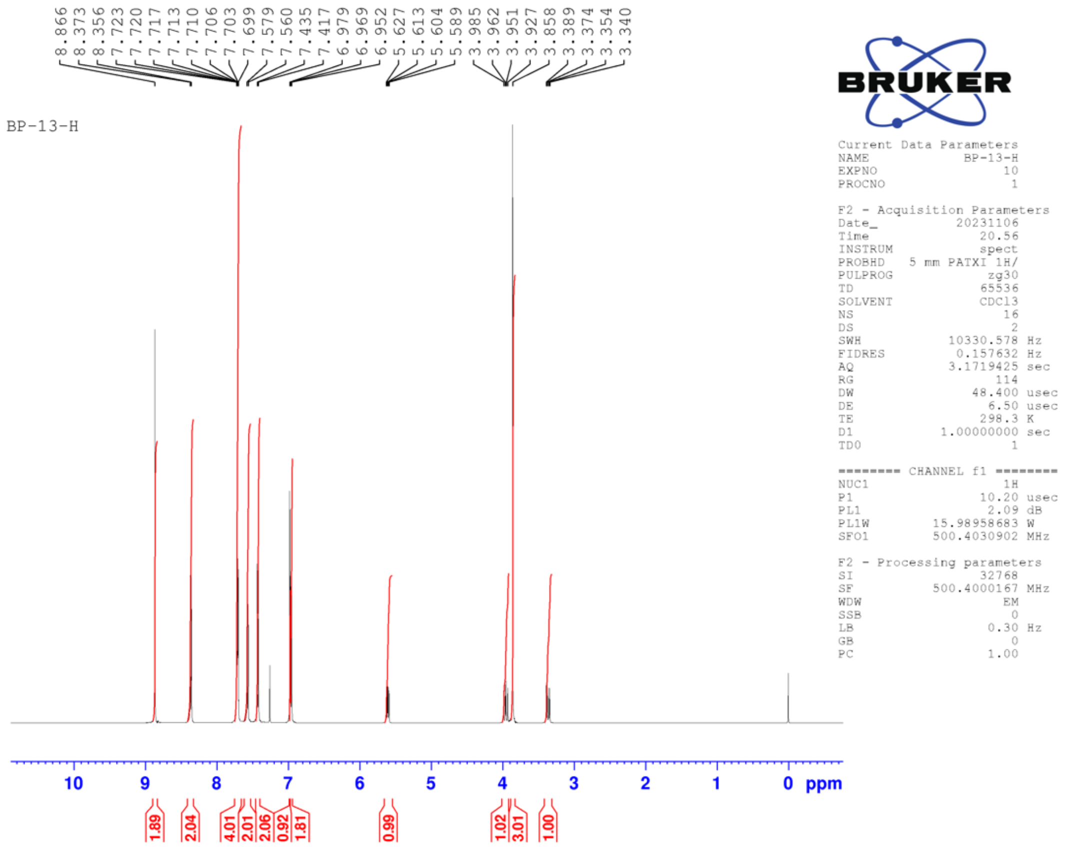

Figure S50: ^{13}C NMR Spectrum of **BP-13**

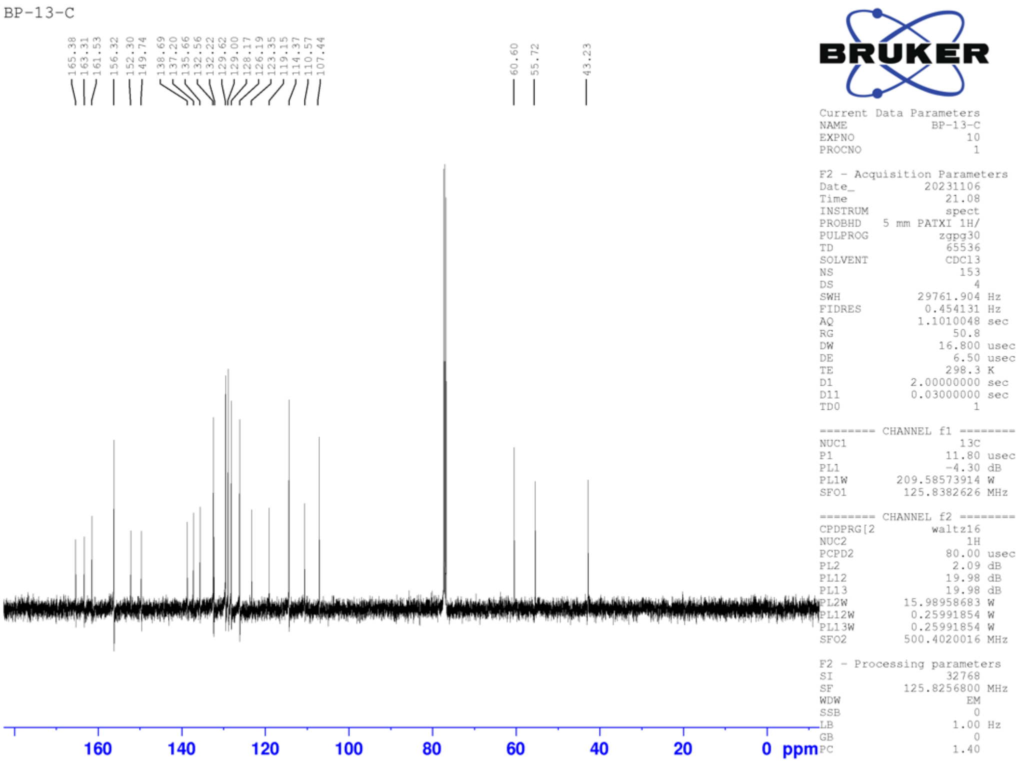

Figure S51: Mass Spectrum of **BP-13**

[Elemental Composition] Page: 1

Data : gousei771 Date : 08-Nov-2023 11:24
Sample: BP-13/sever belgin
Note : NBA
Inlet : Direct Ion Mode : FAB+
RT : 1.13 min Scan#: (3,8)
Elements : C 100/0, H 100/0, O 2/0, N 7/5, Cl 2/0, S 2/0
Mass Tolerance : 5ppm, 10mmu if m/z > 2000
Unsaturation (U.S.) : -0.5 - 100.0

Observed m/z	Int%	Err[ppm / mmu]	U.S.	Composition
548.1200	100.0	+3.4 / +1.9	28.5	C 33 H 18 O 2 N 5 S
		+2.5 / +1.4	24.0	C 30 H 21 O N 6 Cl S
		+1.6 / +0.9	19.5	C 27 H 24 N 7 Cl 2 S
		-2.7 / -1.5	24.5	C 30 H 22 O 2 N 5 S 2
		-3.6 / -2.0	20.0	C 27 H 25 O N 6 Cl S 2
		-4.5 / -2.5	15.5	C 24 H 28 N 7 Cl 2 S 2
549.1252	94.9	+4.7 / +2.6	32.0	C 36 H 15 O 2 N 5
		+3.9 / +2.1	27.5	C 33 H 18 O N 6 Cl
		+3.0 / +1.6	23.0	C 30 H 21 N 7 Cl 2
		-1.4 / -0.8	28.0	C 33 H 19 O 2 N 5 S
		-2.3 / -1.3	23.5	C 30 H 22 O N 6 Cl S
		-3.2 / -1.7	19.0	C 27 H 25 N 7 Cl 2 S

[Theoretical Ion Distribution] Page: 1

Molecular Formula : C30 H22 O N6 Cl S
(m/z 549.1264, MW 550.0633, U.S. 23.5)
Base Peak : 549.1264, Averaged MW : 550.0633(a), 550.0656(w)

m/z	INT.	
549.1264	100.0000	***
550.1294	36.7286	*********************
551.1247	43.1617	*************************
552.1268	14.1675	********
553.1257	3.9605	**
554.1259	0.8223	
555.1270	0.1277	
556.1283	0.0157	
557.1298	0.0016	
558.1316	0.0001	

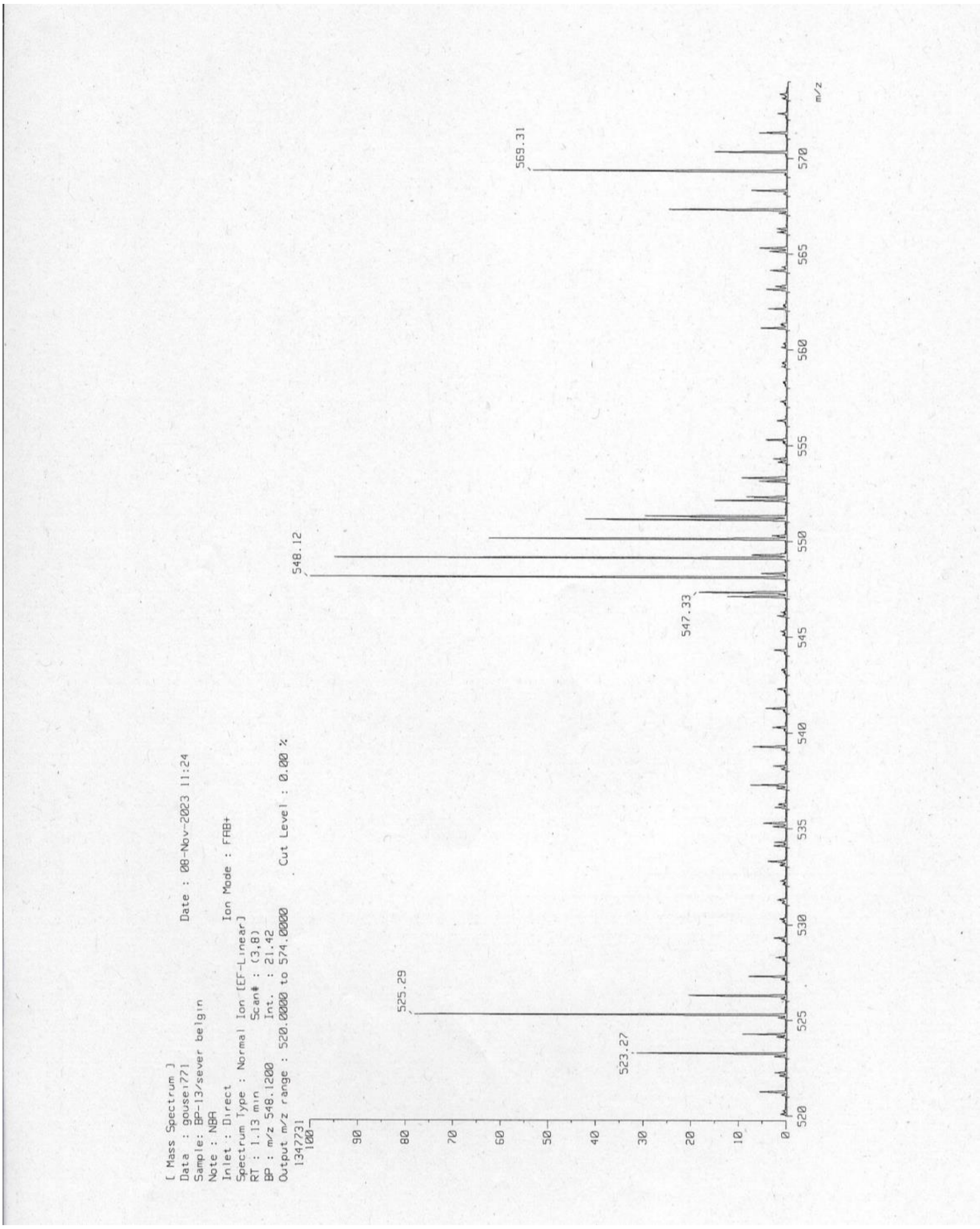
[Mass Spectrum]
Data : gouse1771
Sample: BP-13/sever belgin
Note : NBA
Inlet : Direct
Ion Mode : FAB+
Spectrum Type : Normal Ion [EF-Linear]
RT : 1.13 min
Scan# : (3,8)
BP : m/z 548.1200
Int. : 21.42
Output m/z range : 520.0000 to 574.0000
Cut Level : 0.00 %
Date : 08-Nov-2023 11:24
134723
523.27
525.29
547.33
548.12
569.31
m/z

Figure S52: ^{1}H NMR Spectrum of **BP-14**

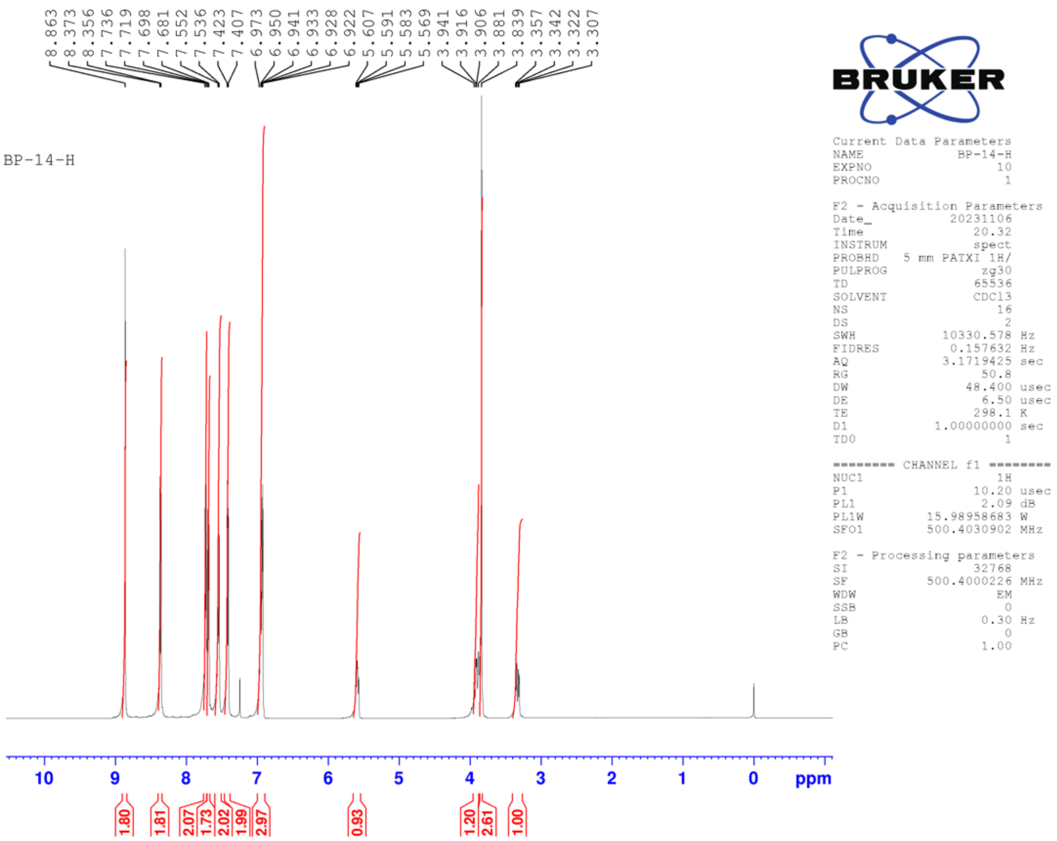

Figure S53: ^{13}C NMR Spectrum of **BP-14**

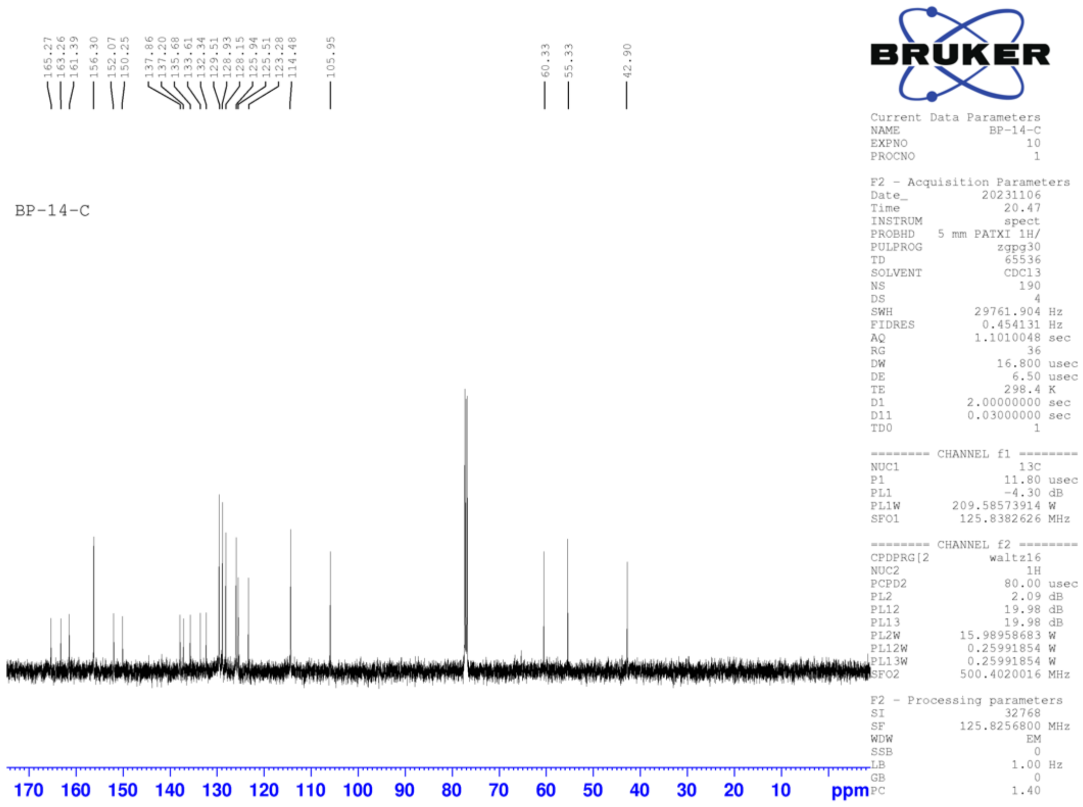

Figure S54: Mass Spectrum of **BP-14**

[Elemental Composition] Page: 1
Data : gousei772 Date : 08-Nov-2023 11:45
Sample: BP-14/sever belgin
Note : NBA
Inlet : Direct Ion Mode : FAB+
RT : 1.88 min Scan#: (6,11)
Elements : C 100/0, H 100/0, O 2/0, N 6/4, Cl 2/0, F 4/2, S 2/0
Mass Tolerance : 5ppm, 10mmu if m/z > 2000
Unsaturation (U.S.) : -0.5 - 100.0

Observed m/z	Int%	Err[ppm / mmu]	U.S.	Composition
591.1130	97.0	-3.1 / -1.8	25.5	C 32 H 18 O 2 N 6 Cl F 2
		+3.8 / +2.3	31.0	C 36 H 13 N 5 F 4
		+4.6 / +2.7	26.5	C 33 H 18 O 2 N 4 F 3 S
		+3.8 / +2.2	22.0	C 30 H 21 O N 5 Cl F 3 S
		+3.0 / +1.7	17.5	C 27 H 24 N 6 Cl 2 F 3 S
		-1.9 / -1.1	27.0	C 33 H 17 N 5 F 4 S
		+1.8 / +1.1	18.0	C 27 H 22 O 2 N 5 Cl F 4 S
		+1.0 / +0.6	13.5	C 24 H 25 O N 6 Cl 2 F 4 S
		+0.8 / +0.5	26.5	C 33 H 21 O N 4 F 2 S 2
		+0.0 / +0.0	22.0	C 30 H 24 N 5 Cl F 2 S 2
		+3.7 / +2.2	13.0	C 24 H 29 O 2 N 5 Cl 2 F 2 S 2
		-1.1 / -0.7	22.5	C 30 H 22 O 2 N 4 F 3 S 2
		-1.9 / -1.1	18.0	C 27 H 25 O N 5 Cl F 3 S 2
		-2.8 / -1.6	13.5	C 24 H 28 N 6 Cl 2 F 3 S 2
		-3.9 / -2.3	14.0	C 24 H 26 O 2 N 5 Cl F 4 S 2
		-4.7 / -2.8	9.5	C 21 H 29 O N 6 Cl 2 F 4 S 2
592.1192	100.0	+1.1 / +0.7	30.5	C 36 H 14 N 5 F 4
		+4.8 / +2.9	21.5	C 30 H 19 O 2 N 5 Cl F 4
		+4.0 / +2.4	17.0	C 27 H 22 O N 6 Cl 2 F 4
		+3.8 / +2.3	30.0	C 36 H 18 O N 4 F 2 S
		+3.0 / +1.8	25.5	C 33 H 21 N 5 Cl F 2 S
		+1.9 / +1.1	26.0	C 33 H 19 O 2 N 4 F 3 S
		+1.1 / +0.6	21.5	C 30 H 22 O N 5 Cl F 3 S
		+0.2 / +0.1	17.0	C 27 H 25 N 6 Cl 2 F 3 S
		-4.6 / -2.7	26.5	C 33 H 18 N 5 F 4 S
		-0.9 / -0.5	17.5	C 27 H 23 O 2 N 5 Cl F 4 S
		-1.7 / -1.0	13.0	C 24 H 26 O N 6 Cl 2 F 4 S
		-1.9 / -1.1	26.0	C 33 H 22 O N 4 F 2 S 2
		-2.7 / -1.6	21.5	C 30 H 25 N 5 Cl F 2 S 2
		+1.0 / +0.6	12.5	C 24 H 30 O 2 N 5 Cl 2 F 2 S 2
		-3.8 / -2.3	22.0	C 30 H 23 O 2 N 4 F 3 S 2
		-4.6 / -2.7	17.5	C 27 H 26 O N 5 Cl F 3 S 2

[Theoretical Ion Distribution] Page: 1
Molecular Formula : C30 H22 O N5 Cl F3 S
(m/z 592.1186, MW 593.0518, U.S. 21.5)
Base Peak : 592.1186, Averaged MW : 593.0518(a), 593.0540(w)

m/z	INT.	
592.1186	100.0000	**
593.1215	36.3612	**********************
594.1169	43.0281	**************************
595.1190	14.0094	********
596.1179	3.9091	**
597.1181	0.8080	
598.1192	0.1247	
599.1206	0.0152	
600.1221	0.0015	
601.1239	0.0001	

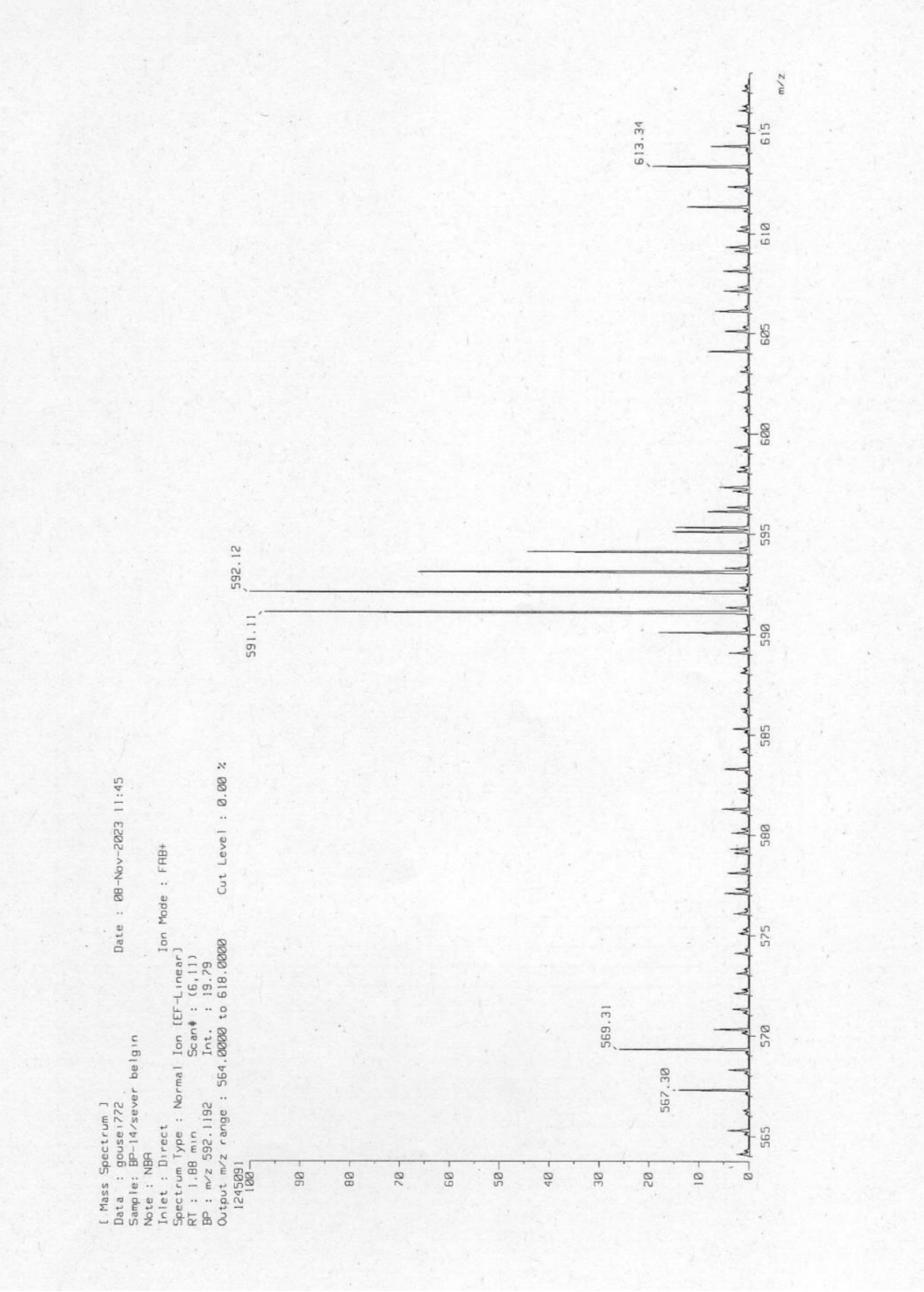
[Mass Spectrum]
Data : gousei772
Date : 08-Nov-2023 11:45
Sample: BP-14/sever belgin
Note : NBA
Inlet : Direct
Ion Mode : FAB+
Spectrum Type : Normal Ion [EF-Linear]
RT : 1.88 min
Scan# : (6,11)
BP : m/z 592.1192
Int. : 19.79
Output m/z range : 564.0000 to 618.0000
Cut Level : 0.00 %
1245091
567.30
569.31
591.11
592.12
613.34
m/z
